# Combined Experimental and Ab Initio Methods for Rationalization of Magneto-Luminescent Properties of Yb^III^ Nanomagnets Embedded in Cyanido/Thiocyanidometallate-Based Crystals

**DOI:** 10.1021/acs.jpclett.1c02942

**Published:** 2021-10-25

**Authors:** Jakub J. Zakrzewski, Kunal Kumar, Mikolaj Zychowicz, Robert Jankowski, Maciej Wyczesany, Barbara Sieklucka, Shin-ichi Ohkoshi, Szymon Chorazy

**Affiliations:** † Faculty of Chemistry, Jagiellonian University, Gronostajowa 2, 30-387 Krakow, Poland; ‡ Department of Chemistry, School of Science, The University of Tokyo, 7-3-1 Hongo, Bunkyo-ku, Tokyo 113-0033, Japan

## Abstract

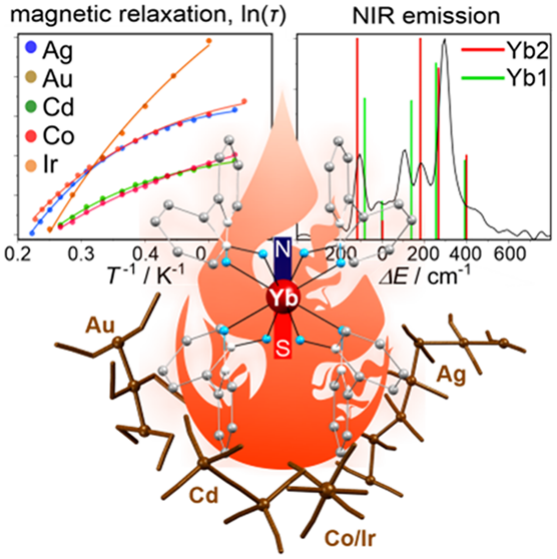

The *ab initio* calculations were correlated with magnetic and emission characteristics to understand the modulation of properties of NIR-emissive [Yb^III^(2,2′-bipyridine-1,1′-dioxide)_4_]^3+^ single-molecule magnets by cyanido/thiocyanidometallate counterions, [Ag^I^(CN)_2_]^−^ (**1**), [Au^I^(SCN)_2_]^−^ (**2**), [Cd^II^(CN)_4_]^2–^/[Cd^II^_2_(CN)_7_]^3–^ (**3**), and [M^III^(CN)_6_]^3–^ [M^III^ = Co (**4**), Ir (**5**), Fe (**6**), Cr (**7**)]. Theoretical studies indicate easy-axis-type ground doublets for all Yb^III^ centers. They differ in the magnetic axiality; however, transversal *g*-tensor components are always large enough to explain the lack of zero-dc-field relaxation. The excited doublets lie more than 120 cm^–1^ above the ground one for all Yb^III^ centers. It was confirmed by high-resolution emission spectra reproduced from the *ab initio* calculations that give reliable insight into energies and oscillator strengths of optical transitions. These findings indicate the dominance of Raman relaxation with the power *n* varying from 2.93(4) to 6.9(2) in the **4**–**3**–**5**–**1**–**2** series. This trend partially follows the magnetic axiality, being deeper correlated with the phonon modes schemes of (thio)cyanido matrices.

The development of lanthanides’ chemistry is driven by applications in life and technology.^1,2^ The latter is related to their emission originating from f–f electronic transitions.^3^ Lanthanide luminescent systems are promising for display devices, light-emitting diodes, optical communication, thermometry, sensing, and bioimaging.^4−8^ Their unique electronic properties related to spin–orbit coupling and crystal-field effects results also in strong magnetic anisotropy leading to the slow relaxation of magnetization.^9,10^ Resulting single-molecule magnets (SMMs) reveal magnetic hysteresis loop of a molecular origin arousing interest for data storage, quantum computing, and spintronics.^11−13^ The main issue is to take control over a large number of magnetic relaxation pathways.^14^ Among them, the Orbach process, quantum tunneling of magnetization (QTM), and Raman relaxation operate without the external field, while a direct process is recognized for field-induced SMMs. Strategies for the increased energy barrier of the Orbach process and eliminating QTM were broadly studied.^15−17^ In contrast, the two-phonon Raman relaxation is much harder to rationalize. Therefore, finding the means of doing so is of high interest in the field.^18−20^ Lately, the ideas of luminescent,^21^ conducting,^22^ or multifunctional SMMs^23,24^ have emerged, and the systems linking slow magnetic relaxation with porosity^25,26^ or photochromism^27^ were recognized. Lanthanide SMMs were lately applied in optical thermometry,^28,29^ while their emission spectra are usually used to perform magneto-optical correlations.^30^ The construction of such molecular materials may involve coordination chemistry.^18−30^ In this context, the application of cyanido metal complexes is efficient.^31,32^ These anionic moieties combined with d- and f-block metal ions resulted in coordination systems showing magnetic,^33^ optical,^33^ electric,^34^ and mechanical phenomena.^35^ They can enhance f-centered emission and induce their strong magnetic anisotropy.^36−39^ They also give information on how the exchange of d-block metal ions can affect the optical and magnetic properties of attached lanthanides.^29,40,41^ To better understand such modulation of emissive SMMs, we focused on supramolecular networks built of cationic Ln^III^ complexes and noncovalently bonded cyanido moieties. We used 2,2′-bipyridine-1,1′-dioxide (2,2′-bpdo) ligands together with Yb^3+^ ions that exhibit NIR emission and magnetic relaxation, usually controlled by phonon-assisted Raman processes.^42,43^ We aimed to combine the *ab initio* calculations with experimental magnetic and luminescent characterization to get insight into modifications of emissive [Yb^III^(2,2′-bpdo)_4_]^3+^ SMMs by the crystallization with (thio)cyanidometallate counterions. Herein, we report structures, magnetic and luminescent properties of the series of d–f Yb^III^-cyanido/thiocyanido ionic crystals (**1**–**7**) showing magnetic relaxation and NIR emission governed by the d-block metal ions, which was rationalized by the combined approach based on experimental studies confronted with the *ab initio* calculations playing a key role in the elucidation of both magnetic anisotropies as well as emission patterns.

Reported compounds crystallize from the solutions containing Yb^3+^ ions, 2,2′-bpdo ligands, and (thio)cyanido precursors, [Ag^I^(CN)_2_]^−^ (**1**), [Au^I^(SCN)_2_]^−^ (**2**), [Cd^II^(CN)_4_]^2–^/[Cd^II^_2_(CN)_7_]^3–^ (**3**), and [M^III^(CN)_6_]^3–^ [M^III^ = Co (**4**), Ir (**5**), Fe (**6**), Cr (**7**)] (see Experimental section in Supporting Information (SI)). The samples of **1**–**7** were characterized by IR spectra, TG, elemental analyses, and X-ray diffraction methods (Figures S1–S8, Tables S1–S7). All compounds crystallize as supramolecular frameworks built of cationic [Yb^III^(2,2′-bpdo)_4_]^3+^ complexes separated by anionic (thio)cyanido complexes and solvent (Figures 1 and S3–S7). The Yb^III^ units in **1**–**3** reveal the geometry of a square antiprism; however, the degree of distortion varies within the series, while the Yb^III^ centers in **4**–**7** adopt the intermediate geometry between a square antiprism and a dodecahedron (Table S7). The cyanido counterions are arranged through solvent-mediated hydrogen bonds (**4**–**7**); they form molecular aggregates based on metallophilic interactions (**1**, **2**); or they *in situ* form [Cd^II^_2_(CN)_7_]^3–^ units accompanying [Cd^II^(CN)_4_]^2–^ ions (**3**). The Yb^III^ complexes are arranged in a tubular manner.

**Figure 1:**
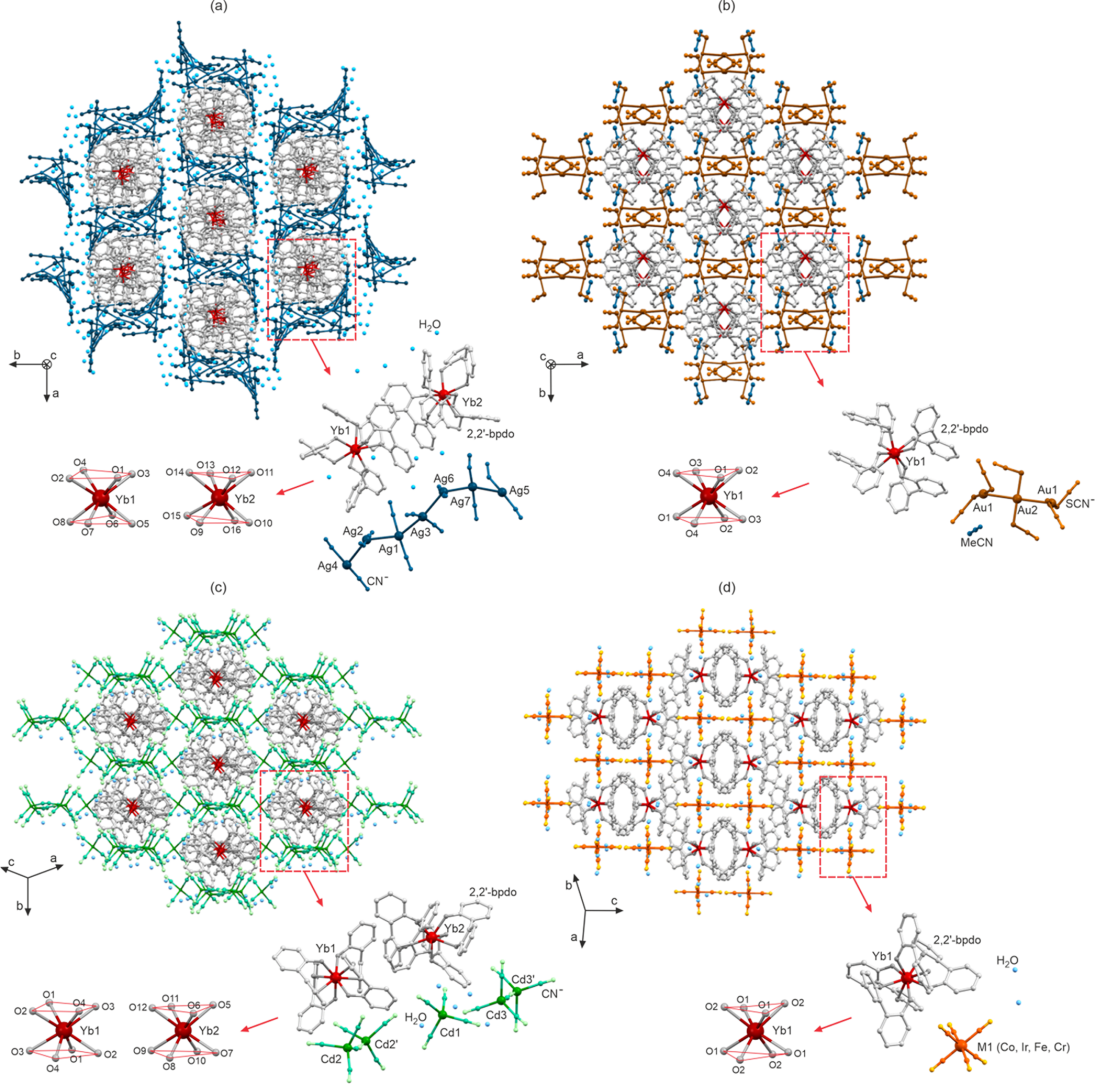
Crystal structures of **1** (a), **2** (b), **3** (c), and **4**–**7** (d) including tubular arrangements of Yb^III^ complexes, the molecular building units, and detailed view of Yb^III^ coordination spheres.

All compounds were studied using direct- (dc) and alternate-current (ac) magnetic measurements (Figures 2 and S9–S22). At 300 K, for **1**–**5** with diamagnetic d-block metal complexes, the χ_M_*T* product lies in the range of 2.47–2.54 cm^3^ mol^–1^ K expected for isolated Yb^3+^ ions (Figures S9–S10). For **6** and **7**, the respective values of 2.84 and 4.34 cm^3^ mol^–1^ K are enlarged because of the contributions from Fe^III^ or Cr^III^ centers, respectively. On cooling, the χ_M_*T* decreases for all cases due to the thermal depopulation of *m*_J_ levels within the ground multiplet. For **1**–**5**, or even for **6** and **7**, no distinct magnetic interactions are present. This claim is supported by *M*(*H*) curves at 1.8 K, showing a featureless increase of magnetization (*M*). At 50 kOe, the *M* values are in the 1.45–1.71 μ_B_ range for **1**–**5**, while for **6** and **7**, the values of 2.50 and 4.50 μ_B_ were found, respectively. The materials do not show a *M*(*H*) hysteresis loop even at 1.8 K owing to the moderate magnetic anisotropy of Yb^III^.^44^ Compounds **1**–**7** were characterized by field-variable ac magnetic studies (Figures S11–S22). Without the dc field, none of them shows a notable signal in the χ_M_″(ν) plots. When increasing the field, the appearance of the maxima occurs for **1**–**5**. They are initially slightly shifted to lower frequencies, but higher fields facilitate magnetic relaxation. Such behavior is due to the quenching of QTM and the appearance of a direct relaxation. Optimal dc fields were selected to follow the *T*-dependences of relaxation times (Figures 2 and S11–S20). For **1**–**5**, the maxima in the χ_M_″(ν) plots appear in the narrow *T–*range, which implies the strong *T*-dependences of relaxation times. To extract their values, a generalized Debye model was used.^45^ Field*-* and *T-*dependences were simultaneously fitted using the eq 1:
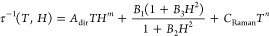
1where the first term corresponds to a direct process, the second to the QTM, while the last to the Raman relaxation.^46^ The term related to an Orbach process was excluded on the basis of the *ab initio* calculations and emission spectra (see below).^42^ However, for comparison, the formalism of an effective energy barrier (*U*_eff_) was applied in the highest recorded *T*-regime:
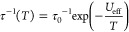
2

**Figure 2:**
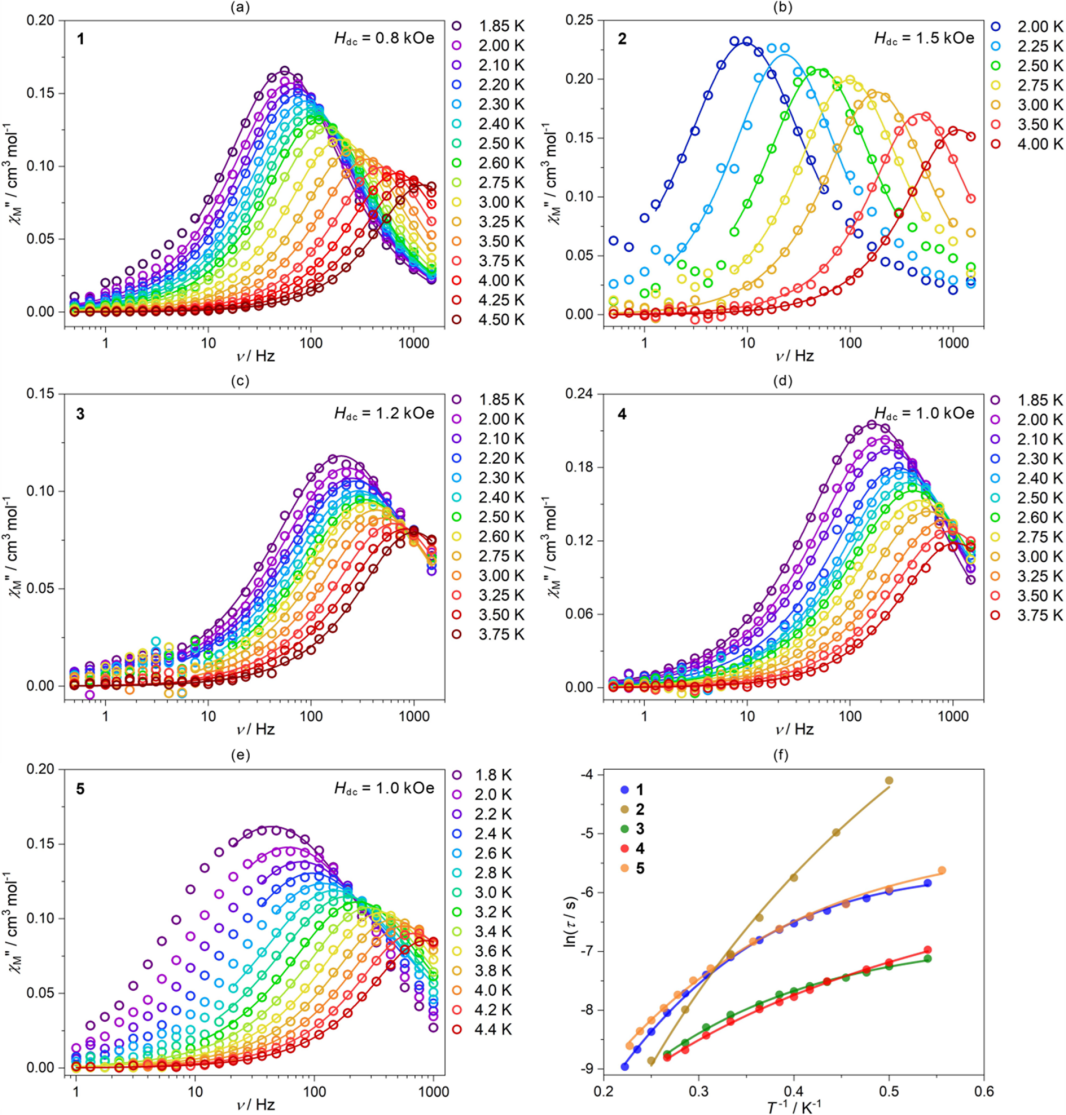
Alternate-current (ac) magnetism of **1**–**5**: the frequency dependences of the χ_M_″ (*H*_ac_ = 3 Oe) with the best-fits to the generalized Debye model (a–e), and *T*-variable relaxation times fitted using the contributions from Raman, direct and QTM processes (f) (Table 1). The circle points show the experimental data while the solid lines represent the respective fits.

Final parameters of *T*-dependences of the relaxation times are gathered in Tables 1 and S8. In all cases, the power *n* of Raman relaxation is lowered from the expected value of 9, which appears when not only acoustic but also optical phonons operate in spin–lattice relaxation.^18−20,47,48^ The best-fit parameters indicate the dominant role of the Raman relaxation. The power *n* is the highest (6.9) for the [Au^I^(SCN)_2_]^−^-based **2**; it is followed by **1** built of [Ag^I^(CN)_2_]^−^ anions, *n* = 4.92(5), and Ir^III^-based **5**, where *n* = 4.06(6). Much smaller *n* parameters of 3.43(5) and 2.93(4) were found for **3** and **4**, involving Cd^II^ and Co^III^, respectively. The *C*_Raman_ follow the opposite trend starting from 0.50(8) s^–1^K^–*n*^ for **2**, and reaching 131(5) s^–1^K^–*n*^ in **4**. As all compounds are based on [Yb^III^(2,2′-bpdo)]^3+^ complexes, the variation in Raman relaxation can be primarily ascribed to the phonon modes of transition metal complexes. The increasing trend of the power *n* in the series of **4**–**3**–**5**–**1**–**2** can be correlated with energies of key phonon modes.^18−20,47,48^ Their lowest energies giving the strongest *T*^n^ dependence are offered by [Au(SCN)_2_]^−^ ions (**2**). Higher energies of key phonon modes were found for [Ag(CN)_2_]^−^ ions (**1**) also forming supramolecular aggregates as [Au(SCN)_2_]^−^ ions, and for heavy [Ir^III^(CN)_6_]^3–^ ions (**5**). Lighter Cd^II^-cyanido units of **3** show much lower power *n* while the lowest is detected for the lightest [Co^III^(CN)_6_]^3–^ ions. The opposite trend of *C*_Raman_ parameter suggests that the number of accessible phonons is the highest for [Co^III^(CN)_6_]^3–^ while the lowest for [Au^I^(SCN)_2_]^−^ ions. It is also important to mention that a relatively high value of 6.9(2) for **2** suggests that almost only acoustic phonons are involved in the relaxation while, for others, optical phonons contribute to the relaxation. In the latter case, the crucial role of optical phonons has to be caused by the significantly stronger spin-phonon coupling than for acoustic phonons as relaxation appears at the low *T*-range where optical phonon modes are not easily accessible.^18−20,47,48^ Materials with paramagnetic [M^III^(CN)_6_]^3–^ ions reveal a χ_M_″(ν) signal, but it is only the onset of relaxation (**6**) or the low-frequency maxima of a dipolar origin (**7**) (Figures S21–S22).^40^

**Table 1:** Best-Fit Parameters for Relaxation Processes (direct, QTM, and Raman) Fitted for the *T*-Dependences of the Relaxation Times in **1**–**5**, Equation 1, in the Simultaneous Fitting of Both *H-* and *T*-Variable ac Magnetic Data, and the Parameters from the Linear Fitting of High *T*-Range Data, Equation 2

fitting type	parameter	**1**	**2**	**3**	**4**	**5**
overall fitting of direct, QTM, and Raman relaxation processes (eq 1)	*A*_dir_/s^–1^K^–1^Oe^–*m*^	2.11(2)·10^–12^	5.03(6)·10^–13^	5(1)·10^–13^	2.3(6)·10^–3^	2.7(1)·10^–13^
	*m*	4 (fixed)	4 (fixed)	4 (fixed)	1.59(3)^a^	4 (fixed)
	*B*_1_/s^–1^	497(14)	2000 (fixed)	2861(158)	2365(115)	190(8)
	*B*_2_/Oe^2–^	2.3(4)·10^–5^	5(1)·10^–4^	2.2(3)·10^–6^	4.8(6)·10^–5^	1.8(3)·10^–7^
	*B*_3_/Oe^2–^	1.1(1)·10^–5^	–	1.0(4)·10^–7^	–	–
	*C*_Raman_/s^–1^K^–*n*^	4.4(3)	0.50(8)	58(3)	131(5)	12.1(9)
	*n*	4.92(5)	6.9(2)	3.43(5)	2.93(4)	4.06(6)
Arrhenius dependence fitting (eq 2)	*U*_eff_/K	20.6(5)	22(1)	10.2(5)	9.4(5)	18(1)
	τ_0_/s	1.4(2)·10^–6^	7(2)·10^–7^	1.0(2)·10^–5^	1.2(2)·10^–5^	3(1)·10^–6^

a For **4**, it was impossible to obtain a reliable fit by fixing the typical *m* direct parameter of 4. Its low value is usually rationalized by the role of hyperfine interactions,^15^ but here, the broadening of the χ_M_″(ν) maxima at high dc fields affects the extracted relaxation times thus also direct parameters within the overall fitting.

Compounds **1**–**7** exhibit solid-state NIR luminescence at 300 K (Figures 3 and S24–S25). The shape of the ^2^F_5/2_ → ^2^F_7/2_ band slightly varies because of subtle differences in the Yb^III^ complexes. The excitation spectra are divided into two ranges. Above ca. 900 nm, there is a band related to the direct f–f excitation, while below ca. 500 nm all compounds reveal the excitation band caused by the energy transfer (ET) from 2,2′-bpdo ligand.^39^ Within **1**–**5**, there are small differences in the structure of this band related to admixtures of ET from (thio)cyanidometallates. In **6**, the intensity of the 2,2′-bpdo excitation is smaller than direct excitation as [Fe^III^(CN)_6_]^3–^ ions harvest the energy from organic ligands.^36^ For the [Cr^III^(CN)_6_]^3–^-based **7**, the excitation spectrum shows an additional band at the edge of UV–vis ranges. It can be assigned to the Cr^III 4^A_2g_ → ^4^T_2g_ transition, thus the Cr^III^-to-Yb^III^ ET.^36,37^ After being cooled, the shapes of the excitation spectra barely change, with the only strong difference in **6** where 2,2′-bpdo-centered bands dominate at low-*T*. Pronounced differences are seen in the emission due to the partial disappearance of hot bands.

**Figure 3:**
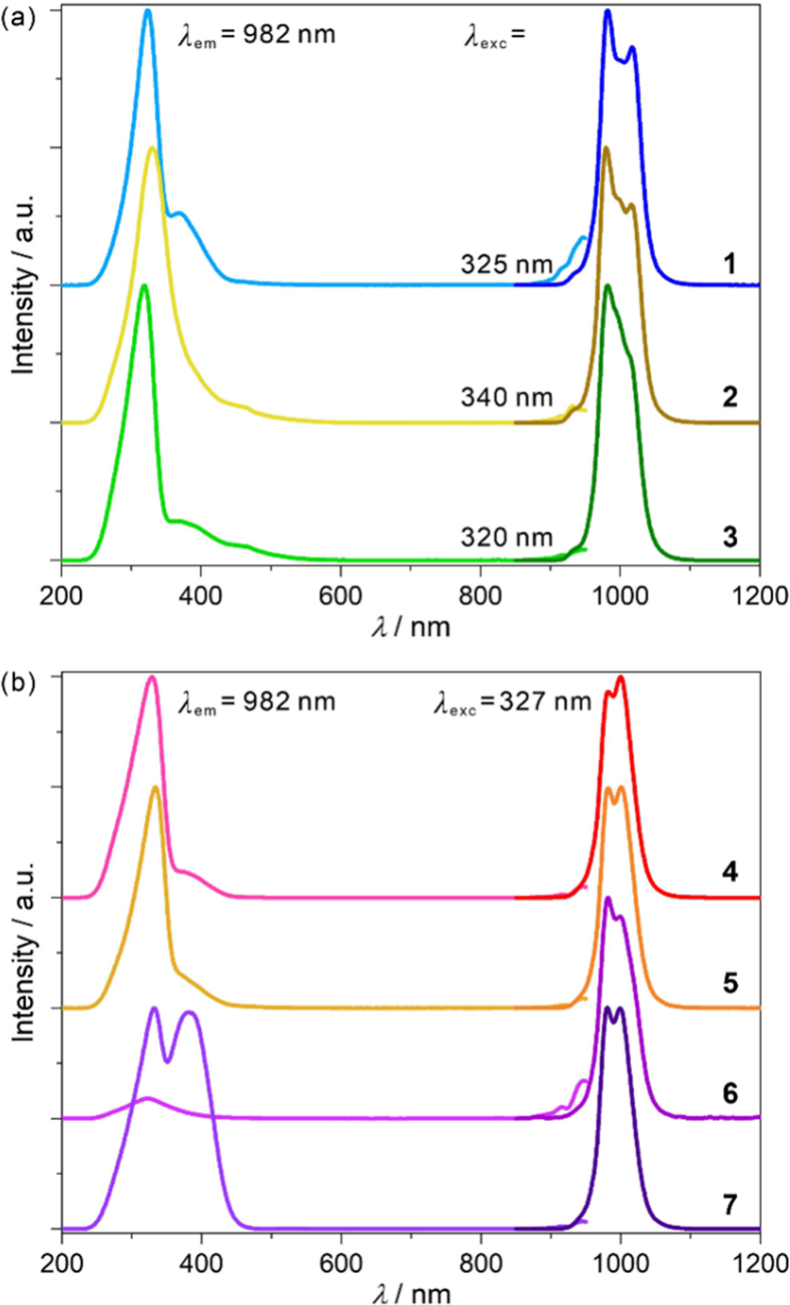
Solid-state photoluminescence of **1**–**3** (a) and **4**–**7** (b), including the excitation and the emission spectra at the indicated wavelengths measured at room temperature.

The *ab initio* calculations of a CASSCF/RASSI/SINGLE_ANISO type were performed on the structural models of **1**–**7** (Tables S9–S17, Figures 4–5, S23).^15^ The calculations were done using two basis sets (models **S** and **L**; Table S9). For Yb1 center of **1**, the active space was additionally enlarged to check the influence of increased orbital mixing (model **L+**). The resulting energy splitting, the composition of the ground doublets, and pseudo-*g*-tensor components for calculated complexes are gathered in Tables S10–S16. The ground Kramers doublets for all computed Yb^III^ centers were found to be an eas*y*-axis type; however, the alignment of a magnetic easy axis (the *g*_*z*_ component) varies in the **1**(Yb1/Yb2)–**2**–**4**–**5** series (Figure 4). This can be explained by the non-negligible structural differences between Yb centers crystallizing in various space groups with the distinguishable supramolecular environment of (thio)cyanido metal complexes. Moreover, the Yb complexes differ in the strength of magnetic axiality represented by the pseudo-*g*-tensor components. The strongest axiality of the highest *g*_*z*_ and the lowest transversal components was found for the Yb1 complex of **2**, but even for this case, the *g*_*x*_ and *g*_*y*_ factors are above 0.15, which explains the lack of zero-dc-field magnetic relaxation. Weaker axiality is observed in Yb1/Yb2 centers of **1**, and the weakest for Yb1 units of isostructural **4** and **5**. One can notice that the strength of the *T*-variation of Raman relaxation, represented by the power *n*, increases together with the increased magnetic axiality. However, this factor seems to play a supporting role as shown by **4** and **5** revealing almost identical ground states but very different Raman relaxation (Table 1). Thus, the correlation between the Raman process and the phonon modes scheme of the lattice, discussed above, plays a dominant role. The results of *ab initio* calculations of the **L** model indicate that the first excited Kramers doublets for all calculated Yb centers lie more than 130 cm^–1^ above the ground one, which excludes the Orbach relaxation process as such a high energy barrier cannot be used to reproduce the relaxation dynamics; for example, the effective energy barriers for the high *T*-range are smaller than 16 cm^–1^ (22 K, Table 1). This confirms the supremacy of Raman relaxation in **1**–**5**. There is no noticeable correlation between the energies of excited Kramers doublets and the parameters of Raman relaxation, for example, **2**, showing the highest power *n*, exhibits the intermediate values of energy splitting. The quality of the calculations is documented by the dc magnetic curves (Figures S9–S10). Theoretical χ_M_*T*(*T*) plots for **4** and **5** reveal noticeable discrepancies from the experiment, however, these differences are comparable with other reported cases of Yb^III^ SMMs.^24,42,44^

**Figure 4:**
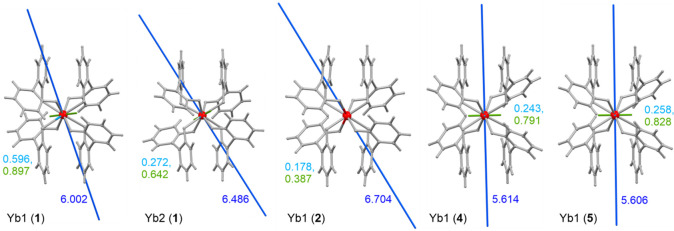
Alignment of the main magnetic axes representing the pseudo-*g*-tensor components obtained from the *ab initio* calculations for the ground doublets of Yb^III^ centers in **1**, **2**, **4**, and **5**.

**Figure 5:**
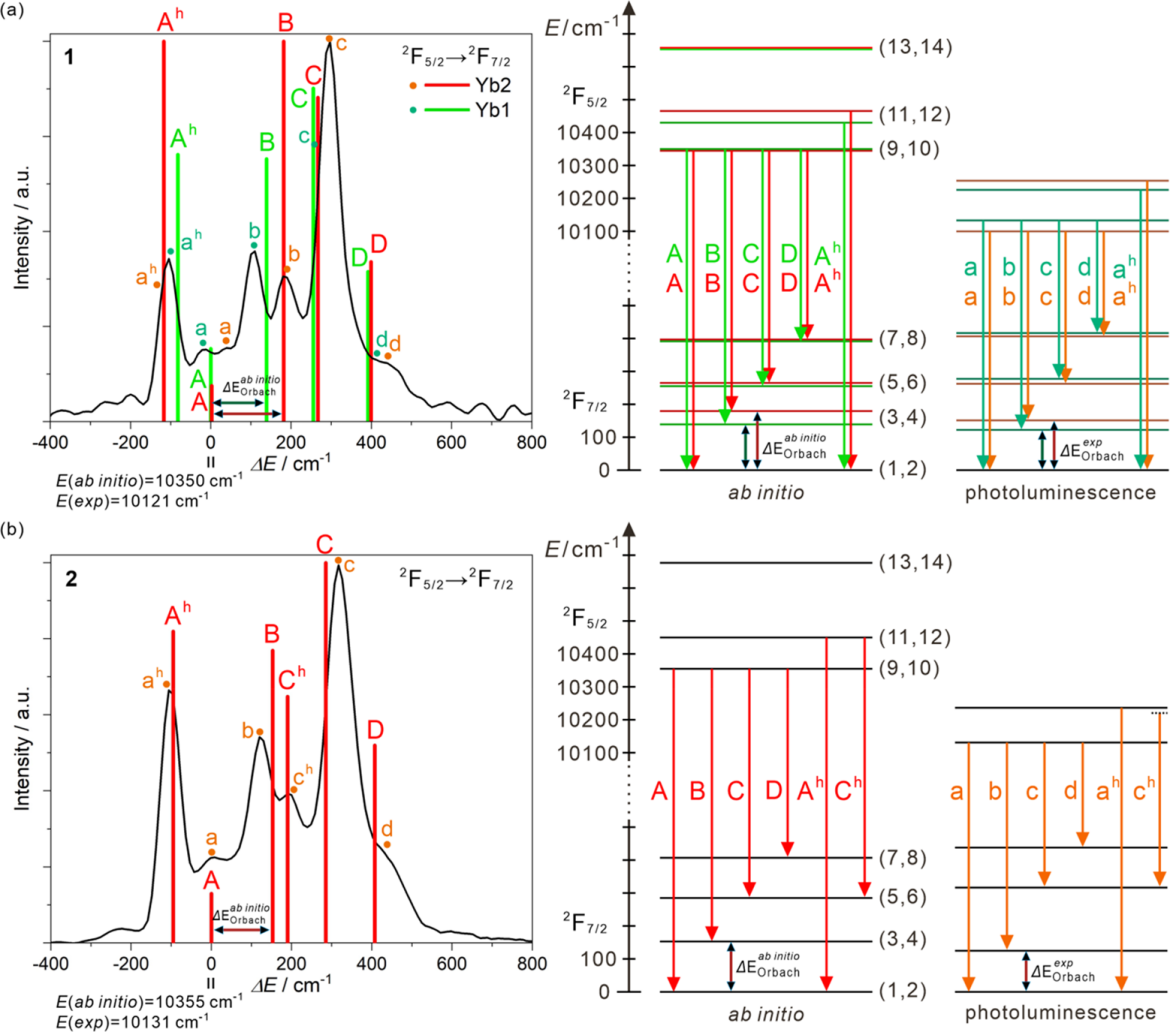
High-resolution emission spectra of **1** (a) and **2** (b) at 80 K for the 325 nm excitation (black lines), shown with the cumulative oscillator strengths (colored bars) obtained from the *ab initio* calculations. The spectra are presented in the function of energy differences (Δ*E*) counted in relation to the calculated 0–0 emission line (A). For better comparison, the spectra were repositioned to have the identical zero point and the corresponding absolute energy values are indicated. The positive values of Δ*E* represent the transition energies smaller than the 0–0 line; thus, they relate to the energy splitting of the ground multiplet, while the negative values of Δ*E* show the hot bands (marked with *h*). The right panel shows the comparison of the energy level diagrams obtained from the *ab initio* and the experimental emission spectra.

The results of the *ab initio* calculations were used to rationalize the high-resolution emission spectra gathered at 80 K for **1**, **2**, **4**, and **5**. By taking advantage of the RASSI module of OpenMolcas, it is achievable to calculate transition moments between spin–orbit states obtained after diagonalization of AMFI spin–orbit Hamiltonian. From this, we can obtain oscillator strength for the transition from the energy state A to B which is represented by factors expressing the probability of emission, thus also emission band intensity. These factors are defined by eq 3:
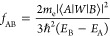
3where *E*_B_ and *E*_*A*_ are energies of state B and A, respectively, and ⟨*A*|*W*|*B*⟩ is a transition moment between these two states. This method relies on two approximations. The first is the application of states taken from the state average multiconfigurational calculations considering relativistic effects but done on the experimental geometry, not the geometry for an excited state. This can be justified by the small impact of changing geometry on the crystal field for well-screened 4f electrons. The second is the consideration of only electric-dipole transitions, but it is sufficient to explain intensities as shown by the Judd–Ofelt Theory of intensities (see discussion in the SI). In this approach, for A and B energy states which are exact solutions to the time-dependent Schrödinger equation with Hamiltonian, *H*, and for an arbitrary operator *W*, the eq 4 holds:
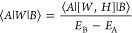
4

Thus, there exists an arbitrary choice of the operator. To calculate dipole transition moment where operator *W* is simply a vector of (*x,y,z*) coordinates, we can also use [*W*,*H*] which for nonrelativistic Hamiltonian is a velocity operator. These two approaches are identical as far as the used functions are exact solutions, but with the approximated functions in practical calculations, they differ. We found that the velocity gauge more correctly reproduces experimental spectra. The calculated cumulative strengths, which are obtained by the 2 × 2 summation over Kramers doublets of energy states *A* and *B* taken from the **L/L+** models are shown in Table S17. The obtained results were presented in the comparison with the experimental spectra which were repositioned to correspond to the relative energies from calculations (see Figures 5 and S26 with the comments in the captions). The *ab initio* calculations with our approach well reproduce not only the energies but also relative intensities of emission bands. The latter can be now clearly assigned to the specific transitions between the doublets of the emissive and ground multiplets. This is particularly helpful for the detection of the 0–0 emission line and the separation of the hot bands, two issues that are critical for magneto-luminescent correlations in SMMs.^21,23,24,28,30,42^ The *ab initio* calculations are even useful for the estimation of the energy of emissive multiplet, only slightly overestimating the related energies by less than 2%, being also appropriate for the determination of the detailed energy splitting of ground multiplets (Table S18). Then, we can undoubtedly determine the energy gaps between the two lowest-lying Kramers doublets which represent the energy barriers for a potential Orbach magnetic relaxation (Δ*E*_Orbach_, Figures 5 and S26, Table S18). The related theoretically estimated energy gaps (Δ*E*_*Orbach*_^*ab initio*^) are very close to the optically estimated values (Δ*E*_*Orbach*_^*exp*^), and all of them, within the whole set of investigated Yb complexes, were found to be above 120 cm^–1^. This confirms the lack of an Orbach relaxation in the accessible frequency range for the presented compounds which supports the conclusion of the dominant role of a Raman process. The optical estimation keeps the identical trend of the energy gaps in the **1**–**5** series as found from the *ab initio*, for example, the highest *ΔE*_Orbach_ is ascribable to Yb2 centers of **1**, confirming also that the energy schemes of doublets do not directly influence the efficiency of Raman relaxation as this trend does not follow those for the power *n*.

In conclusion, we present the methodology based on the *ab initio* calculations confronted with experimental magnetic and luminescent data that enables the deep investigation of physical properties of NIR-emissive [Yb^III^(2,2′-bpdo)]^3+^ nanomagnets embedded in the crystal lattices with diverse cyanido/thiocyanidometallates. First, the *ab initio* calculations were used to determine the character of Yb^III^ electronic ground states. They are of an easy-axis type with tunable magnetic axality within the series, but in all cases, the non-negligible transversal *g*-tensor components exist, explaining the lack of zero-dc-field magnetic relaxation. More importantly, the *ab initio* approach gave insight into the energy scheme both for the ground multiplet as well as for the emissive level. This leads to the successful reproduction of the emission spectra, including not only the energies of emission bands, which is typically explored,^21,23,24,28,30,42^ but also the bands’ intensities represented by the cumulative oscillator strengths proportional to transition moments computed using a velocity gauge. This is extremely helpful in the clear interpretation of emission patterns, in particular, in finding the 0–0 emission line and detecting the hot bands. As a result, we performed reliable magneto-luminescent correlations including the determination of energy gaps between two lowest-lying Kramers doublets. They, confronted with ac magnetic data, indicate that the SMM behavior in the whole series is dominated by the Raman relaxation. We observed the broad variation of the efficiency of Raman relaxation which is dependent on the (thio)cyanido counterion. This modulation partially follows the magnetic axiality of the Yb^III^ ground doublet which is related to subtle structural differences between lanthanide complexes induced by changing the crystal lattice by metal-based counterions. However, the main correlation exists between the *T*-dependence of a Raman process and the phonon modes scheme of the lattice. We found the increasing trend of the power *n* in the series with [Cd(CN)_*x*_]^*n*−^, [Ir(CN)_6_]^3–^, [Ag(CN)_2_]^−^, up to [Au(SCN)_2_]^−^ ions. This trend was assigned to the decreasing energies of the critical phonon modes, further correlated with the CN^–^ to SCN^–^ change, lighter-to-heavier metal substitution, and the formation of metallophilic aggregates. The *C*_Raman_ parameter reveals almost the opposite trend which suggests that the accessibility of key phonon modes can be governed by the opposite factors to those operating for their energies. Therefore, we show the great potential in using the *ab initio* calculations for the elucidation of magneto-luminescent properties of Yb^III^ complexes with the particular attention given to the challenging task of the calculation of emission spectra. Our approach is a convenient tool for the discussion of SMM effects in luminescent complexes but can be extended for the investigation of advanced luminescent phenomena such as optical thermometry utilizing the *T*-dependence of hot emission bands.^24,28^ This research direction as well as the expansion of the methodology to other lanthanide ions with more complex energy level diagrams are in progress.

## Supporting Information Available

Experimental section; basic characterization and detailed structural studies; complete dc and ac magnetic characteristics; details and expanded results of the *ab initio* calculations; additional luminescent characteristics (PDF)
X-ray data (CIF)

## Supplementary Material

jz1c02942_si_001.pdf

jz1c02942_si_002.cif
