# Supplementary material for: Combined Experimental and Ab Initio Methods for Rationalization of Magneto-Luminescent Properties of YbIII Nanomagnets Embedded in Cyanido/Thiocyanidometallate-Based Crystals
Source: J Phys Chem Lett. 2021 Oct 25;12(43):10558–66. doi: 10.1021/acs.jpclett.1c02942 (PMC8573772; doi:10.1021/acs.jpclett.1c02942)
Supplement: Supplementary file 1 — jz1c02942_si_001.pdf [file jz1c02942_si_001.pdf]

SUPPORTING INFORMATION

Combined Experimental and Ab Initio Methods for Rationalization of Magneto-Luminescent Properties of Yb^{III} Nanomagnets Embedded in Cyanido/Thiocyanidometallate-Based Crystals

Jakub J. Zakrzewski,[1] Kunal Kumar,[2] Mikolaj Zychowicz,[1] Robert Jankowski,[1]
Maciej Wyczesany,[1] Barbara Sieklucka,[1] Shin-ichi Ohkoshi,[2] and Szymon Chorazy[1,]*

[1]Faculty of Chemistry, Jagiellonian University, Gronostajowa 2, 30-387 Kraków, Poland. [2]Department of Chemistry, School of Science, The University of Tokyo, 7-3-1 Hongo, Bunkyo-ku, Tokyo 113-0033, Japan.

*Corresponding author: chorazy@chemia.uj.edu.pl

Experimental section. S3
Results of preliminary characterization and detailed structural studies. (comment) S6
Infrared absorption spectra of **1**–**7**. (Figure S1) S7
Thermogravimetric curves of **1**–**7**. (Figure S2) S8
Crystal data and structure refinement for **1–3**. (Table S1) S9
Crystal data and structure refinement for **4–7**. (Table S2) S10
Detailed structure parameters of ytterbium(III) complexes in **1**–**3**. (Table S3) S11
Detailed structure parameters of ytterbium(III) complexes in **4**–**7**. (Table S4) S12
Detailed structure parameters of cyanido metal complexes in **1**–**3**. (Table S5) S13
Detailed structure parameters of cyanido metal complexes in **4**–**7**. (Table S6) S14
Results of Continuous Shape Measure Analysis for $[Yb^{III}(2,2'\text{-bpdo})_4]^{3+}$ complexes in **1**–**7**. (Table S7) S15
The representative structural fragments of **1**. (Figure S3) S16
The detailed structural view of the helical supramolecular arrangement of metallophilic $[Ag^{I}(CN)_2]$-based chains around the $[Yb^{III}(2,2'\text{-bpdo})_4]^{3+}$ complexes in the crystal structure of **1**. (Figure S4) S17
The asymmetric unit of **2** presented with the atoms labeling scheme. (Figure S5) S18
The representative structural fragments of **3**. (Figure S6) S19
The asymmetric unit of **4–7** presented with the respective atoms labeling schemes. (Figure S7) S20
Powder X-ray diffraction patterns of **1–7**. (Figure S8) S21
Direct-current (*dc*) magnetic properties of **1**, **2**, and **3**. (Figure S9) S22
Direct-current (*dc*) magnetic properties of **4**, **5**, **6**, and **7**. (Figure S10) S23
Complete magnetic-field-variable alternate-current (*ac*) magnetic susceptibility characteristics of **1**. (Figure S11) S24
Complete temperature-variable alternate-current (*ac*) magnetic susceptibility characteristics of **1**. (Figure S12) S25
Complete magnetic-field-variable alternate-current (*ac*) magnetic susceptibility characteristics of **2**. (Figure S13) S26
Complete temperature-variable alternate-current (*ac*) magnetic susceptibility characteristics of **2**. (Figure S14) S27
Complete magnetic-field-variable alternate-current (*ac*) magnetic susceptibility characteristics of **3**. (Figure S15) S28
Complete temperature-variable alternate-current (*ac*) magnetic susceptibility characteristics of **3**. (Figure S16) S29
Complete magnetic-field-variable alternate-current (*ac*) magnetic susceptibility characteristics of **4**. (Figure S17) S30
Complete temperature-variable alternate-current (*ac*) magnetic susceptibility characteristics of **4**. (Figure S18) S31
Complete magnetic-field-variable alternate-current (*ac*) magnetic susceptibility characteristics of **5**. (Figure S19) S32
Complete temperature-variable alternate-current (*ac*) magnetic susceptibility characteristics of **5**. (Figure S20) S33
Field- and temperature-variable alternate-current (*ac*) magnetic susceptibility characteristics of **6**. (Figure S21) S34
Field- and temperature-variable alternate-current (*ac*) magnetic susceptibility characteristics of **7**. (Figure S22) S35
Comment to Figures S11–S20. S36
Summary of parameters extracted from the fitting of *H*- and *T*-dependences of the relaxation times in **1–5** including contributions to the overall relaxation time from all considered processes at the indicated conditions. (Table S8) S36

Description with contractions of the basis sets employed in *ab initio* calculations. (Table S9) S37
Summary of the *ab initio* calculations for Yb1 centers in **1** (models **S** and **L**). (Table S10) S37
Summary of the *ab initio* calculations for Yb2 centers in **1**. (Table S11) S38
Summary of the *ab initio* calculations for Yb1 centers in **1** (model **L** and **L+**). (Table S12) S38
Summary of the *ab initio* calculations for Yb^{III} centers in **2**. (Table S13) S39
Summary of the *ab initio* calculations for Yb^{III} centers in 4. (Table S14) S39
Summary of the *ab initio* calculations for Yb^{III} centers in **5**. (Table S15) S40
Energy splitting of ^2F term of Yb^{III} for **1**, **2**, **4**, and **5**. (Table S16) S41
Cumulative oscillator strengths for transitions between emissive doublet of $^2F_{5/2}$ excited term and four doublets of $^2F_{7/2}$ multiplet in velocity gauge together with most intensive hot transitions. (Table S17) S41
Comment to Tables S9–S17 (details of *ab initio* calculations) S42
Fragments of the crystal structures of **1**, **2**, **4**, and **5** used for ab initio calculations. (Figure S23) S43
Solid-state excitation and emission spectra of **1**, **2**, and **3**. (Figure S24) S44
Solid-state excitation and emission spectra of **4**, **5**, **6**, and **7**. (Figure S25) S45
High-resolution emission spectra of **4** and **5** at 80 K for the 325 nm excitation shown with the calculated cumulative oscillator strengths obtained from the *ab initio* calculations using the **L** models. (Figure S26) S46
Comparison of experimental and theoretical (obtained from the *ab initio* calculations) energies of emission transitions and energies of Kramers doublets of Yb^{III} complexes in **1**, **2**, **4**, and **5**. (Table S18) S47
References to Supporting Information. S48

EXPERIMENTAL SECTION

Materials

All the precursors required for the synthesis of $K_3[Ir(CN)_6]$ and $K_2[Cd(CN)_4]$, as well as those further applied for the syntheses of **1**–**7**, were purchased from commercial sources (Sigma-Aldrich, Tokyo Chemical Industries Co., Ltd., Wako Pure Chemicals Industries, Ltd, abcr GmbH) and used as received without further purification.

Synthesis and basic characterization

Synthesis of $K_2[Cd(CN)_4]$

The cyanido precursor for the synthesis of **3**, $K_2[Cd(CN)_4]$, was prepared using cadmium(II) chloride, $CdCl_2$, potassium hydroxide, KOH, and potassium cyanide, KCN, following a modified literature procedure.[1] An aqueous solution of KOH (1.23 g, 22 mmol, 20 mL) was added dropwise to an aqueous solution of $CdCl_2$ (2.02 g, 11 mmol, 15 mL) while stirring at room temperature. The resulting white precipitate was collected by suction filtration and then dissolved in 15 mL of the aqueous solution containing 3 g (46 mmol) of KCN. The obtained mixed solution was left undisturbed in an opened beaker for slow evaporation. The crystalline precursor was obtained after ca. 7 days, and it was separated by suction filtration from the remaining solution. Yield: 1.75 g, 54% (based on $CdCl_2$).

Synthesis of $K_3[Ir(CN)_6]$

The cyanido precursor for the synthesis of **5**, $K_3[Ir(CN)_6]$, was prepared using potassium hexachloroiridate(III), $K_3[IrCl_6]$, and potassium cyanide, KCN, following a modified literature procedure.[2] A 1 g portion of $K_3[IrCl_6]$ was milled together with 2 g of KCN and transferred to a glass tube, which was in the next step sealed under vacuum. The sealed tube was inserted into an oven and quickly heated up to 650°C, then kept for ca. 10 min, and allowed to cool down to room temperature. After opening the tube, the crude residue was treated with hot water (ca. 60°C, ca. 100 mL), and the resulting solution was filtrated and neutralized using diluted hydrochloric acid, HCl, to remove the excess of KCN (*caution: a large amount of volatile HCN is generated in this process*). The solution was evaporated to dryness using a rotatory evaporator. *The residue was redissolved in water (ca. 200 mL), and an ion exchanger, Dowex® 50W X8 (hydrogen form) was added keeping the whole mixture within a large beaker. Such prepared mixture was stirred for ca. 1 h, filtrated, and evaporated to dryness. The procedure starting with a mark (*) was repeated 3 to 4 times to guarantee a proper exchange of cations (from K^+ to H^+) and to fully remove KCl. After this step, $H_3[Ir(CN)_6]$ was obtained in form of white powder. It was again dissolved in water, and carefully neutralized using a diluted aqueous solution of KOH, then the solution was evaporated to dryness. The final product can be at this stage recrystallized by dissolving $K_3[Ir(CN)_6]$ in the minimal amount of water and treating the concentrated solution with a large excess of ethanol. Yield: 0.7 g, 79% (based on $K_3[IrCl_6]$).

*Synthesis of **1***

A 29.9 mg (0.15 mmol) portion of potassium dicyanoargentate, $K[Ag(CN)_2]$ in 0.5 mL of distilled water was added to the 1.5 mL portion of an aqueous solution containing 19.4 mg (0.05 mmol) of $YbCl_3·6H_2O$ and 37.6 mg (0.20 mmol) of 2,2'-bpdo (2,2'-bipyridine-1,1'-dioxide). The resulting mixture was stirred at room temperature for 5 min, and left open in the dark at 5°C for crystallization. The colorless crystals of **1** were obtained by slow evaporation after ca. 2 days. The composition of **1**, $[Yb^{III}(2,2'\text{-bpdo})_4][Ag^I(CN)_2]_3·7.5H_2O$ was determined by SC-XRD analysis. This formula is preserved when crystals are covered by the mother solution or by the protective grease, but on exposure to the air atmosphere, the samples of **1** were found to turn amorphous with the final water content of 4 molecules per {YbAg$_3$} formula unit, as established by the CHN elemental analysis and TG studies ($\mathbf{1}^{\mathbf{dried}}$). Yield (based on $\mathbf{1}^{\mathbf{dried}}$): 47% (34.7 mg). CHN elem. anal. calcd. for $Yb_1Ag_3C_{46}H_{40}N_{14}O_{12}$ (M_W = 1477.5 g·mol^{-1}): C, 37.1%; H, 2.7%; N, 13.3%. Found: C, 37.4%; H, 2.4%; N, 13.2%.

*Synthesis of **2***

A 53.0 mg (0.55 mmol) portion of potassium thiocyanide, KSCN was added to the 6 mL of an acetonitrile solution containing 80.0 mg (0.27 mmol) of chloro(dimethylsulfide)gold(I), and the resulting mixture was stirred at room temperature for 20 min. Then, the white precipitate formed in the reaction was removed by suction filtration, and the 6 mL of the filtrate was reduced to 3mL by air drying in the dark and transferred to the separate vial. This step was followed by the addition of 37.0 mg (0.096 mmol) of $YbCl_3·6H_2O$ dissolved in the 1.6 mL portion of a 3:1 $CH_3CN:H_2O$ mixture, and 57.0 mg (0.3 mmol) of 2,2'-bpdo dissolved in the 3 mL portion of a 1:2 $CH_3CN:H_2O$ mixture. The vial containing the final mixed solution was left closed in the dark for crystallization. The crystals of **2** appeared after ca. 2 days. The composition of **2**, $[Yb^{III}(2,2'\text{-bpdo})_4][Au^I(SCN)_2]_3·2MeCN$ was determined by SC-XRD analysis. This formula is preserved when crystals are covered by the mother solution or by the protective grease, but on exposure to the air atmosphere, one MeCN molecule per formula unit is removed. The resulting air-stable composition, $[Yb^{III}(2,2'\text{-bpdo})_4][Au^I(SCN)_2]_3·MeCN$ was given by the CHN analysis and the TG for an air-dried **2** ($\mathbf{2}^{\mathbf{dried}}$). Yield (based on $\mathbf{2}^{\mathbf{dried}}$ and Yb^{3+} precursor): 62% (108 mg). CHN elem. anal. calcd. for $Yb_1Au_3C_{48}H_{35}N_{15}O_8S_6$ (M_W = 1906.2 g·mol^{-1}): C, 30.2%; H, 1.9%; N, 11.0%. Found: C, 30.3%; H, 2.0%; N, 11.1%.

*Synthesis of **3***

A 71 mg (0.24 mmol) portion of potassium tetracyanidocadmate(II), $K_2[Cd(CN)_4]$ in 1 mL of distilled water was added to a 2.4 mL portion of an aqueous solution containing 68 mg (0.16 mmol) of $Yb(NO_3)_3 \cdot 5H_2O$ and 60 mg (0.32 mmol) of 2,2'-bpdo. The resulting mixture was stirred at room temperature for 10 s and left closed in the dark for crystallization. The colorless crystals of **3** were obtained after ca. 1 day. Then, the product was separated by suction filtration. The composition of **3**, $[Yb^{III}(2,2'\text{-bpdo})_4]_9[Cd^{II}(CN)_4]_6[Cd^{II}_2(CN)_7]_5 \cdot 44H_2O$ was determined by the SC-XRD analysis, and confirmed by the results of CHN elemental analysis and TG studies. Yield (based on Yb^{3+} precursor): 28% (124 mg). CHN elem. anal. calcd. for $Yb_9Cd_{16}C_{419}H_{376}N_{131}O_{116}$ (M_W = 12458.2 g·mol^{-1}): C, 40.4%; H, 3.0%; N, 14.7%. Found: C, 40.1%; H, 2.6%; N, 15.0%.

*Synthesis of **4***

A 39.9 mg (0.12 mmol) portion of potassium hexacyanidocobaltate(III), $K_3[Co(CN)_6]$ in 1 mL of distilled water was added to a 3 mL portion of an aqueous solution containing 46.5 mg (0.12 mmol) of $YbCl_3 \cdot 6H_2O$ and 180.7 mg (0.96 mmol) of 2,2'-bpdo. The resulting mixture was vigorously stirred at room temperature for 10 s. After ca. 2 minutes the crystalline product starts to appear in the vial, which was then closed and left in the dark for 2 days for crystallization. Then, the product was separated by suction filtration. The composition of **4**, $[Yb^{III}(2,2'\text{-bpdo})_4][Co^{III}(CN)_6] \cdot 6H_2O$ was determined by the SC-XRD analysis. Upon drying on the air the powder sample of **4** was found to be hygroscopic, and therefore the air-stable formula takes the form of $[Yb^{III}(2,2'\text{-bpdo})_4][Co^{III}(CN)_6] \cdot 9.5H_2O$, as revealed by the CHN elemental analysis and TG studies. The hygroscopic character does not affect the crystallinity of the sample as shown by the P-XRD studies, as the experimental powder pattern matches the calculated one obtained based on the model from a single-crystal experiment (Figure S8d). Yield (based on Yb^{3+} precursor): 75% (118 mg). CHN elem. anal. calcd. for $Yb_1Co_1C_{46}H_{51}N_{14}O_{17.5}$ (M_W = 1311.9 g·mol^{-1}): C, 42.1%; H, 3.9%; N, 15.0%. Found: C, 42.3%; H, 3.8%; N, 15.4%.

*Synthesis of **5***

Compound **5** was prepared following a procedure presented for **4**, using 55.9 mg (0.12 mmol) of potassium hexacyanidoiridate(III), $K_3[Ir(CN)_6]$ instead of $K_3[Co(CN)_6]$. The product was separated from the mother solution after ca. 2 days of crystallization using suction filtration. The composition of **5**, $[Yb^{III}(2,2'\text{-bpdo})_4][Ir^{III}(CN)_6] \cdot 6H_2O$ was determined by the SC-XRD analysis. Upon drying on the air the powder sample of **5** was found to be hygroscopic, and therefore the air-stable formula takes the form of $[Yb^{III}(2,2'\text{-bpdo})_4][Ir^{III}(CN)_6] \cdot 7H_2O$, as revealed by the CHN elemental analysis and TG studies. The hygroscopic character does not affect the crystallinity of the sample as shown by the P-XRD studies, as the experimental powder pattern matches the calculated one obtained based on the model from a single-crystal experiment (Figure S8d). Yield (based on Yb^{3+} precursor): 69% (116 mg). CHN elem. anal. calcd. for $Yb_1Ir_1C_{46}H_{46}N_{14}O_{15}$ (M_W = 1400.2 g·mol^{-1}): C, 39.5%; H, 3.3%; N, 14.0%. Found: C, 39.8%; H, 3.0%; N, 14.2%.

*Synthesis of **6***

Compound **6** was prepared following a procedure presented for **4**, using 39.5 mg (0.12 mmol) of potassium hexacyanidoferrate(III), $K_3[Fe(CN)_6]$ instead of $K_3[Co(CN)_6]$. The product was separated from the mother solution after ca. 2 days of crystallization using suction filtration. The composition of **6**, $[Yb^{III}(2,2'\text{-bpdo})_4][Fe^{III}(CN)_6] \cdot 6H_2O$ was determined by the SC-XRD analysis. Upon drying on the air the powder sample of **6** was found to be hygroscopic, and therefore the air-stable formula takes the form of $[Yb^{III}(2,2'\text{-bpdo})_4][Fe^{III}(CN)_6] \cdot 8H_2O$, as revealed by the CHN elemental analysis and TG studies. The hygroscopic character does not affect the crystallinity of the sample as shown by the P-XRD studies, as the experimental powder pattern matches the calculated one obtained based on the model from a single-crystal experiment (Figure S8d). Yield (based on Yb^{3+} precursor): 84% (129 mg). CHN elem. anal. calcd. for $Yb_1Fe_1C_{46}H_{48}N_{14}O_{16}$ (M_W = 1281.8 g·mol^{-1}): C, 43.1%; H, 3.8%; N, 15.3%. Found: C, 42.7%; H, 3.8%; N, 15.5%.

*Synthesis of **7***

Compound **7** was prepared following a procedure presented for **4**, using 39.0 mg (0.12 mmol) of potassium hexacyanidochromate(III), $K_3[Cr(CN)_6]$ instead of $K_3[Co(CN)_6]$. The product was separated from the mother solution after ca. 2 days of crystallization using suction filtration. The composition of **7**, $[Yb^{III}(2,2'\text{-bpdo})_4][Cr^{III}(CN)_6] \cdot 6H_2O$ was determined by the SC-XRD analysis. Upon drying on the air the powder sample of **7** was found to be hygroscopic, and therefore the air-stable formula takes the form of $[Yb^{III}(2,2'\text{-bpdo})_4][Fe^{III}(CN)_6] \cdot 7H_2O$, as revealed by the CHN elemental analysis and TG studies. The hygroscopic character does not affect the crystallinity of the sample as shown by P-XRD studies, as the experimental powder pattern matches the calculated one obtained based on the model from a single-crystal experiment (Figure S8d). Yield (based on Yb^{3+} precursor): 63% (97 mg). CHN elem. anal. calcd. for $Yb_1Cr_1C_{46}H_{46}N_{14}O_{15}$ (M_W = 1260.0 g·mol^{-1}): C, 43.8%; H, 3.7%; N, 15.6%. Found: C, 43.5%; H, 3.7%; N, 15.6%.

X-ray diffraction methods

Single-crystal X-ray diffraction (SC-XRD) analyses for **1** and **3–7** were performed on a Bruker D8 Quest Eco Photon50 CMOS diffractometer equipped with graphite monochromated MoKα radiation, while the single crystal of **2** was measured on a Rigaku R-AXIS RAPID diffractometer equipped with the imaging plate type detector and graphite monochromated MoKα radiation (Tables S1–S2). For the SC-XRD studies, the selected single crystals of **1**–**7** were taken directly from the respective mother solutions, covered by Apiezon® N grease, and mounted onto the Micro Mounts™ holder. The measurement for **2** was carried out at 90(2) K, and after collecting all the diffraction frames, the crystal structure of **2** was solved using a direct method within the SHELXS-97 program integrated within the Crystal Structure software.[3] The SC-XRD measurements for **1** and **3**–**7** were carried out at 100(2) K, the data reduction and cell refinement were performed using SAINT and SADABS programs, and the crystal structure solution involved an intrinsic phasing method using a SHELXT-2014/5.[4] All crystal structures were refined following a weighted full-matrix least-squares method on F^2 with SHELX-2018/3 implemented in the WinGX v2014/1 integrated system.[5, 6] All non-hydrogen atoms were refined anisotropically. The hydrogen atoms were found from the electron density map and refined using a riding model. For **1**, a considerable number of DIFIX, ISOR, and DELU restraints were applied on the atoms of strongly disordered $[Ag(CN)_2]^-$ ions and the part of 2,2'-bpdo ligands. For **2**, only very few DELU and ISOR restraints were used for the C/N atoms of the part of 2,2'-bpdo ligands. For **3**, a considerable set of DFIX, ISOR, and DELU commands were employed for the atoms of strongly disordered polycyanidocadmate(II) moieties, and some ISOR restraints for the part of water solvent molecules. For **4–7**, a few DELU and ISOR restraints were used for cyanido ligands, and some DFIX commands for the pat of water solvent molecules. All these restraints were used to ensure the convergence of the refinement procedure and maintain the proper coordination geometry. The crystallographic data were deposited in the CCDC database. Deposition numbers are 2095574–2095580 for **1**–**7**, respectively. The detailed parameters of crystal data and structure refinement are presented in Tables S1–S2, while the representative structural parameters of the obtained models are shown in Tables S3–S6. The structural figures were prepared using the Mercury 3.10.3 software. Powder X-ray diffraction (P-XRD) patterns for all materials were collected on a Bruker D8 Advance Eco diffractometer equipped with CuKα radiation (λ = 1.5418 Å). The samples of **1** and **2** were measured in capillaries under the respective mother solutions, while the rest of the compounds was air-dried and ground before the P-XRD experiment.

Physical techniques

Infrared (IR) absorption spectra were measured on the selected single-crystals using a Nicolet iN10 MX Fourier transform infrared microscope. For **1** and **2**, the crystals for the IR experiment were taken directly from the mother solutions. CHN elemental analyses were performed on the air-dried samples using an Elementar Vario Micro Cube analyzer. Thermogravimetric (TG) analyses were carried out under a nitrogen atmosphere using TG209 F1 Libra thermogravimetric analyzer. Solid-state photoluminescence spectra were gathered on a Horiba Jobin-Yvon Fluorolog-3 (FL3-211) spectrofluorimeter equipped with a 450 W Xe lamp and an InGaAs photodiode detector DSS-IGA020L cooled using liquid nitrogen. To protect **1** and **2** from the exposition to an air atmosphere, both samples were measured in the form of a mixture with Apiezon® N grease. The data analysis was carried out using FluorEssence® software. Magnetic properties were investigated using a Quantum Design MPMS XL magnetometer. For magnetic measurements, the powder samples of **1** and **2** were inserted into glass tubes under the respective mother solutions and sealed under Ar, while the remaining samples was packed in polyvinyl capsules, covered with paraffin oil and wool. All the samples were measured in plastic straws serving as a sample holder. Diamagnetic corrections from the samples and the holder were introduced.

Calculations

Continuous shape measure (CShM) analysis for the eight-coordinated Yb^{III} complexes in **1**–**7** was executed using SHAPE software, ver. 2.1.[7,8] The *ab initio* calculations of a CASSCF/RASSI/SINGLE_ANISO type were performed using OpenMolcas quantum chemistry software package based on the experimental geometry of Yb^{III} complexes in the crystal structures of **1**, **2**, **4**, and **5**. The exact details regarding those calculations, including the used basis sets, and the method of evaluating cumulative oscillator strengths are gathered in the Comment to Tables S9–S17.

RESULTS OF PRELIMINARY CHARACTERIZATION AND DETAILED STRUCTURAL STUDIES

The samples of **1**–**7** were preliminarily characterized using IR absorption spectroscopy, thermogravimetry (TG), and CHN elemental analysis (Figure S1 and Experimental Section). For the single-crystal X-ray diffraction (SC-XRD) experiments all crystals were taken directly from the mother solutions and covered by the protective grease. The results of the SC-XRD analysis for **1**–**7** were gathered in Tables S1–S6, while the crystal structures were presented in Figures 1 and S7. All the compounds crystallize in the form of supramolecular frameworks, which may be treated as ionic crystals, composed of nearly identical $[Yb^{III}(2,2'\text{-bpdo})_4]^{3+}$ complexes, which are efficiently separated within the crystal lattices by cyanido or thiocyanido metal complexes and solvent molecules of crystallization (H_2O in **1**, **3**–**7**, and MeCN for **2**).

In the crystal structure of **1**, crystallizing in the chiral $P2_12_12_1$ space group of the orthorhombic crystal system as a racemate, two independent Yb^{III} complexes can be distinguished (Figures 1a and S3–S4). Both of them reveal an identical composition and the geometry of a distorted square antiprism, given by very similar parameters of CShM analysis (Table S7). The f–block metal moieties are in **1** surrounded by anionic $[Ag^I(CN)_2]^-$ complexes, which form twisted supramolecular chains along the *c* crystallographic axis based on argentophilic interactions with the Ag^I–Ag^I distances lying in the 2.937–4.543 Å range (Figure S4). These metallophilic chains are the source of the chirality within the supramolecular framework, but effectively, the value of the Flack parameter for all checked single crystals was of ca. 0.5 (Table S1), which is presumably related to the significant structural disorder involving weakly bonded cyanido metal complexes (Figure S3). The interstitial space between both types of metal complexes is filled with 15 crystallization water molecules per two $\{YbAg_3\}$ building units, resulting in the formula of $[Yb^{III}(2,2'\text{-bpdo})_4][Ag^I(CN)_2]_3\cdot 7.5H_2O$. Most of the water molecules are located between the pyridine rings of $[Yb^{III}(2,2'\text{-bpdo})_4]^{3+}$ moieties, therefore, the H-bonding network involves mostly the contacts between the crystallization solvent itself. This may be the reason for the instability of the crystals upon drying on the air, as the sample of **1** was found to turn amorphous with the final water content of 4 molecules per $\{YbAg_3\}$ formula unit (found from CHN elemental analysis and TG analysis). To prevent this behavior, all magnetic and optical measurements were carried out on samples protected either by the mother solution or by protective grease (see Experimental Section).

All the attempts to obtain an analogous system to **1** using $[Au^I(CN)_2]^-$ were found unsuccessful, which may be related to the different strengths of the respective metallophilic interactions. However, by exchanging the cyanido complex to thiocyanido one, the crystals of **2** were obtained. The resulting system was found to crystallize in the *C*2/c space group of the monoclinic crystal system (Table S1). In this case, there is only one type of crystallographically independent Yb^{III} complexes, which, similarly to their analogs of **1**, reveal the geometry of a distorted square antiprism, according to the CShM analysis (Table S7). Present counter-ions in the form of $[Au^I(SCN)_2]^-$ complexes form local trinuclear units based on Au^I–Au^I contacts of ca. 3 Å (Figure 1b and Table S5). Both thiocyanide groups, as well as MeCN molecules of crystallization weakly interact with pyridine rings forming π–contacts. The determined formula of $[Yb^{III}(2,2'\text{-bpdo})_4][Au^I(SCN)_2]_3\cdot 2MeCN$ is preserved in the mother solution or when crystals of **2** are covered by the protective grease, but the exposition to an air atmosphere leads to the removal of one MeCN molecule per $\{YbAu_3\}$ unit (found from CHN and TG analyses).

The use of $K_2[Cd^{II}(CN)_4]$ salt resulted in the formation of **3**, which crystallizes in the *C*2/c space group of the monoclinic crystal system (Table S1). In **3**, two independent centers of Yb^{III} are present in the form of $[Yb^{III}(2,2'\text{-bpdo})_4]^{3+}$ complexes, and they both adopt the geometry of a distorted square antiprism (Table S7). Despite the lack of metallophilic interactions for tetrahedral Cd^{II} centers, in the crystal structure of **3**, two types of cyanido complexes were found (Figures 1c and S6). Apart from the tetrahedral ones used in the original synthesis, $[Cd^{II}(CN)_4]^{2-}$, the final crystal structure contains also $[Cd^{II}_2(CN)_7]^{3-}$ ions that appeared due to the decomposition of the cyanido precursor in the mother solution during the crystallization process. The latter complexes are in partial structural disorder related to the Cd^{II}–CN–Cd^{II} molecular bridge. The complexity of **3** results in the final formula of $[Yb^{III}(2,2'\text{-bpdo})_4]_9[Cd^{II}(CN)_4]_6\,[Cd^{II}_2(CN)_7]_5\cdot 30H_2O$, and the water of crystallization is partially hydrogen-bonded to terminal cyanido ligands. When exposed to an air atmosphere, the crystals of **3** preserve their crystal structure as proven by the P-XRD experiment (Figure S8) but they are hygroscopic and the resulting air-stable phase contains 44 water molecules per formula unit (as indicated by the CHN elemental analysis and TG studies).

The application of hexacyanidometallate(III) ions, $[M^{III}(CN)_6]^{3-}$ (M^{III} = Co, Ir, Fe, Cr) leads to the spontaneous formation of isostructural **4**, **5**, **6**, and **7**, respectively, which crystallize in the *F*ddd space group of the orthorhombic crystal system (Table S2). Compared to **1**–**3**, the adopted geometry for Yb^{III} complexes recede from the ideal square antiprism and is somehow the mixture of the latter and the geometry of a dodecahedron, which is visible in similar values of CShM parameters for both polyhedrons (Table S7). As typical for $[M^{III}(CN)_6]^{3-}$ ions in reported crystal structures, they reveal a nearly ideal octahedral geometry with only slightly modulated metal–cyanide distance between the analogs (Table S6). Single-crystal data show that in all four compounds, four crystallization water molecules are present per $[Yb^{III}(2,2'\text{-bpdo})_4][M^{III}(CN)_6]$ formula. They interact through

hydrogen bonds with each other and with terminal ends of cyanido ligands, forming the chain-like structure within channels of a small radius along the *a* crystallographic axis. Despite being structural analogs, **4**–**7** reveal different air-stable content of water, as all of them were found to be hygroscopic. Based on the CHN and TG analyses 9.5, 7, 8, and 7 water molecules per formula unit were found for **4**, **5**, **6**, and **7**, respectively. This may be roughly correlated with the size of cyanido metal complex, which is similar for **5** and **7**, smaller for **6**, while the smallest metal-cyanide distances are present for Co^{III}–based **4** (Table S6).

In the whole presented series, $[Yb^{III}(2,2'\text{-bpdo})_4]^{3+}$ moieties are well separated within the crystal lattice, both by d–block metal complexes, as well as solvent molecules of crystallization. The shortest Yb^{III}–Yb^{III} distances can be found in **1** of 9.384 and 9.563 Å, while the longest is present in **4**–**7**, being above 10.1 Å (Tables S3–S4).

The phase purity of all powder samples and the validity of the proposed structural models for **1**–**7** were checked using the powder X-ray diffraction method (Figure S8). These experiments also confirm the stability of **3**–**7** upon drying on the air, despite variable water content, when compared with the SC-XRD data.

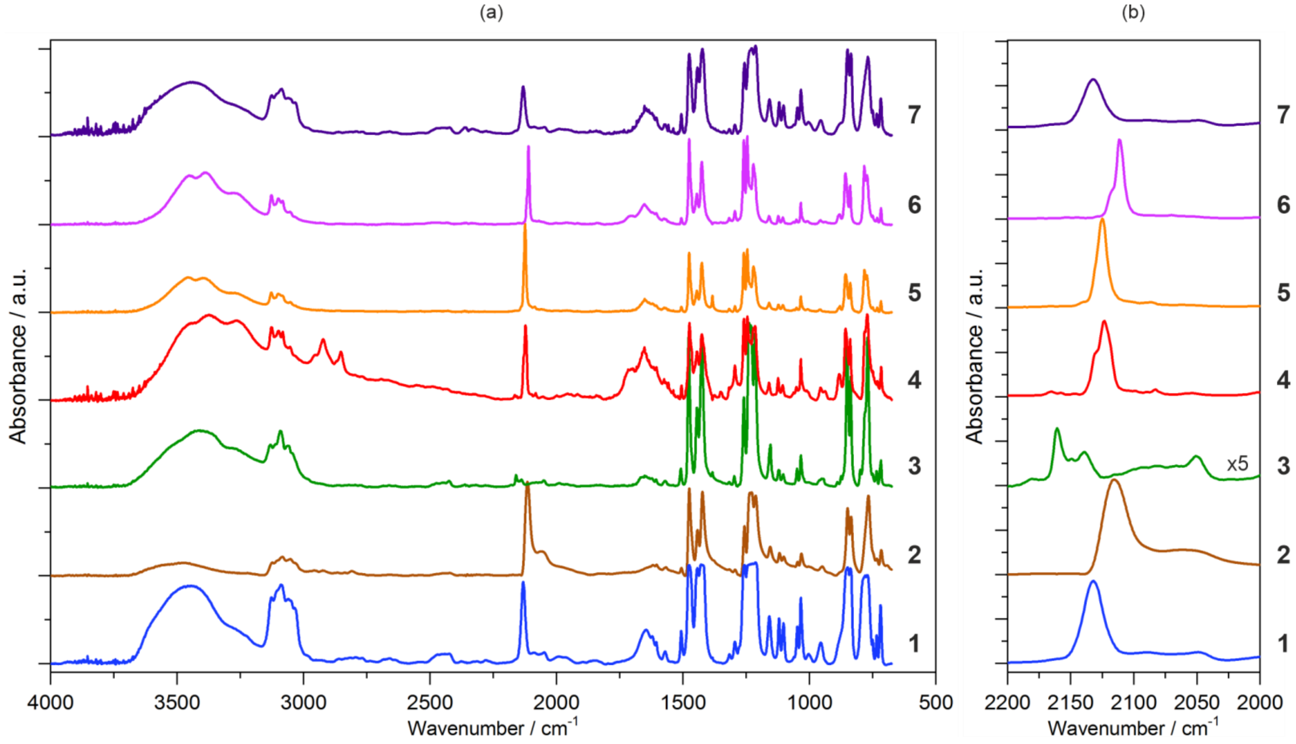

Figure S1. Infrared (IR) absorption spectra of **1**–**7** gathered in the 675–4000 cm^{-1} range (*a*) and the enlargement of the wavenumber region of 2000–2200 cm^{-1} representing the stretching vibrations of cyanido ligands (*b*).

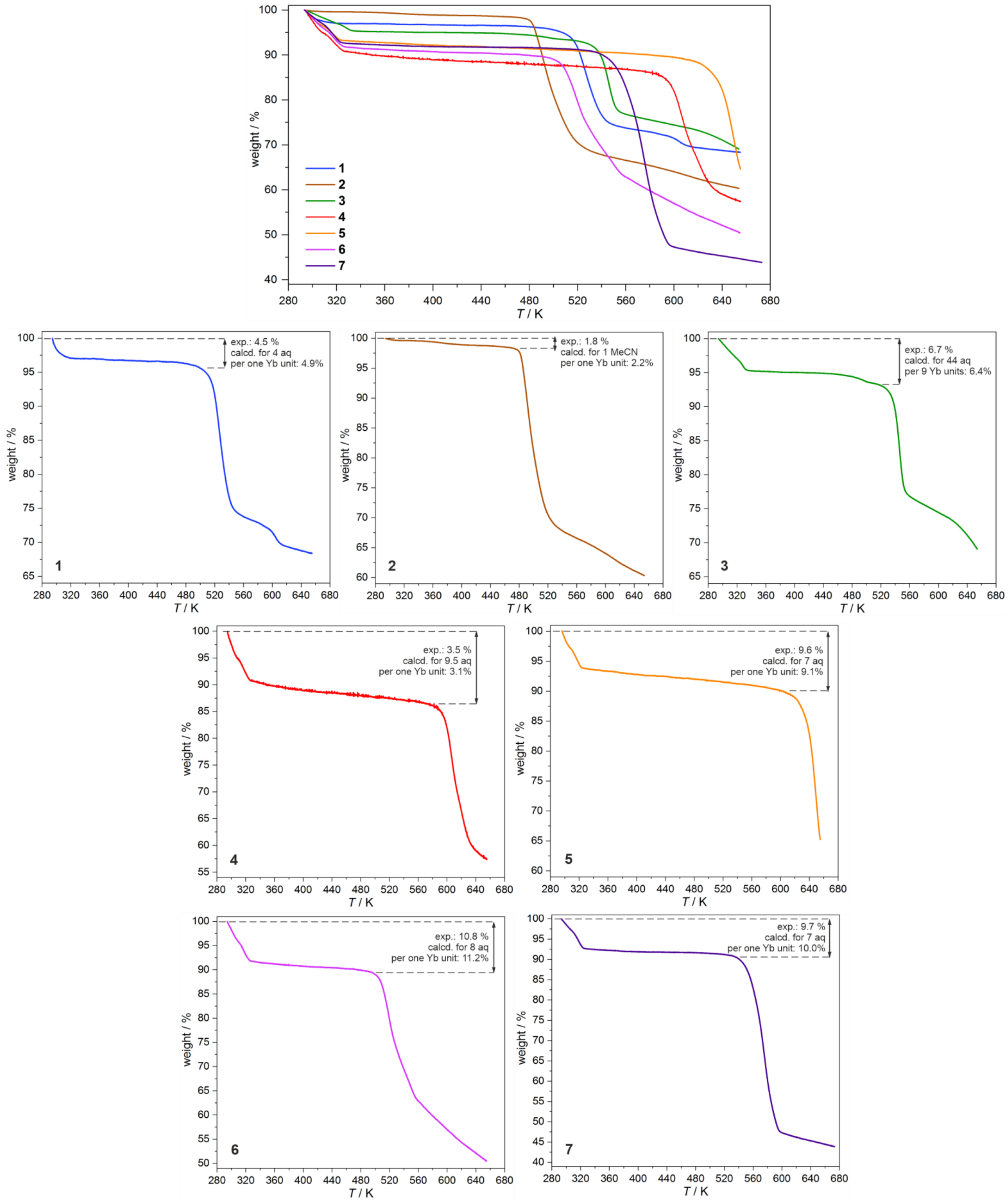

Figure S2. Thermogravimetric curves of **1**–**7** collected under nitrogen atmosphere with the heating rate of 1 K per minute. The upper graph contains the comparison of TG curves for all compounds while the other graphs show separately TG curves for each compound. The steps related to the loss of water molecules (**1**, **3**–**7**) or MeCN solvent molecules (**2**) were depicted on the graphs, and they are accompanied by the comparison with the calculated values for the presented amount of solvent molecules per Yb units (see Experimental section for details).

Table S1. Crystal data and structure refinement for **1–3**.

compound	**1**	**2**	**3**
formula	$Yb_2Ag_6C_{92}H_{64}N_{28}O_{31}$	$Yb_1Au_3C_{50}H_{38}N_{16}O_8S_6$	$Yb_3Cd_{5.33}C_{139.67}H_{96}N_{43.67}O_{34}$
formula weight / g·mol^{-1}	3051.01	1947.26	4048.51
T / K	100(2)	90(2)	100(2)
λ / Å	0.71073 (Mo Kα)		
crystal system	orthorhombic	monoclinic	
space group	$P2_12_12_1$ (no. 19)	$C2$/c (no. 15)	
a / Å	13.117(3)	22.961(3)	53.198(4)
b / Å	24.758(4)	14.1072(16)	12.3749(9)
c / Å	37.628(7)	18.6968(19)	24.9475(18)
α, γ / deg	90	90	90
β / deg		103.936(7)	90.362(3)
V / Å^3	12220(4)	5877.9(11)	16423(2)
Z	4		
ρ_{calc} / g·cm^{-3}	1.658	2.2	1.637
μ / cm^{-1}	2.534	9.321	2.449
F(000)	5928	3668	7911
crystal type	colorless block		
crystal size / mm × mm × mm	0.28 × 0.22 × 0.10	0.13 × 0.10 × 0.10	0.13 × 0.13 × 0.11
θ range / deg	2.695–26.531	3.063–27.478	2.348–25.349
limiting indices	$-16 < h < 16$<br>$-30 < k < 31$<br>$-46 < l < 47$	$-29 < h < 29$<br>$-18 < k < 15$<br>$-24 < l < 24$	$-64 < h < 64$<br>$-14 < k < 14$<br>$-30 < l < 30$
collected reflections	74569	28238	76626
unique reflections	25126	6736	15009
R_{int}	0.097	0.0604	0.0451
completeness / %	99.7	99.8	99.9
data/restraints/parameters	25126/327/1589	6736/38/381	15009/80/1095
GOF on F^2	1.089	1.388	1.033
final R indices	R_1 = 0.095 [$I \geq 2\sigma(I)$]<br>wR_2 = 0.254 (all data)	R_1 = 0.067 [$I \geq 2\sigma(I)$]<br>wR_2 = 0.0795 (all data)	R_1 = 0.0471 [$I \geq 2\sigma(I)$]<br>wR_2 = 0.142 (all data)
largest diff. peak/hole / e·Å^{-3}	4.346/–2.430	2.801/–4.179	3.166/–1.310
Flack x-parameter	0.495(5)	-	-

Table S2. Crystal data and structure refinement for **4–7**.

compound	**4**	**5**	**6**	**7**
formula	$Yb_1Co_1C_{46}H_{44}N_{14}O_{14}$	$Yb_1Ir_1C_{46}H_{44}N_{14}O_{14}$	$Yb_1Fe_1C_{46}H_{44}N_{14}O_{14}$	$Yb_1Cr_1C_{46}H_{32}N_{14}O_{14}$
formula weight / g·mol^{-1}	1248.92	1382.19	1245.84	1229.89
T / K	100(2)			
λ / Å	0.71073 (Mo Kα)			
crystal system	orthorhombic			
space group	*F*ddd (no. 70)			
a / Å	13.8229(10)	13.8789(14)	13.8260(7)	13.9366(11)
b / Å	25.6413(19)	25.716(3)	25.7260(12)	25.744(2)
c / Å	28.239(2)	28.221(3)	28.2630(13)	28.199(3)
α, β, γ / deg	90			
V / Å^3	10008.9(13)	10072.2(17)	10052.8(8)	10117.5(15)
Z	8			
ρ_{calc} / g·cm^{-3}	1.658	1.823	1.646	1.615
μ / cm^{-1}	2.27	4.566	2.218	2.132
F(000)	5016	5416	5008	4896
crystal type	colorless needle		yellow needle	
crystal size / mm × mm × mm	0.39 × 0.08 × 0.07	0.43 × 0.05 × 0.05	0.45 × 0.06 × 0.05	0.15 × 0.06 × 0.04
θ range / deg	2.736– 27.101	2.733– 27.101	2.733–27.103	2.731–27.101
limiting indices	$-17 < h < 17$<br>$-32 < k < 32$<br>$-36 < l < 36$	$-17 < h < 17$<br>$-32 < k < 32$<br>$-36 < l < 25$	$-17 < h < 17$<br>$-32 < k < 32$<br>$-36 < l < 36$	$-17 < h < 17$<br>$-32 < k < 32$<br>$-35 < l < 36$
collected reflections	22298	14734	26440	17013
unique reflections	2767	2789	2785	2802
R_{int}	0.0521	0.0402	0.0477	0.0863
completeness / %	99.9			
data/restraints/parameters	2767/35/192	2789/39/192	2785/35/192	2802/39/179
GOF on F^2	1.063	1.134	1.071	1.036
final *R* indices	R_1 = 0.0235<br>[$I \geq 2\sigma(I)$]<br>wR_2 = 0.0506<br>(all data)	R_1 = 0.0703<br>[$I \geq 2\sigma(I)$]<br>wR_2 = 0.1303<br>(all data)	R_1 = 0.0248<br>[$I \geq 2\sigma(I)$]<br>wR_2 = 0.0516<br>(all data)	R_1 = 0.0529<br>[$I \geq 2\sigma(I)$]<br>wR_2 = 0.1212<br>(all data)
largest diff. peak/hole / e·Å^{-3}	0.676/–0.341	6.200/–6.619	0.903/–0.94	2.690/–2.879

Table S3. Detailed structure parameters of ytterbium(III) complexes in **1**–**3**.

parameter (see Fig. 1)	**1**, Yb1	**1**, Yb2	**2**, Yb1	**3**, Yb1	**3**, Yb2
Yb–O distances / Å	2.281(14) (O1)	2.279(16) (O9)	2.317(5) (O1)	2.274(4) (O1)	2.265(5) (O5)
	2.378(15) (O2)	2.380(16) (O10)			2.335(5) (O6)
	2.359(13) (O3)	2.257(18) (O11)	2.258(5) (O2)	2.337(4) (O2)	2.354(5) (O7)
	2.238(13) (O4)	2.37(2) (O12)			2.263(5) (O8)
	2.263(13) (O5)	2.350(16) (O13)	2.387(5) (O3)	2.346(4) (O3)	2.363(5) (O9)
	2.417(14) (O6)	2.269(18) (O14)			2.269(5) (O10)
	2.350(14) (O7)	2.372(14) (O15)	2.292(5) (O4)	2.290(4) (O4)	2.373(6) (O11)
	2.286(12) (O8)	2.158(19) (O16)			2.262(5) (O12)
representative O–Yb–O angles / deg	71.7(5) (O1–Yb1–O2)	69.3(9) (O11–Yb2–O12)	72.84(18) (O1–Yb1–O4)	72.38(14) (O2–Yb1–O1)	71.9(2) (O12–Yb2–O11)
	70.1(5) (O2–Yb1–O4)	72.7(8) (O12–Yb2–O14)	72.47(18) (O4–Yb1–O3)	71.97(14) (O1–Yb1–O3)	72.6(2) (O11–Yb2–O5)
	72.8(5) (O4–Yb1–O3)	73.8(6) (O14–Yb2–O13)	70.85(18) (O3–Yb1–O2)	72.03(14) (O3–Yb1–O4)	73.4(2) (O5–Yb2–O6)
	73.1(5) (O3–Yb1–O1)	71.4(6) (O13–Yb2–O11)	73.29(19) (O2–Yb1–O1)	72.04(13) (O4–Yb1–O2)	72.56(19) (O6–Yb2–O12)
	72.6(5) (O5–Yb1–O6)	72.4(6) (O9–Yb2–O10)	74.05(17) (O1–Yb1–O3’)	82.0(2) (O1–Yb1–O1’)	71.34(16) (O9–Yb2–O10)
	70.5(5) (O6–Yb1–O8)	73.2(6) (O10–Yb2–O16)	79.28(18) (O1–Yb1–O4’)	81.81(14) (O1–Yb1–O3’)	71.99(17) (O10–Yb2–O7)
	72.1(5) (O7–Yb1–O8)	71.6(6) (O16–Yb2–O15)	81.06(18) (O2–Yb1–O3’)	74.63(14) (O3–Yb1–O2’)	72.34(19) (O7–Yb2–O8)
	74.5(5) (O5–Yb1–O7)	72.3(6) (O15–Yb2–O9)	83.2(3) (O2–Yb1–O2’)	80.71(13) (O4–Yb1–O2’)	72.04(17) (O8–Yb2–O9)
	82.5(5) (O1–Yb1–O5)	71.4(8) (O10–Yb2–O12)	83.0(3) (O4–Yb1–O4’)	80.96(19) (O4–Yb1–O4’)	79.06(17) (O12–Yb2–O9)
	71.2(5) (O2–Yb1–O7)	86.4(8) (O11–Yb2–O16)	↑ O(1–4) and O(1–4)’ are related to two distorted squares representing Yb1 complexes of a square antiprism geometry (Fig. 1b)	↑ O(1–4) and O(1–4)’ are related to two distorted squares representing Yb1 complexes of a square antiprism geometry (Fig. 1c)	78.7(2) (O6–Yb2–O8)
	83.0(5) (O4–Yb1–O8)	70.8(5) (O13–Yb2–O15)			79.07(12) (O5–Yb2–O7)
	72.0(5) (O3–Yb1–O6)	80.3(7) (O14–Yb2–O9)			78.25(18) (O11–Yb2–O10)
shortest Yb–Yb distances / Å	9.384<br>9.563<br>(Yb1–Yb2 along *c* axis)		9.859<br>(Yb1–Yb1 along *c* axis)	9.729<br>(Yb1–Yb2 along [101] direction)<br>10.085<br>(Yb2–Yb2 along [101] direction)	

Table S4. Detailed structure parameters of ytterbium(III) complexes in **4–7**.

parameter (see Fig. 1d)	**4**, Yb1	**5**, Yb1	**6**, Yb1	**7**, Yb1
Yb–O distances / Å	2.3717(16) (O1)	2.372(5) (O1)	2.3722(17) (O1)	2.369(4) (O1)
	2.2431(16) (O2)	2.244(6) (O2)	2.2479(17) (O2)	2.249(4) (O2)
representative O–Yb–O angles / deg	71.98(6)<br>72.51(6)<br>(O1–Yb1–O2)	71.9(2)<br>72.6(2)<br>(O1–Yb1–O2)	71.93(6)<br>72.51(6)<br>(O1–Yb1–O2)	72.14(14)<br>72.55(14)<br>(O1–Yb1–O2)
	69.60(8)<br>(O1–Yb1–O1’)	69.9(3)<br>(O1–Yb1–O1’)	69.65(8)<br>(O1–Yb1–O1’)	69.72(19)<br>(O1–Yb1–O1’)
	88.29(9)<br>(O2–Yb1–O2’)	88.5(3)<br>(O2–Yb1–O2’)	88.27(9)<br>(O2–Yb1–O2’)	87.9(2)<br>(O2–Yb1–O2’)
	81.50(6)<br>(O1–Yb1–O2’)<br>(O2–Yb1–O1’)	81.3(2)<br>(O1–Yb1–O2’)<br>(O2–Yb1–O1’)	81.56(6)<br>(O1–Yb1–O2’)<br>(O2–Yb1–O1’)	81.24(14)<br>(O1–Yb1–O2’)<br>(O2–Yb1–O1’)
	↑ O(1–2) and O(1–2)’ are related to two different distorted squares representing Yb1 complexes of a square antiprism geometry (Fig. 1d)			
	122.93(8)<br>(O1–Yb1–O1)	122.9(3)<br>(O1–Yb1–O1)	122.83(8)<br>(O1–Yb1–O1)	122.94(19)<br>(O1–Yb1–O1)
	100.65(9)<br>(O2–Yb1–O2)	100.7(3)<br>(O2–Yb1–O2)	100.67(9)<br>(O2–Yb1–O2)	101.2(2)<br>(O2–Yb1–O2)
shortest Yb–Yb distances / Å	10.143	10.156	10.161	10.162
	(shortest Yb1–Yb1 distance is observed both within the columnar arrangement along [101] direction as well as between the columns, Fig. 1d)			

Table S5. Detailed structure parameters of cyanido metal complexes in **1**–**3**.

**1**, $[Ag^{I}(CN)_2]^-$ complexes (distances, Å, and angles, deg)					
Ag1–C81	2.08(4)	Ag4–C87	1.79(3), 2.25(3)	Ag7–C93	1.88(9), 1.96(3), 2.18(3)
Ag1–C82	2.03(4)	Ag4–C88	1.87(3), 2.23(4)	Ag7–C94	1.89(3), 1.95(9), 2.04(3)
C81–Ag1–C82	171.1(12)	C87–Ag4–C88	155(3), 178(2)	C93–Ag7–C94	120(6), 157(5), 174(3)
Ag1–C81–N17	174(3)	Ag4–C87–N23	172(4), 176(5)	Ag7–C93–N29	147(10), 153(10), 167(8)
Ag1–C82–N18	177(3)	Ag4–C88–N24	165(5), 165(6)	Ag7–C94–N30	127(9), 129(5), 151(10)
Ag2–C83	1.953(14), 2.103(14)	Ag5–C89	1.94(3)		
Ag2–C84	2.101(14), 2.101(14)	Ag5–C90	2.00(3)	Ag4---Ag2	3.229, 3.398
C83–Ag2–C84	173(3), 175(3)	C89–Ag5–C90	168(3)		
Ag2–C83–N19	155(6), 169(4)	Ag5–C89–N25	163(7)	Ag2---Ag1	3.028, 3.040
Ag2–C84–N20	165(8), 166(7)	Ag5–C90–N26	145(5)	Ag1---Ag3	3.111
Ag3–C85	2.03(5)	Ag6–C91	2.07(2), 2.15(3)	Ag3---Ag6	3.176, 3.182
Ag3–C86	2.07(4)	Ag6–C92	1.94(3), 2.167(17)	Ag6---Ag7	2.937, 3.130
C85–Ag3–C86	161.8(13)	C91–Ag6–C92	163(3), 173(2)		
Ag3–C85–N21	173(3)	Ag6–C91–N27	149(4), 168(8)	Ag7---Ag5	3.321, 4.543
Ag3–C86–N22	168(4)	Ag6–C92–N28	152(6), 175(4)	(Ag5)N25---Ag4	3.267, 3.730
**2**, $[Au^{I}(SCN)_2]^-$ complexes (distances, Å, and angles, deg)					
Au1–S1	2.303(2)	Au1–S1–C22	100.6(3)	Au2–S9	2.311(2)
Au1–S2	2.290(2)	S1–C22–N6	177.1(9)	S9–Au2–S9	176.27(12)
S1–Au1–S2	176.28(9)	Au1–S2–C21	102.9(3)	Au2–S9–C23	101.1(3)
Au1---Au2	3.023	S2–C21–N5	177.7(9)	S9–C23–N7	176.7(8)
**3**, $[Cd^{II}_2(CN)_7]^{3-}$ and $[Cd^{II}(CN)_4]^{2-}$ complexes (distances, Å, and angles, deg)					
Cd1–C61	2.242(9)	Cd2–C68/N20	2.243(6)	Cd3–C72/N24	2.11(2)
Cd1–C62	2.211(9)	Cd2–C65	2.237(8)	Cd3–C69	2.155(16)
Cd1–C63	2.216(9)	Cd2–C66	2.204(6)	Cd3–C70	2.15(2)
Cd1–C64	2.187(10)	Cd2–C67	2.203(7)	Cd3–C71	2.282(16)
C61–Cd1–C62	104.1(3)	C68–Cd2–C65	102.3(2)	C72–Cd3–C69	98.1(12)
C61–Cd1–C63	110.1(3)	C68–Cd2–C66	114.8(2)	C72–Cd3–C70	111.9(8)
C61–Cd1–C64	109.2(4)	C68–Cd2–C67	108.3(2)	C72–Cd3-C71	106.3(10)
C62–Cd1–C63	108.0(3)	C67–Cd2–C66	114.6(2)	C69–Cd3–C70	114.7(12)
C62–Cd1–C64	116.7(4)	C67–Cd2–C65	110.6(3)	C69–Cd3–C71	111.4(13)
C63–Cd1–C64	108.7(3)	C66–Cd2–C65	105.4(2)	C70–Cd3–C71	113.2(10)
Cd1–C61–N13	176.1(8)	Cd2–C65–N17	175.7(7)	Cd3–C72–N24	173(3)
Cd1–C62–N14	175.6(9)	Cd2–C66–N18	170.1(5)	Cd3–C70–N22	172(2)
Cd1–C63–N15	177.3(7)	Cd2–C67–N19	174.8(6)	Cd3–C71–N23	164(3)
Cd1–C64–N16	176.1(15)	Cd2–C68–N20	171.3(2)	Cd3–C69–N21	175(4)
Cd1–Cd2	8.858	Cd2–Cd2'	5.595	Cd3A–C72A/N24A	2.253(19)
Cd1–Cd3	6.715	Cd3–Cd3'	5.542	Cd3A–C70A	2.19(2)

Table S6. Detailed structure parameters of cyanido metal complexes in **4–7**.

$[M^{III}(CN)_6]^{3-}$ complexes	**4** (M = Co)	**5** (M = Ir)	**6** (M = Fe)	**7** (M = Cr)
M1–C1 / Å	1.895(4)	1.99(2)	1.943(4)	2.052(15)
M1–C2 / Å	1.894(3)	2.063(15)	1.937(4)	2.064(11)
C1–M1–C1 / deg	180	180	180	180
C2–M1–C2 / deg	175.65(19)<br>86.99(19)<br>93.18(19)	175.1(9)<br>85.8(8)<br>94.4(7)	175.4(2)<br>85.9(2)<br>94.3(2)	175.5(6)<br>86.8(5)<br>93.4(5)
C1–M1–C2 / deg	87.82(10)<br>92.18(10)	87.6(4)<br>92.4(4)	87.68(11)<br>92.32(11)	87.7(3)<br>92.3(3)
M1–C1–N1 / deg	180	180	180	180
M1–C2–N2 / deg	176.3(3)	175.8(13)	176.1(3)	172.3(9)
M1–M1' / Å	10.143	10.156	10.161	10.162

Table S7. Results of Continuous Shape Measure Analysis (CShM) for $[Yb^{III}(2,2'\text{-bpdo})_4]^{3+}$ complexes in **1**–**7**.

compound	complex	CShM parameters[a]			determined geometry
		SAPR–8	TDD–8	BTPR–8	
**1**	Yb1	**0.877**	2.209	2.069	SAPR-8
	Yb2	**0.973**	2.260	2.102	SAPR-8
**2**	Yb1	**0.578**	1.692	1.932	SAPR-8
**3**	Yb1	**0.482**	2.181	2.096	SAPR-8
	Yb2	**0.706**	1.151	1.864	SAPR-8
**4**	Yb1	**1.379**	**1.316**	1.964	SAPR-8/TDD-8
**5**	Yb1	**1.302**	**1.296**	1.925	SAPR-8/TDD-8
**6**	Yb1	**1.369**	**1.323**	1.966	SAPR-8/TDD-8
**7**	Yb1	**1.356**	**1.259**	1.950	SAPR-8/TDD-8

[a]CShM parameters:
CShM SAPR-8 is the parameter representing how close is the experimental geometry of the complex to the ideal square antiprism (D_{4d} symmetry). CShM TDD-8 is the parameter representing how close is the experimental geometry of the complex to the ideal triangular dodecahedron (D_{2d} symmetry). CShM BTPR-8 is the parameter representing how close is the experimental geometry of the complex to the ideal bicapped trigonal prism (C_{2v} geometry). The CShM parameter equals zero for the ideal geometry and increases upon the increasing distortion from the ideal polyhedron.[7,8]

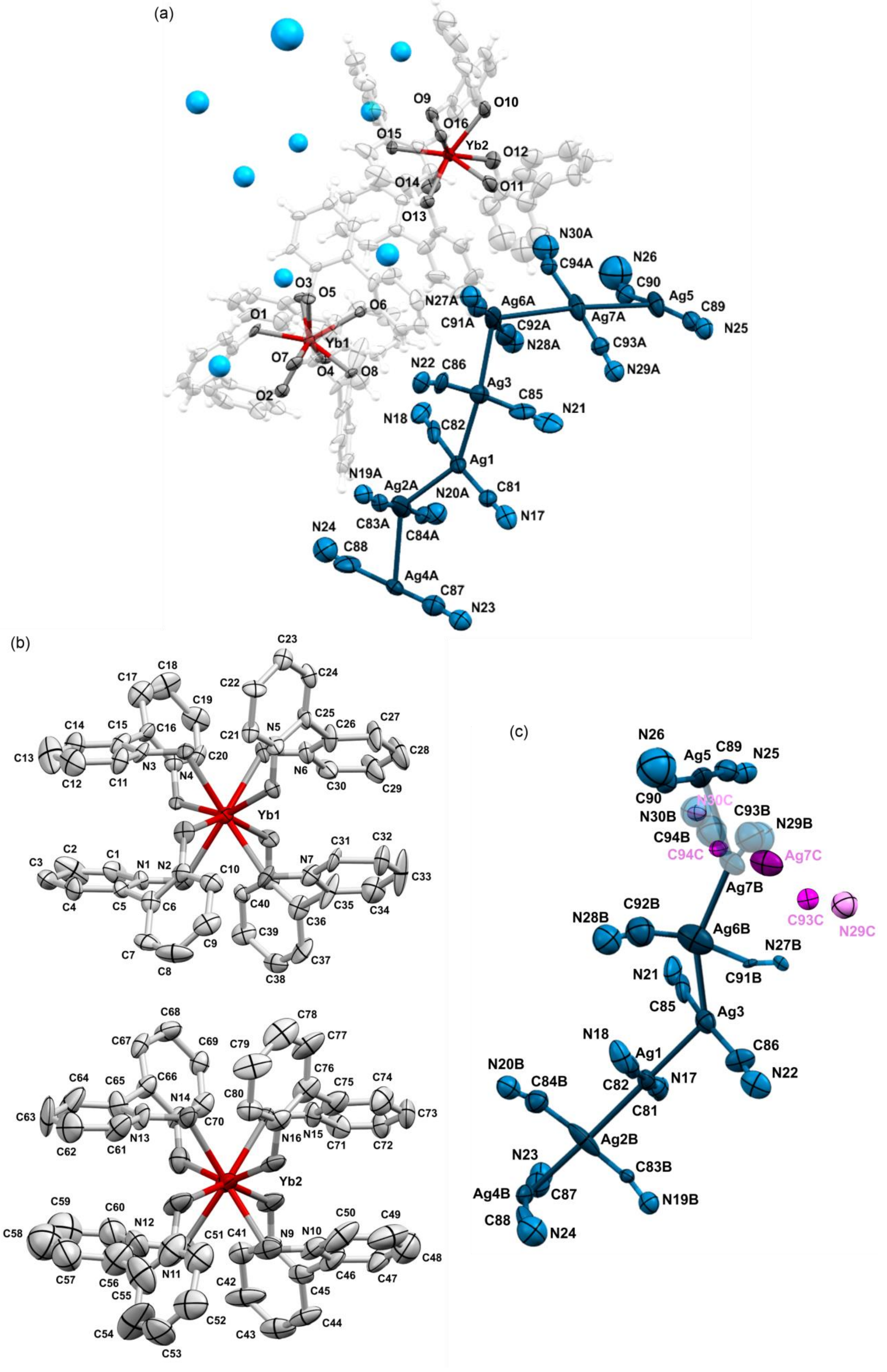

Figure S3. The representative structural fragments of **1**: the asymmetric unit incorporating the A positions of the part of $[Ag(CN)_2]^-$ ions existing in more than one crystallographic positions (*a*), the detailed insight into the Yb1 and Yb2 complexes belonging to the asymmetric unit (*b*), and the graphical presentation of the alternative B and C positions of $[Ag(CN)_2]^-$ ions (*c*). All atoms were shown at the 50% probability level. The related bond lengths and angles are collected in Tables S3 and S5. Light blue balls in (*a*) represent the O atoms of crystallization water.

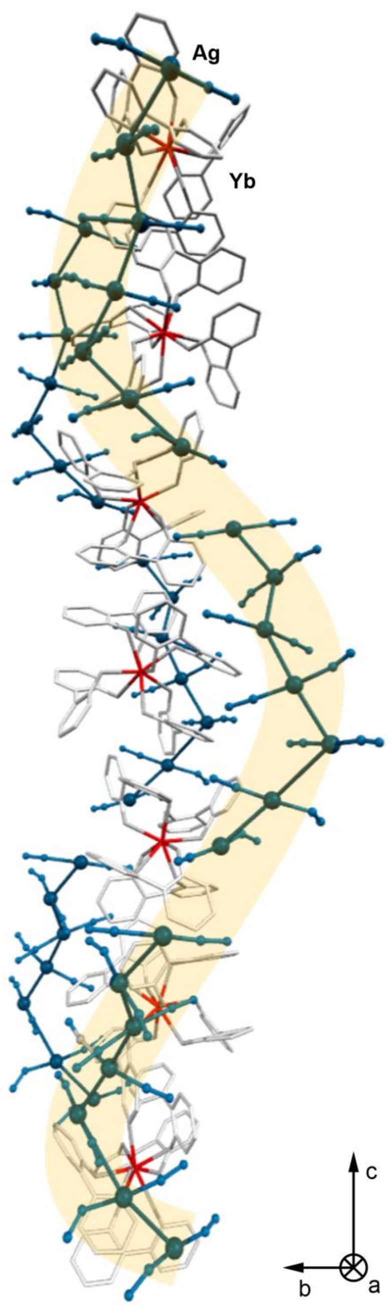

Figure S4. The detailed structural view of the helical supramolecular arrangement of metallophilic $[Ag^{I}(CN)_2]^{-}$-based aggregates around the $[Yb^{III}(2,2'\text{-bpdo})_4]^{3+}$ complexes in the crystal structure of **1**.

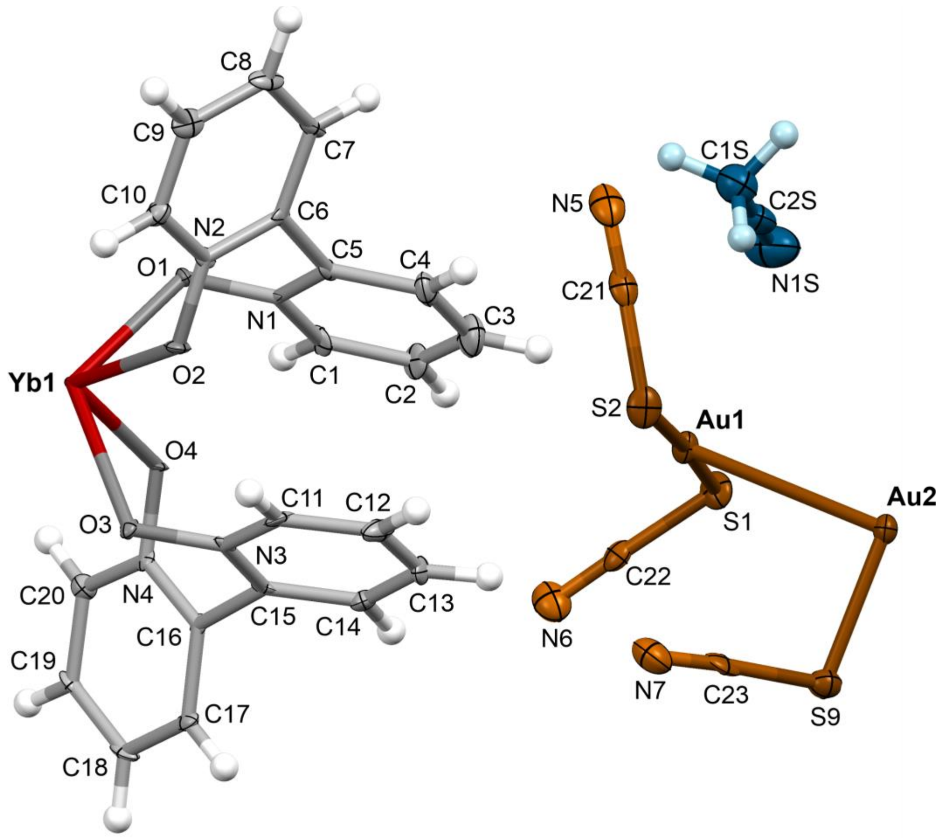

Figure S5. The asymmetric unit of **2** presented with the atoms labeling scheme. All atoms were shown at the 50% probability level. The related bond lengths and angles are collected in Tables S3 and S5.

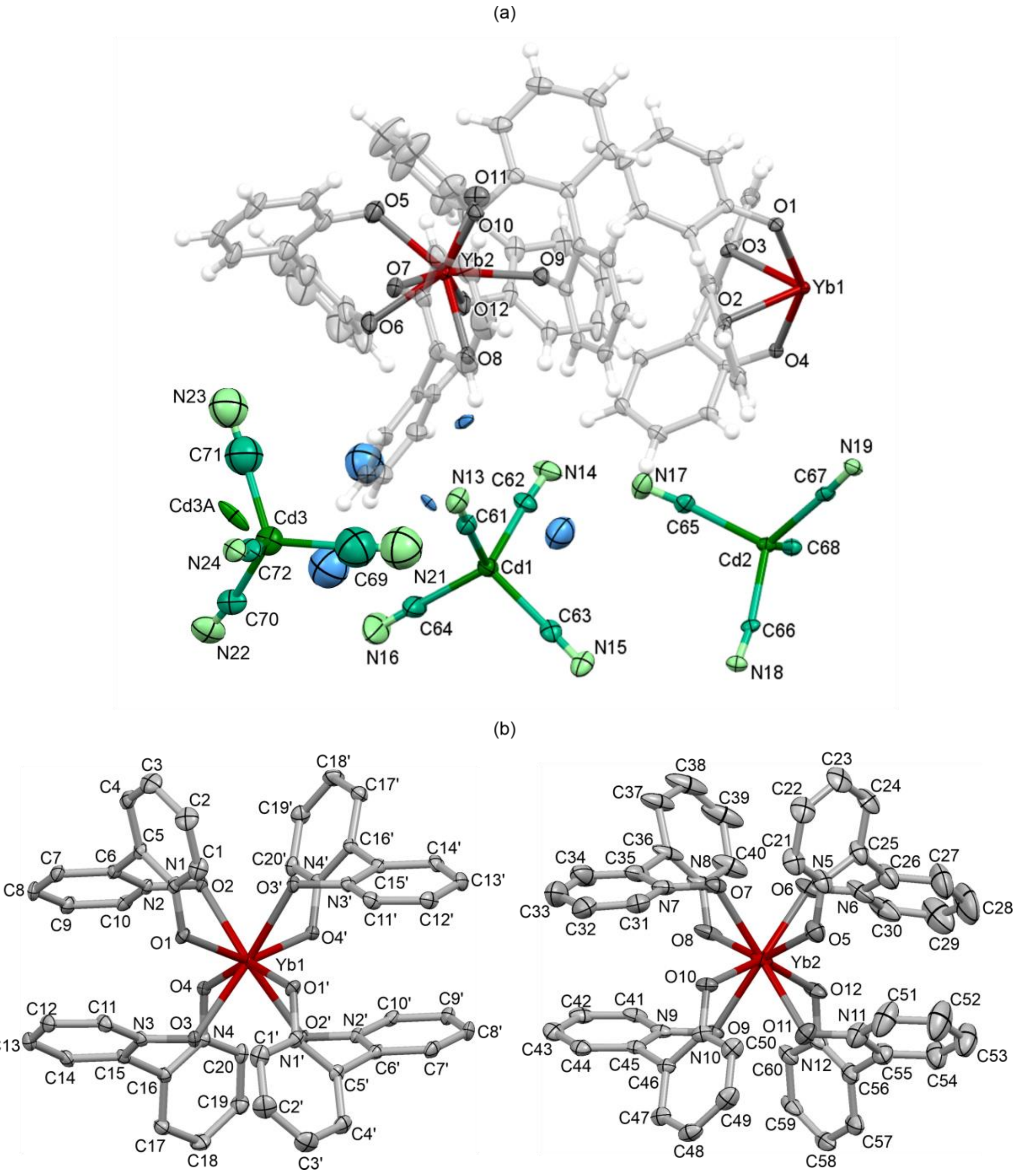

Figure S6. The representative structural fragments of **3**: the asymmetric unit with the atoms labeling scheme excluding atoms of 2,2’-bpdo rings and crystallization water molecules (*a*) and the detailed insight into the Yb1 and Yb2 complexes belonging to the asymmetric unit with the related atoms labeling scheme (*b*). All atoms were shown at the 30% probability level. The related bond lengths and angles are collected in Tables S3 and S5. Light blue balls in (*a*) represent the O atoms of crystallization water.

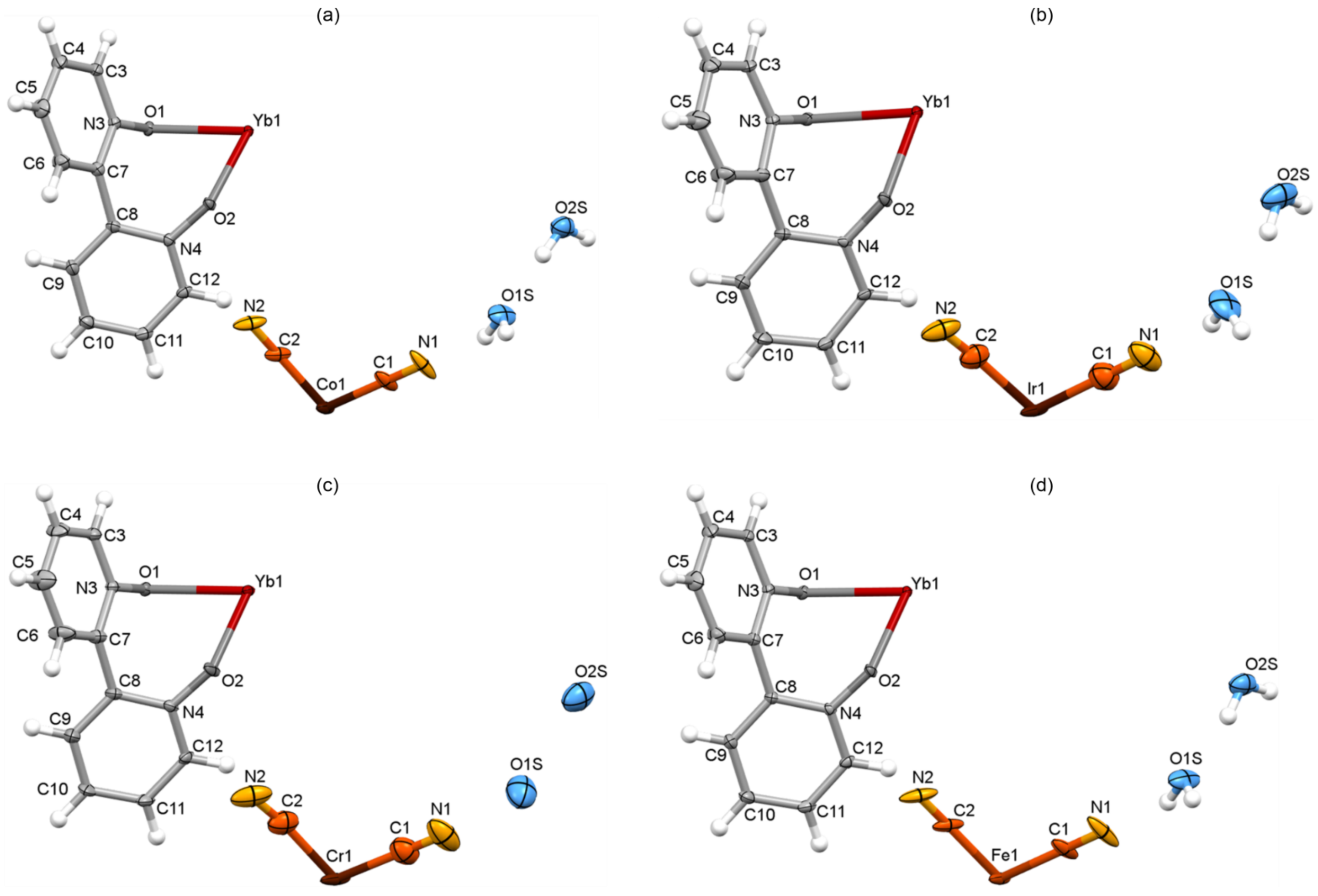

Figure S7. The asymmetric units of **4** (*a*), **5** (*b*), **6** (*c*), and **7** (*d*) presented with the respective atoms labeling schemes. All atoms were shown at the 50% probability level. The related bond lengths and angles are collected in Tables S4 and S6.

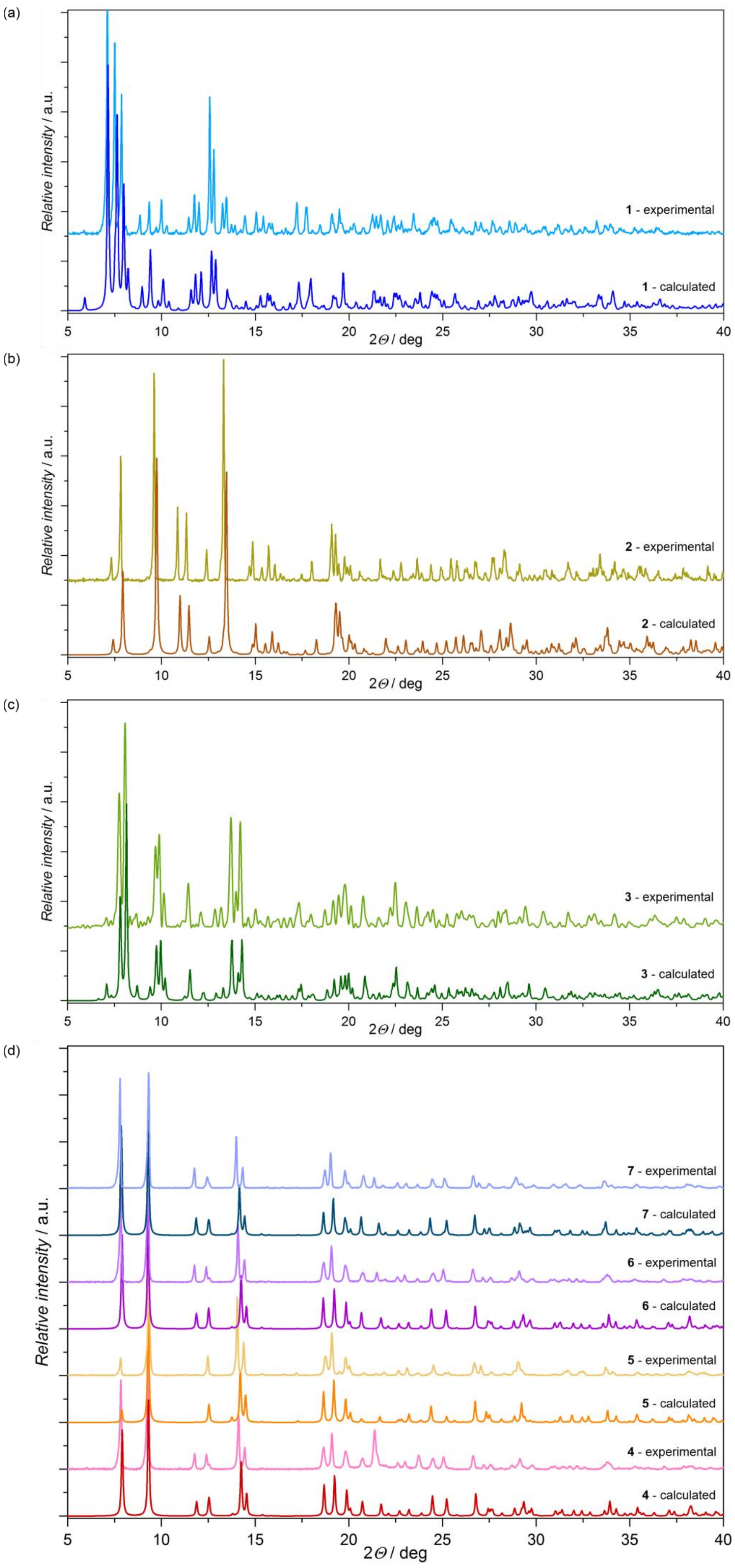

Figure S8. Powder X-ray diffraction (PXRD) patterns for **1** (*a*), **2** (*b*), **3** (*c*), and **4–7** (*d*). Calculated patterns were obtained based on the respective structural models obtained by a single-crystal X-ray diffraction method.

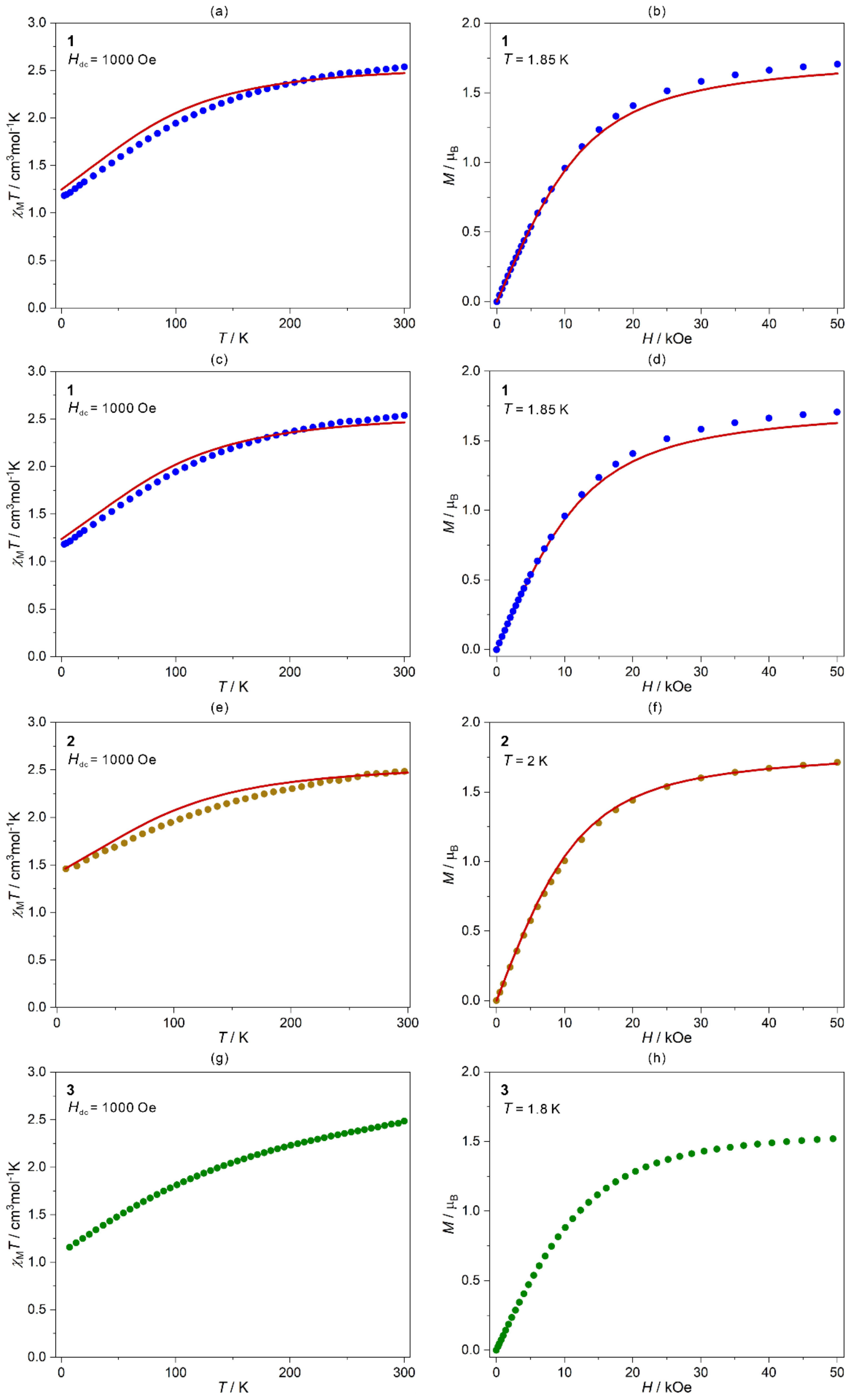

Figure S9. Direct-current (*dc*) magnetic properties of **1** (a-d), **2** (e, f), and **3** (g, h): temperature dependences of the $\chi_M T$ product at H_{dc} = 1000 Oe (left panel) and the field dependences of molar magnetization, M, at the indicated temperatures (right panel). In (a-f), the experimental data are compared with the theoretical ones, obtained using *ab initio* calculations (a, b, e, f – **L** model for Yb^{III} complexes, c, d – **L** model for Yb2 and **L+** model for Yb1).

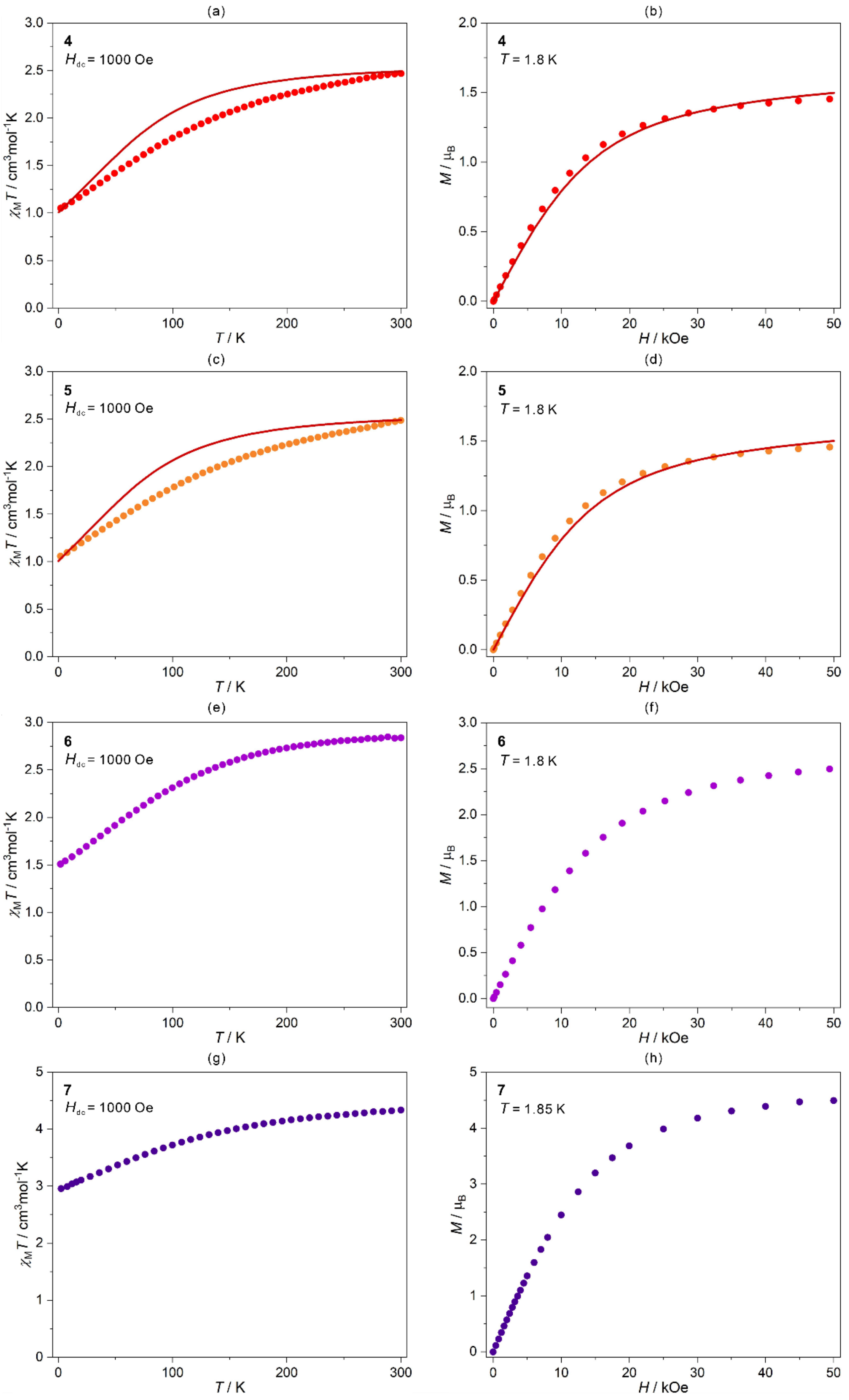

Figure S10. Direct-current (*dc*) magnetic properties of **4** (a, b), **5** (c, d), **6** (e, f) and **7** (g, h): temperature dependences of the $\chi_M T$ product at H_{dc} = 1000 Oe (left panel) and the field dependences of molar magnetization, M, at the indicated temperatures (right panel). In (a-d), the experimental data are compared with the theoretical ones, obtained using *ab initio* calculations (**L** model).

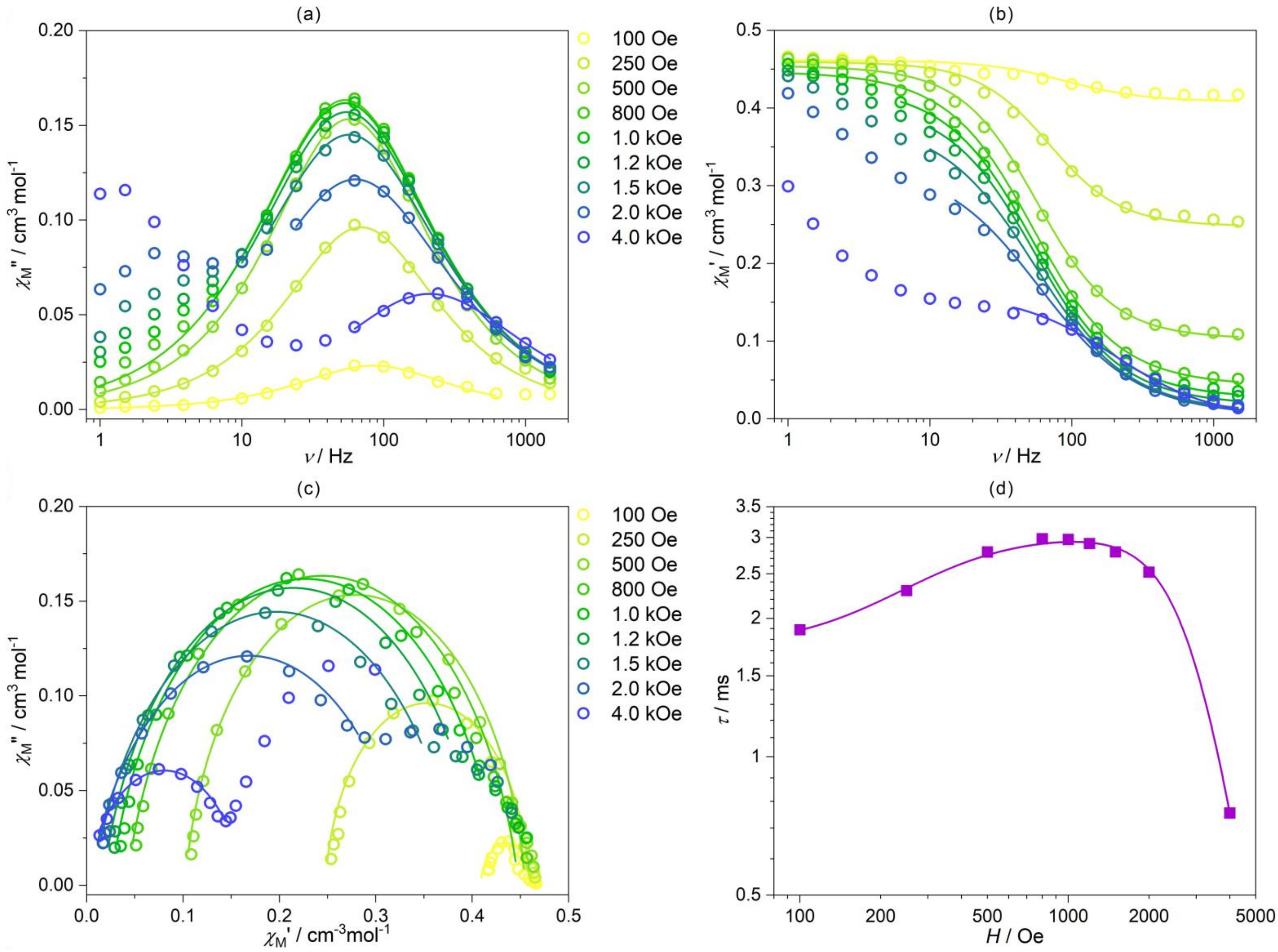

Figure S11. Complete magnetic-field-variable alternate-current (*ac*) magnetic susceptibility characteristics of **1** under H_{ac} = 3 Oe at T = 1.85 K, and their analysis: (a) the frequency dependence of the out-of-phase molar susceptibility, χ_M'', under various indicated *dc* fields, (b) the frequency dependence of the in-phase molar susceptibility, χ_M', under various indicated *dc* fields, (c) the related Argand plots, (d) the field dependence of the relaxation time, τ. Colored solid curves in (a), (b), and (c) represent the best fits using the generalized Debye model for a single relaxation process. The solid purple line in (d) shows the best fit taking into account quantum tunneling of magnetization (QTM), two-phonon Raman process, and field-induced direct process.

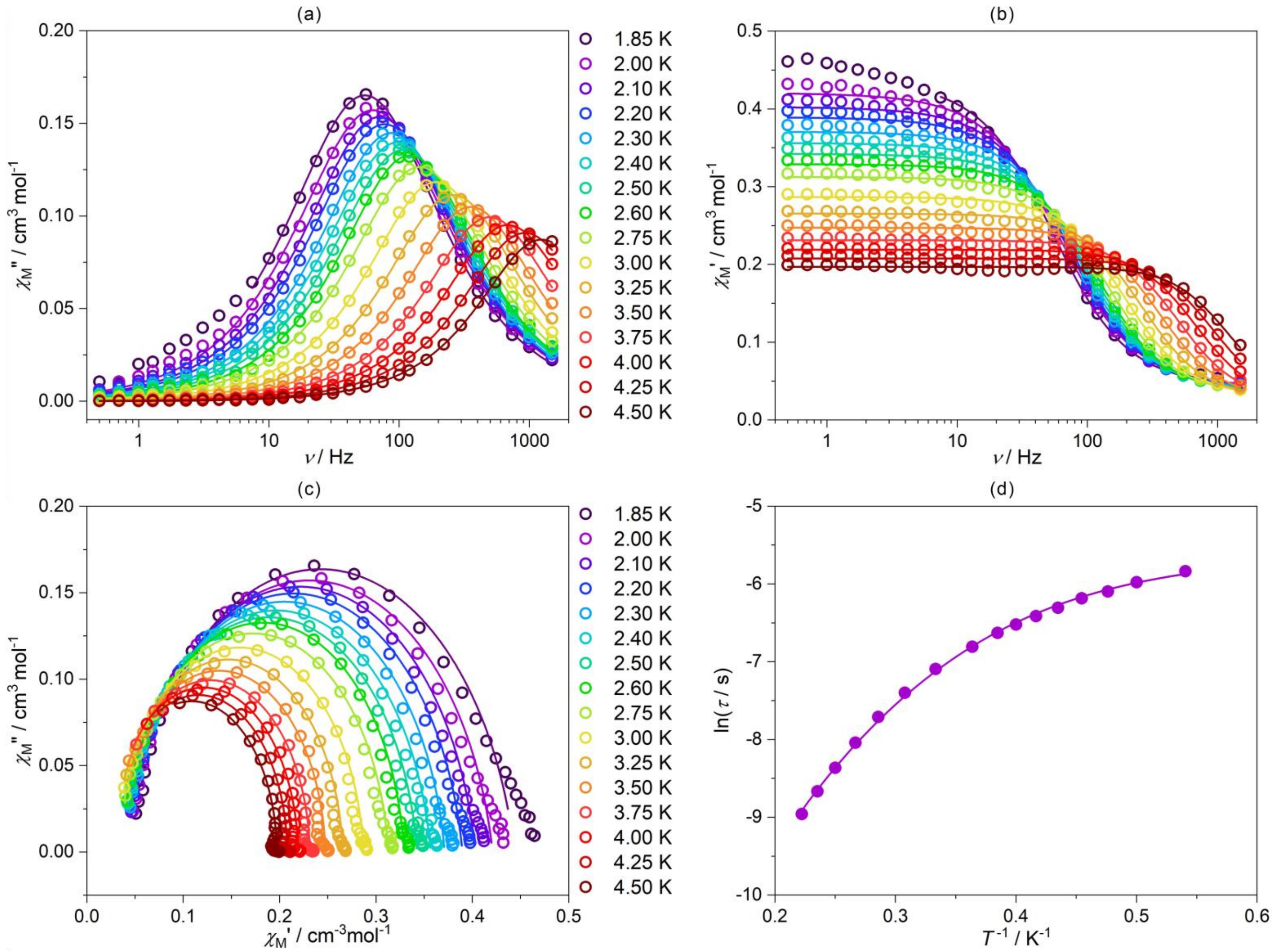

Figure S12. Complete temperature-variable alternate-current (*ac*) magnetic susceptibility characteristics of **1** under H_{ac} = 3 Oe, H_{dc} = 800 Oe, and their analysis: (a) the frequency dependence of the out-of-phase molar susceptibility, χ_M”, at various indicated temperatures, (b) the frequency dependence of the in-phase molar susceptibility, χ_M’, at various indicated temperatures, (c) the related Argand plots, (d) the temperature dependence of the relaxation time, τ. Colored solid curves in (a), (b), and (c) represent the best fits using the generalized Debye model for a single relaxation process. The solid purple line in (d) shows the best fit taking into account quantum tunneling of magnetization (QTM), two-phonon Raman process, and field-induced direct process, in the 1.85–4.5 K range.

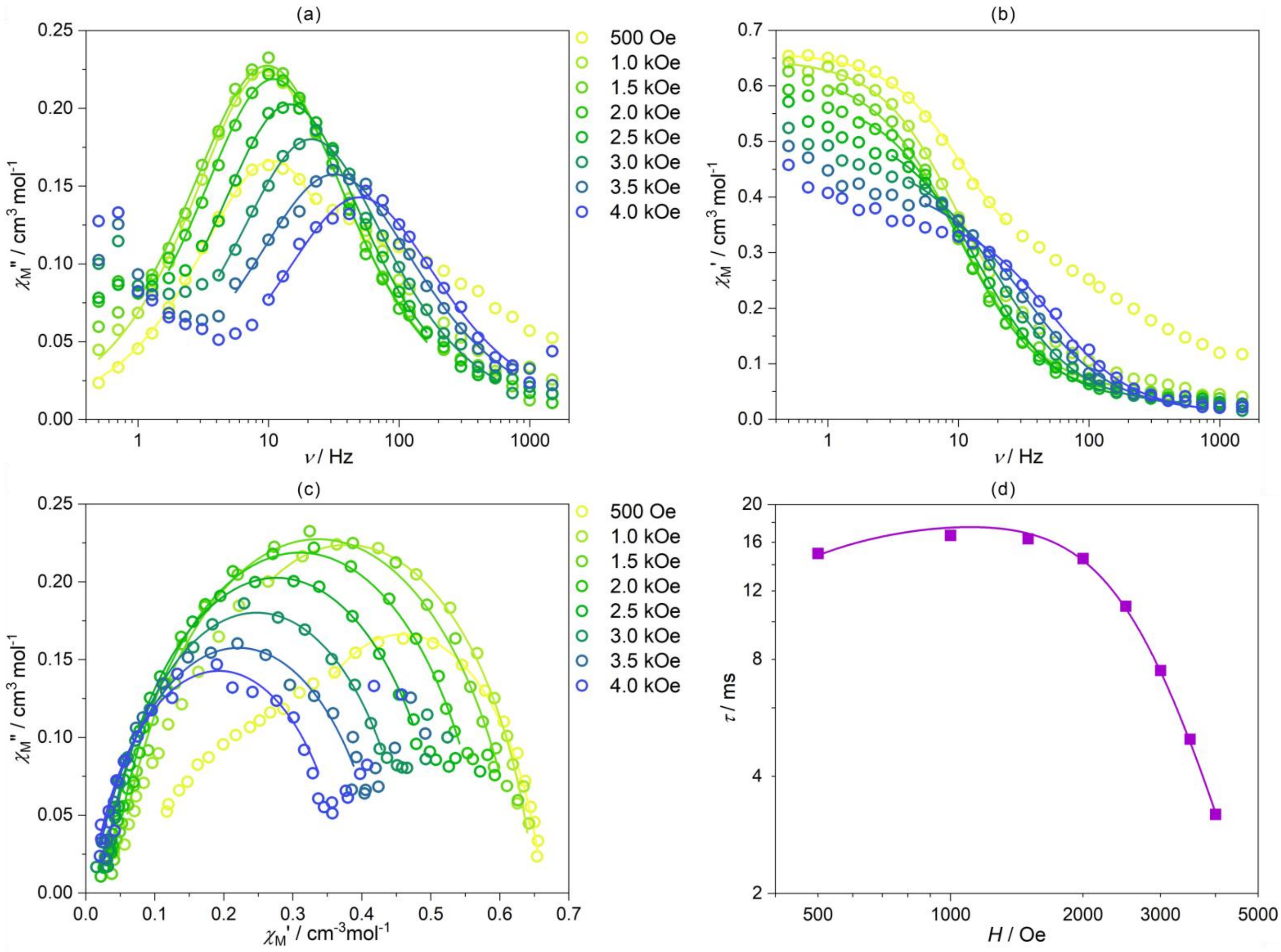

Figure S13. Complete magnetic-field-variable alternate-current (*ac*) magnetic susceptibility characteristics of **2** under H_{ac} = 3 Oe at T = 2 K, and their analysis: (a) the frequency dependence of the out-of-phase molar susceptibility, χ_M'', under various indicated *dc* fields, (b) the frequency dependence of the in-phase molar susceptibility, χ_M', under various indicated *dc* fields, (c) the related Argand plots, (d) the field dependence of the relaxation time, τ. Colored solid curves in (a), (b), and (c) represent the best fits using the generalized Debye model for a single relaxation process. The solid purple line in (d) shows the best fit taking into account quantum tunneling of magnetization (QTM), two-phonon Raman process, and field-induced direct process.

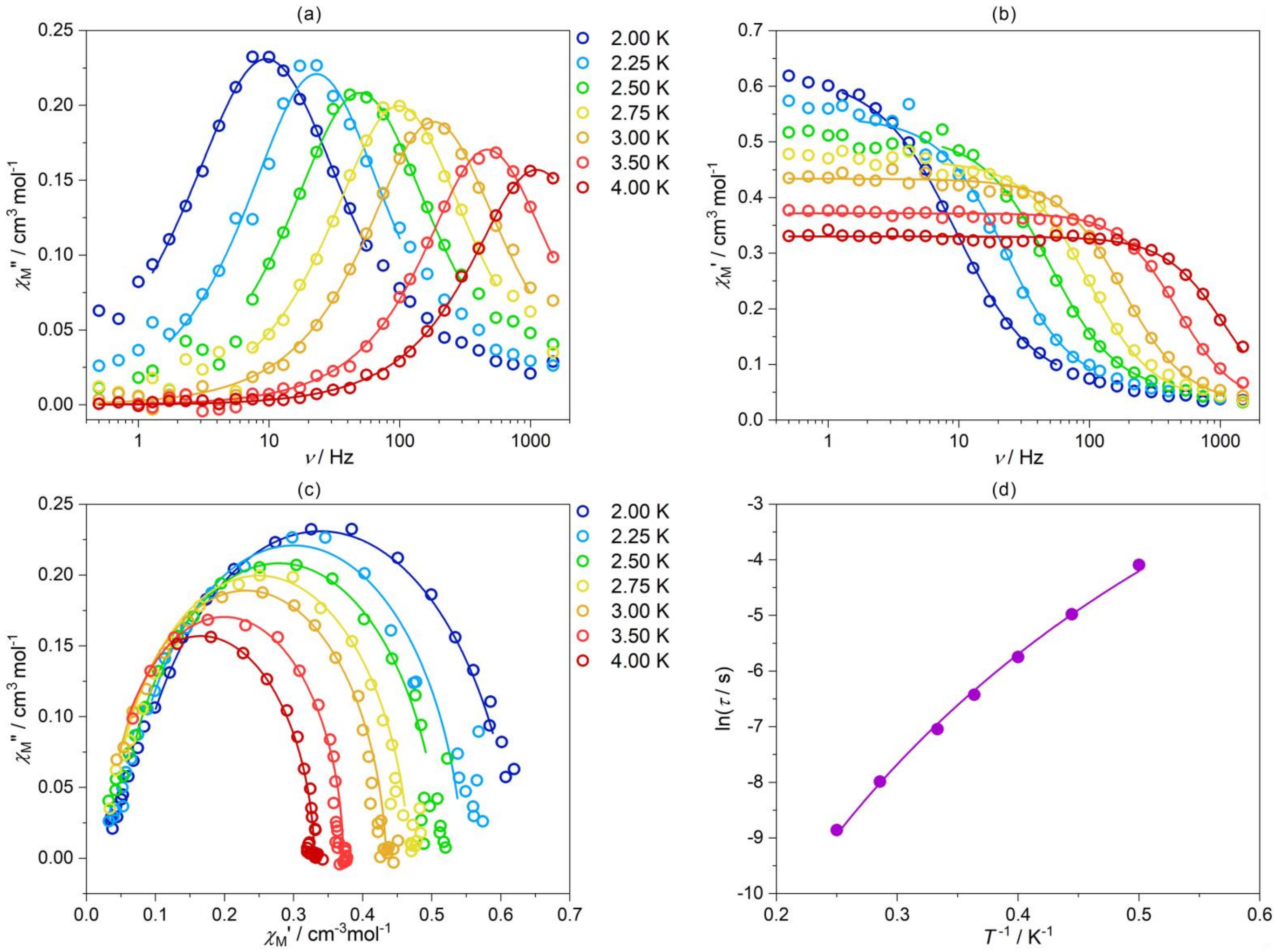

Figure S14. Complete temperature-variable alternate-current (*ac*) magnetic susceptibility characteristics of **2** under H_{ac} = 3 Oe, H_{dc} = 1.5 kOe, and their analysis: (a) the frequency dependence of the out-of-phase molar susceptibility, χ_M", at various indicated temperatures, (b) the frequency dependence of the in-phase molar susceptibility, χ_M', at various indicated temperatures, (c) the related Argand plots, (d) the temperature dependence of the relaxation time, τ. Colored solid curves in (a), (b), and (c) represent the best fits using the generalized Debye model for a single relaxation process. The solid purple line in (d) shows the best fit taking into account quantum tunneling of magnetization (QTM), two-phonon Raman process, and field-induced direct process, in the 2–4 K range.

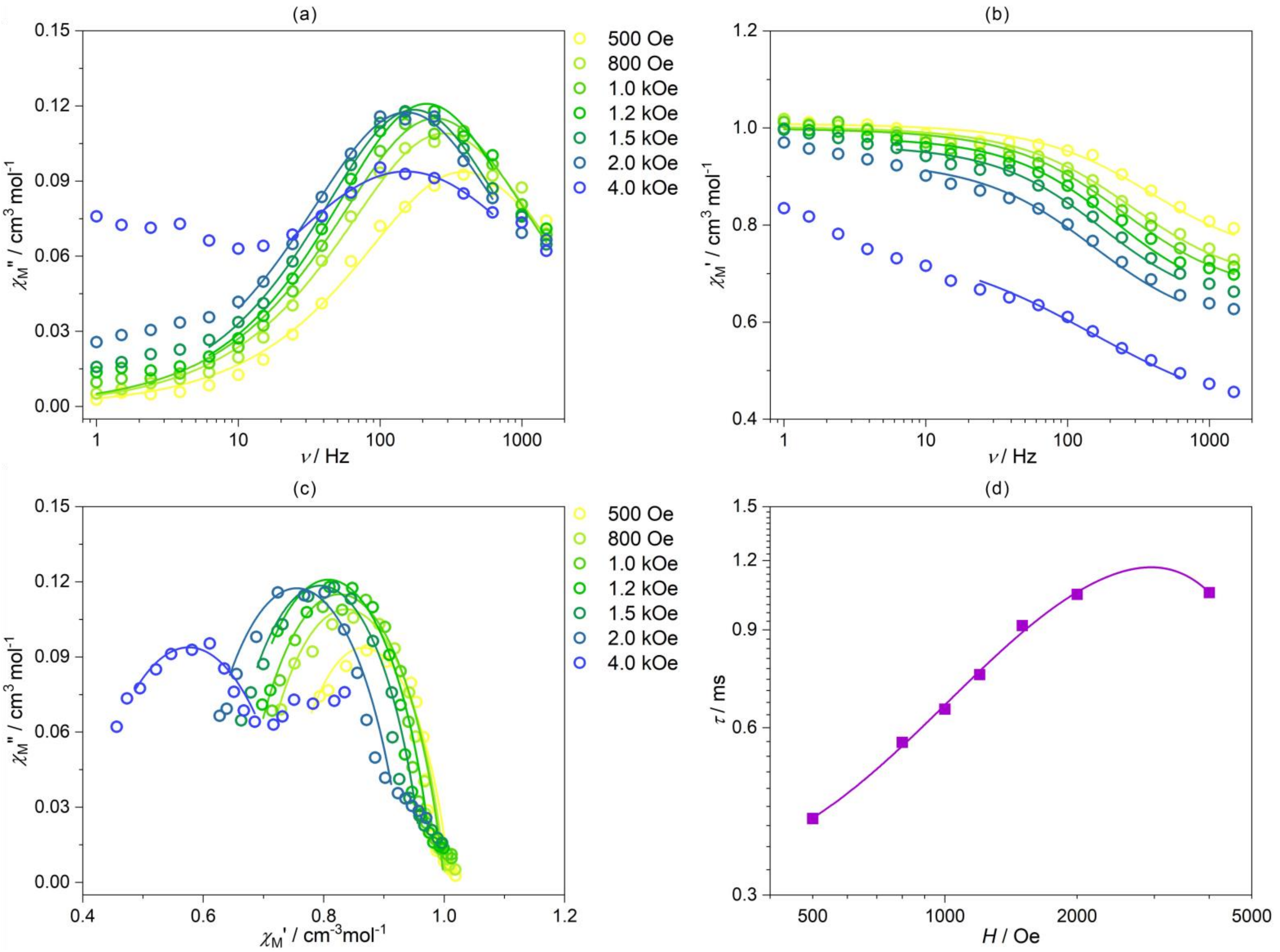

Figure S15. Complete magnetic-field-variable alternate-current (*ac*) magnetic susceptibility characteristics of **3** under H_{ac} = 3 Oe at T = 1.85 K, and their analysis: (a) the frequency dependence of the out-of-phase molar susceptibility, χ_M'', under various indicated *dc* fields, (b) the frequency dependence of the in-phase molar susceptibility, χ_M', under various indicated *dc* fields, (c) the related Argand plots, (d) the field dependence of the relaxation time, τ. Colored solid curves in (a), (b), and (c) represent the best fits using the generalized Debye model for a single relaxation process. The solid purple line in (d) shows the best fit taking into account quantum tunneling of magnetization (QTM), two-phonon Raman process, and field-induced direct process.

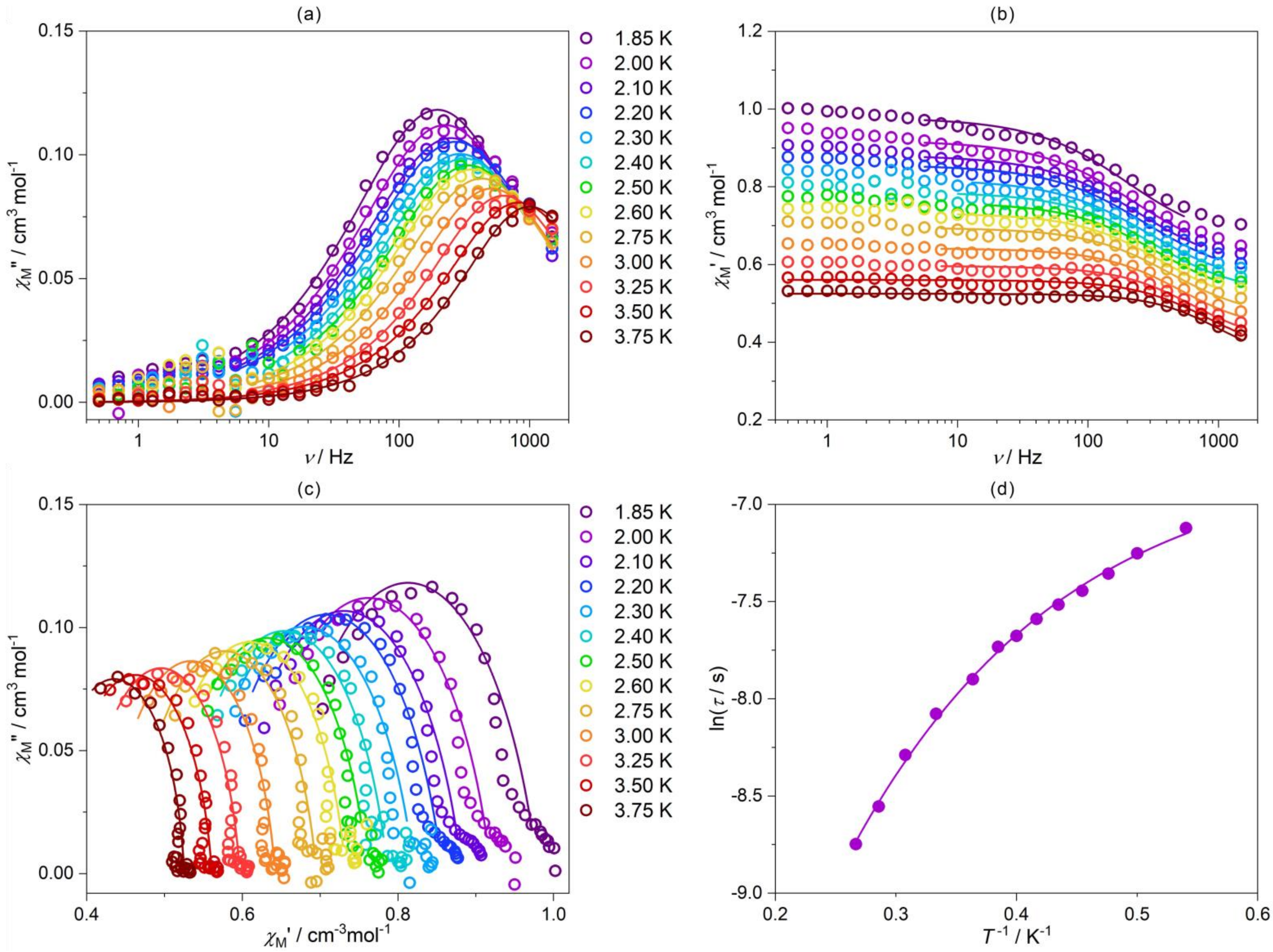

Figure S16. Complete temperature-variable alternate-current (*ac*) magnetic susceptibility characteristics of **3** under H_{ac} = 3 Oe, H_{dc} = 1.2 kOe, and their analysis: (a) the frequency dependence of the out-of-phase molar susceptibility, χ_M'', at various indicated temperatures, (b) the frequency dependence of the in-phase molar susceptibility, χ_M', at various indicated temperatures, (c) the related Argand plots, (d) the temperature dependence of the relaxation time, τ. Colored solid curves in (a), (b), and (c) represent the best fits using the generalized Debye model for a single relaxation process. The solid purple line in (d) shows the best fit taking into account quantum tunneling of magnetization (QTM), two-phonon Raman process, and field-induced direct process, in the 1.85–3.75 K range.

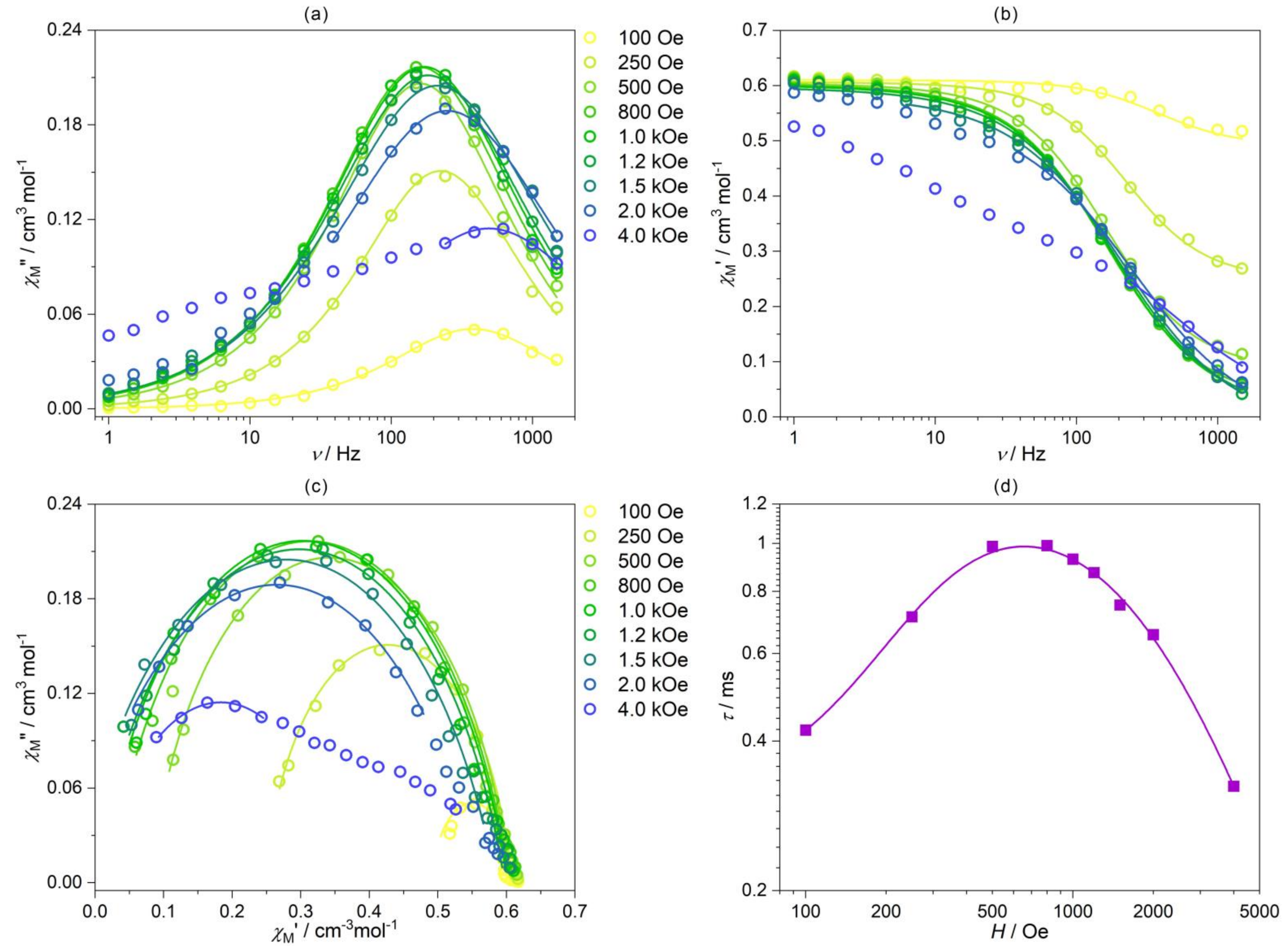

Figure S17. Complete magnetic-field-variable alternate-current (*ac*) magnetic susceptibility characteristics of **4** under H_{ac} = 3 Oe at T = 1.85 K, and their analysis: (a) the frequency dependence of the out-of-phase molar susceptibility, χ_M'', under various indicated *dc* fields, (b) the frequency dependence of the in-phase molar susceptibility, χ_M', under various indicated *dc* fields, (c) the related Argand plots, (d) the field dependence of the relaxation time, τ. Colored solid curves in (a), (b), and (c) represent the best fits using the generalized Debye model for a single relaxation process. The solid purple line in (d) shows the best fit taking into account quantum tunneling of magnetization (QTM), two-phonon Raman process, and field-induced direct process.

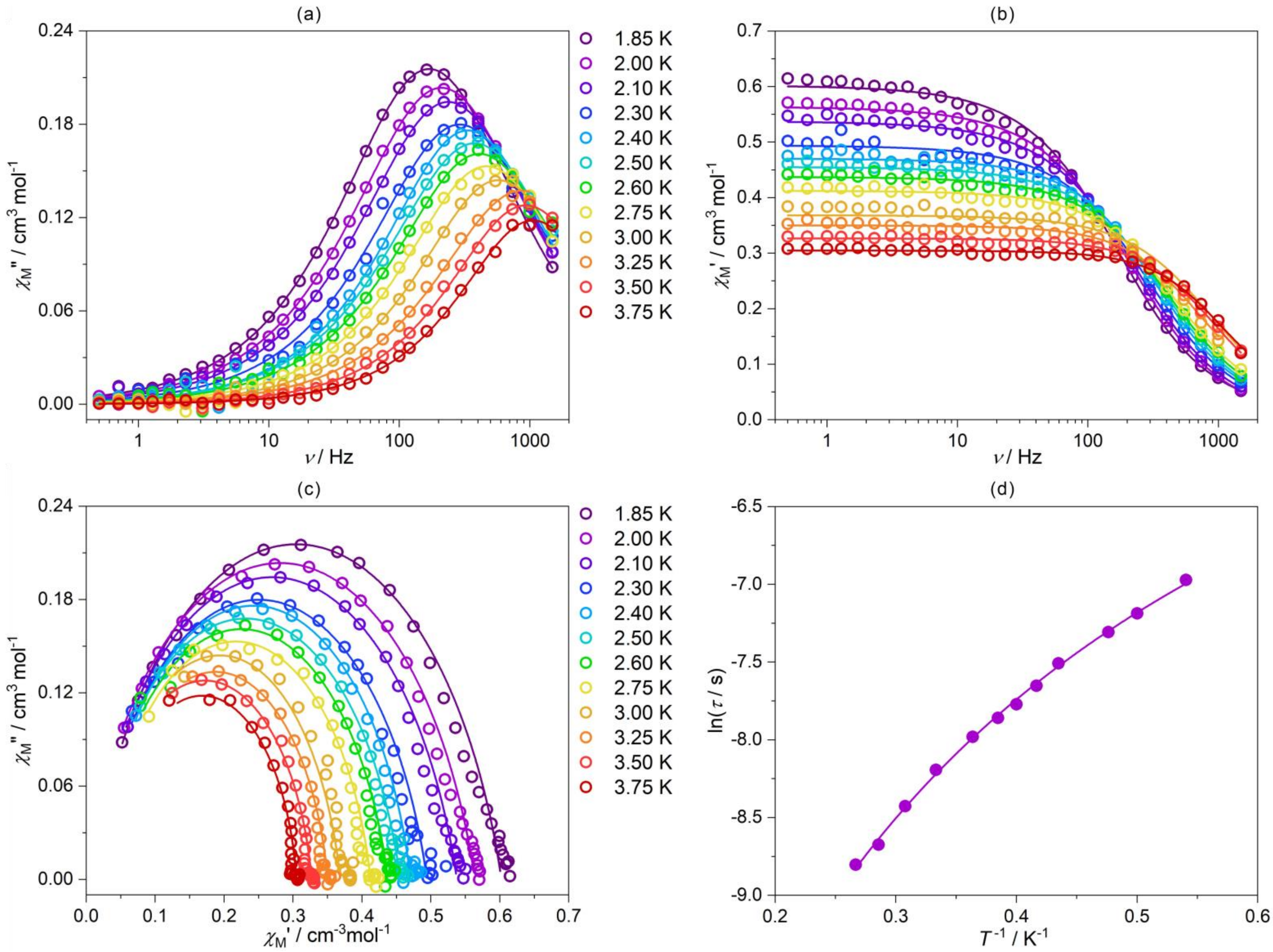

Figure S18. Complete temperature-variable alternate-current (*ac*) magnetic susceptibility characteristics of **4** under H_{ac} = 3 Oe, H_{dc} = 1 kOe, and their analysis: (a) the frequency dependence of the out-of-phase molar susceptibility, χ_M", at various indicated temperatures, (b) the frequency dependence of the in-phase molar susceptibility, χ_M', at various indicated temperatures, (c) the related Argand plots, (d) the temperature dependence of the relaxation time, τ. Colored solid curves in (a), (b), and (c) represent the best fits using the generalized Debye model for a single relaxation process. The solid purple line in (d) shows the best fit taking into account quantum tunneling of magnetization (QTM), two-phonon Raman process, and field-induced direct process, in the 1.85–3.75 K range.

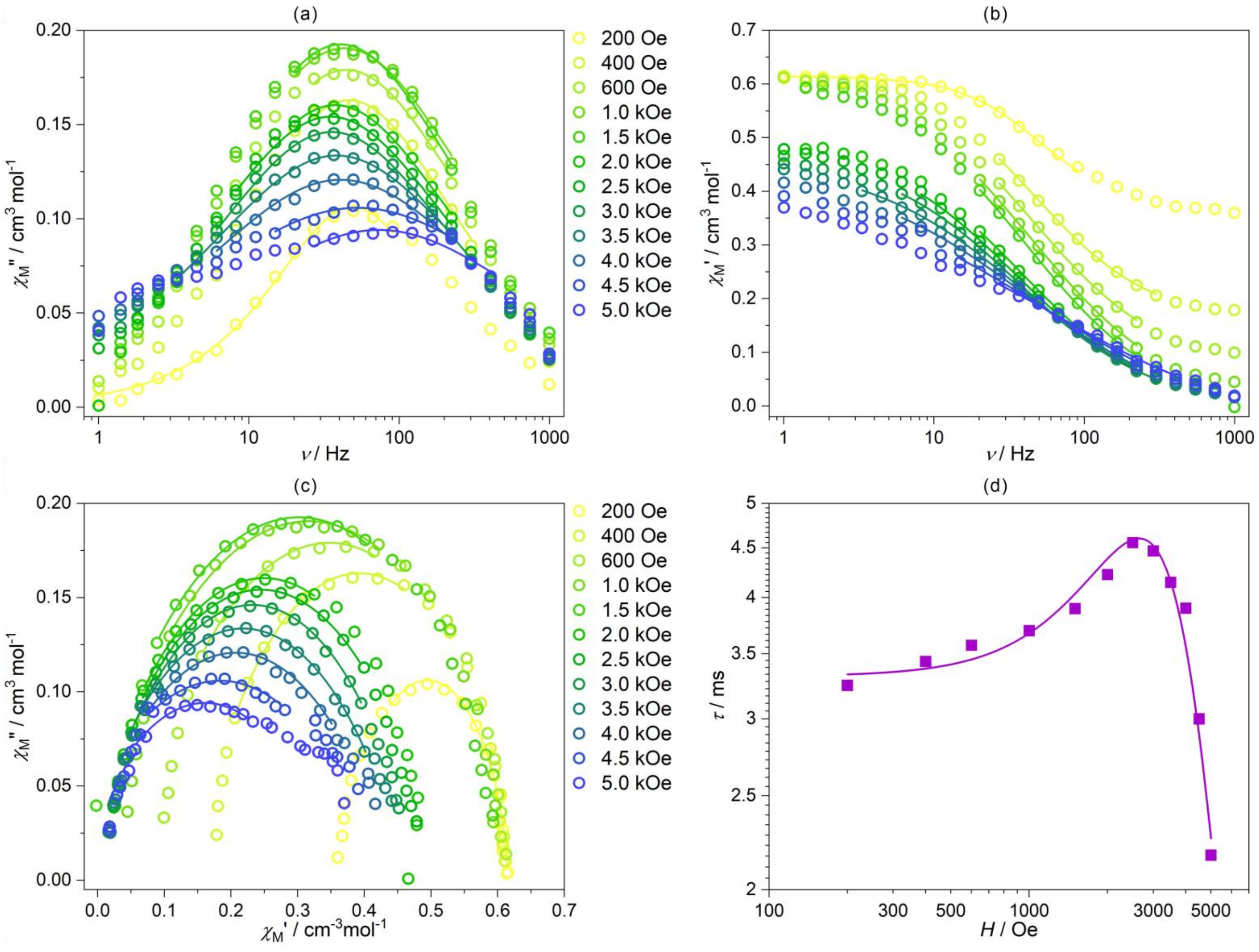

Figure S19. Complete magnetic-field-variable alternate-current (*ac*) magnetic susceptibility characteristics of **5** under H_{ac} = 3 Oe at T = 1.8 K, and their analysis: (a) the frequency dependence of the out-of-phase molar susceptibility, χ_M'', under various indicated *dc* fields, (b) the frequency dependence of the in-phase molar susceptibility, χ_M', under various indicated *dc* fields, (c) the related Argand plots, (d) the field dependence of the relaxation time, τ. Colored solid curves in (a), (b), and (c) represent the best fits using the generalized Debye model for a single relaxation process. The solid purple line in (d) shows the best fit taking into account quantum tunneling of magnetization (QTM), two-phonon Raman process, and field-induced direct process.

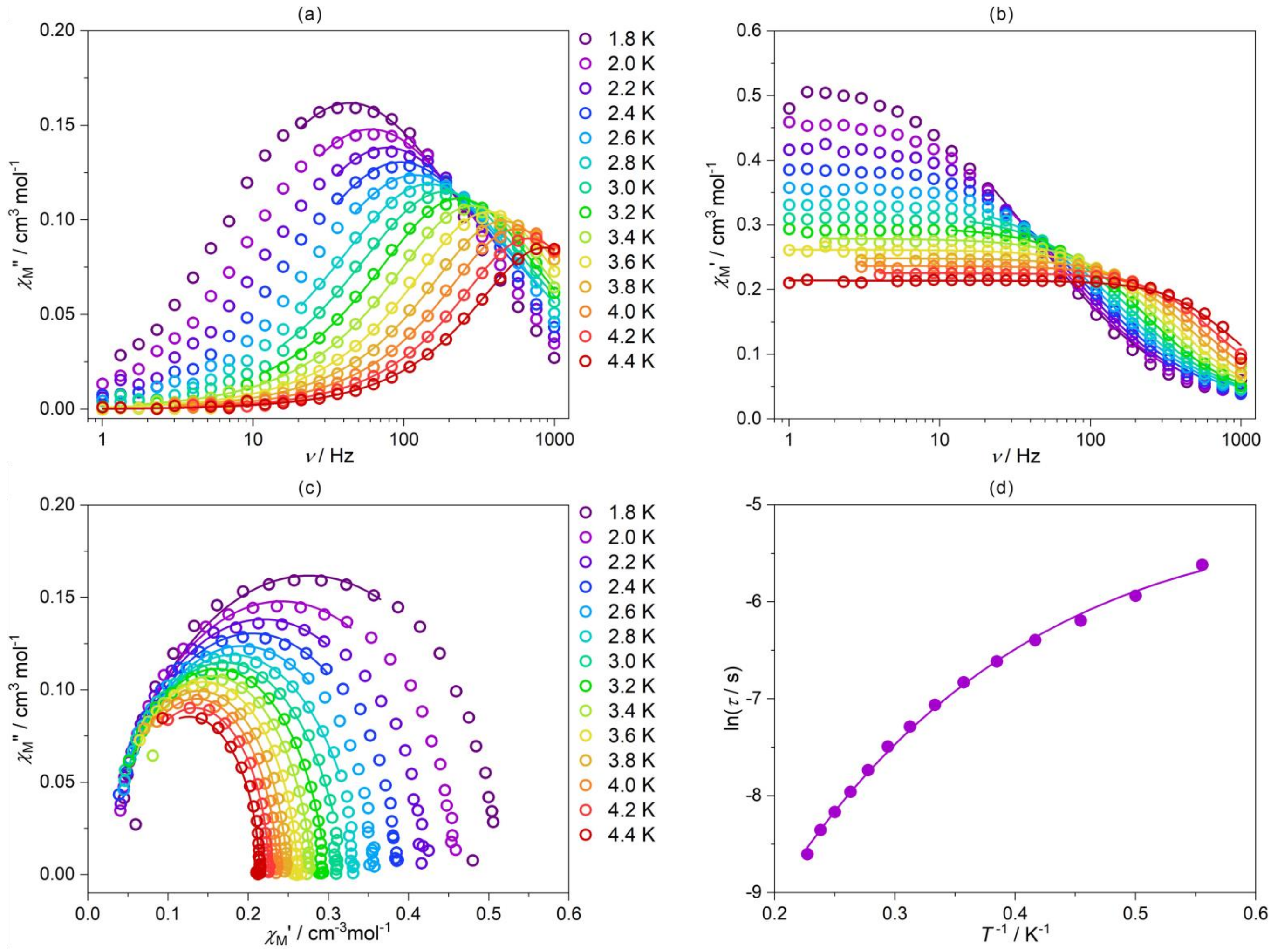

Figure S20. Complete temperature-variable alternate-current (*ac*) magnetic susceptibility characteristics of **5** under H_{ac} = 3 Oe, H_{dc} = 1 kOe, and their analysis: (a) the frequency dependence of the out-of-phase molar susceptibility, χ_M", at various indicated temperatures, (b) the frequency dependence of the in-phase molar susceptibility, χ_M', at various indicated temperatures, (c) the related Argand plots, (d) the temperature dependence of the relaxation time, τ. Colored solid curves in (a), (b), and (c) represent the best fits using the generalized Debye model for a single relaxation process. The solid purple line in (d) shows the best fit taking into account quantum tunneling of magnetization (QTM), two-phonon Raman process, and field-induced direct process, in the 1.8–4.4 K range.

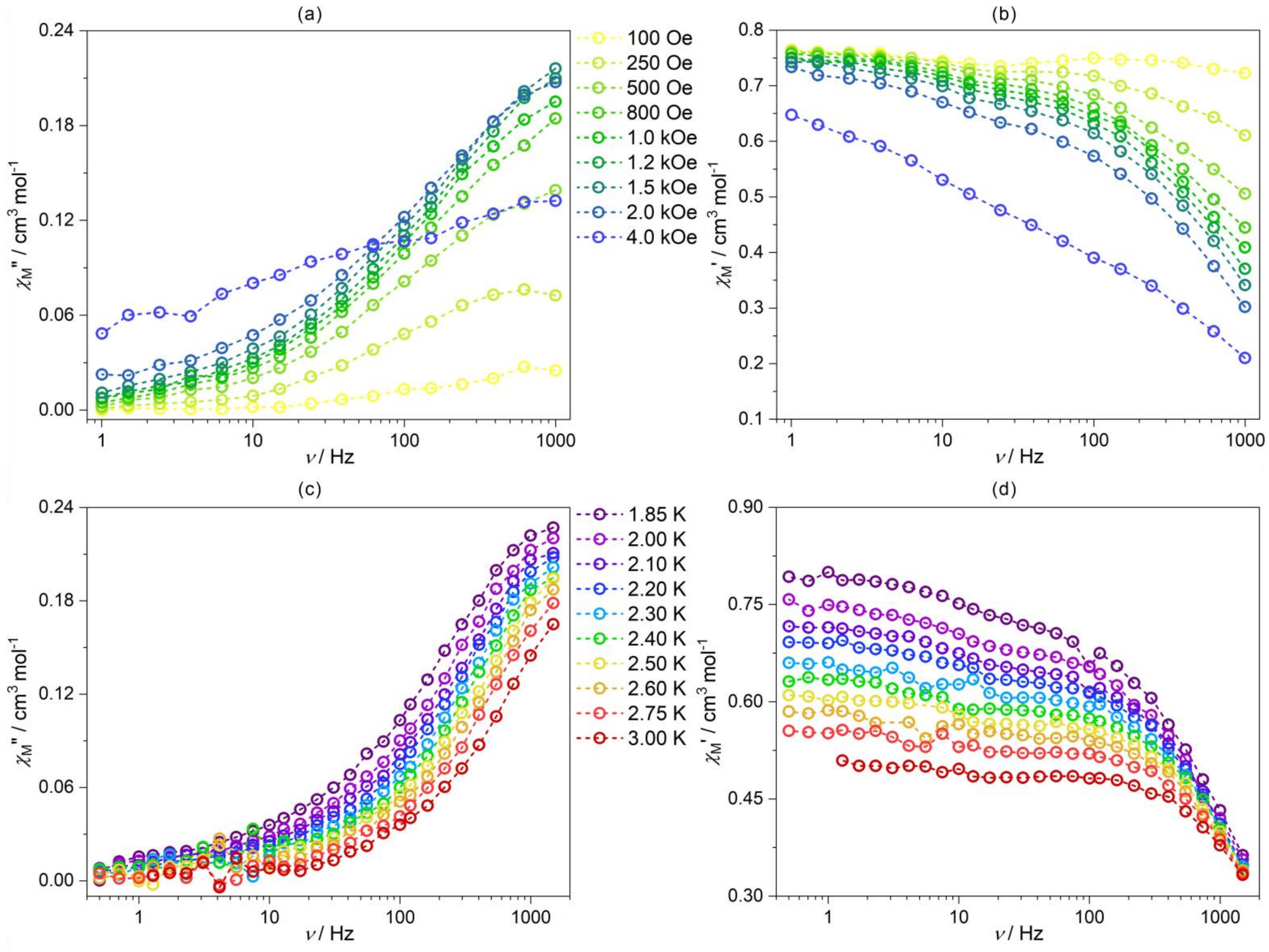

Figure S21. Field- and temperature-variable alternate-current (*ac*) magnetic susceptibility characteristics of **6** under H_{ac} = 3 Oe and their analysis: (a) the frequency dependence of the out-of-phase molar susceptibility, χ_M'', at various indicated fields measured at T = 1.85 K, (b) the frequency dependence of the in-phase molar susceptibility, χ_M', at various indicated fields measured at T = 1.85 K, (c) the frequency dependence of the out-of-phase molar susceptibility, χ_M'', at various indicated temperatures measured under H_{dc} = 1.5 kOe, (d) the frequency dependence of the in-phase molar susceptibility, χ_M', at various indicated temperatures measured under H_{dc} = 1.5 kOe.

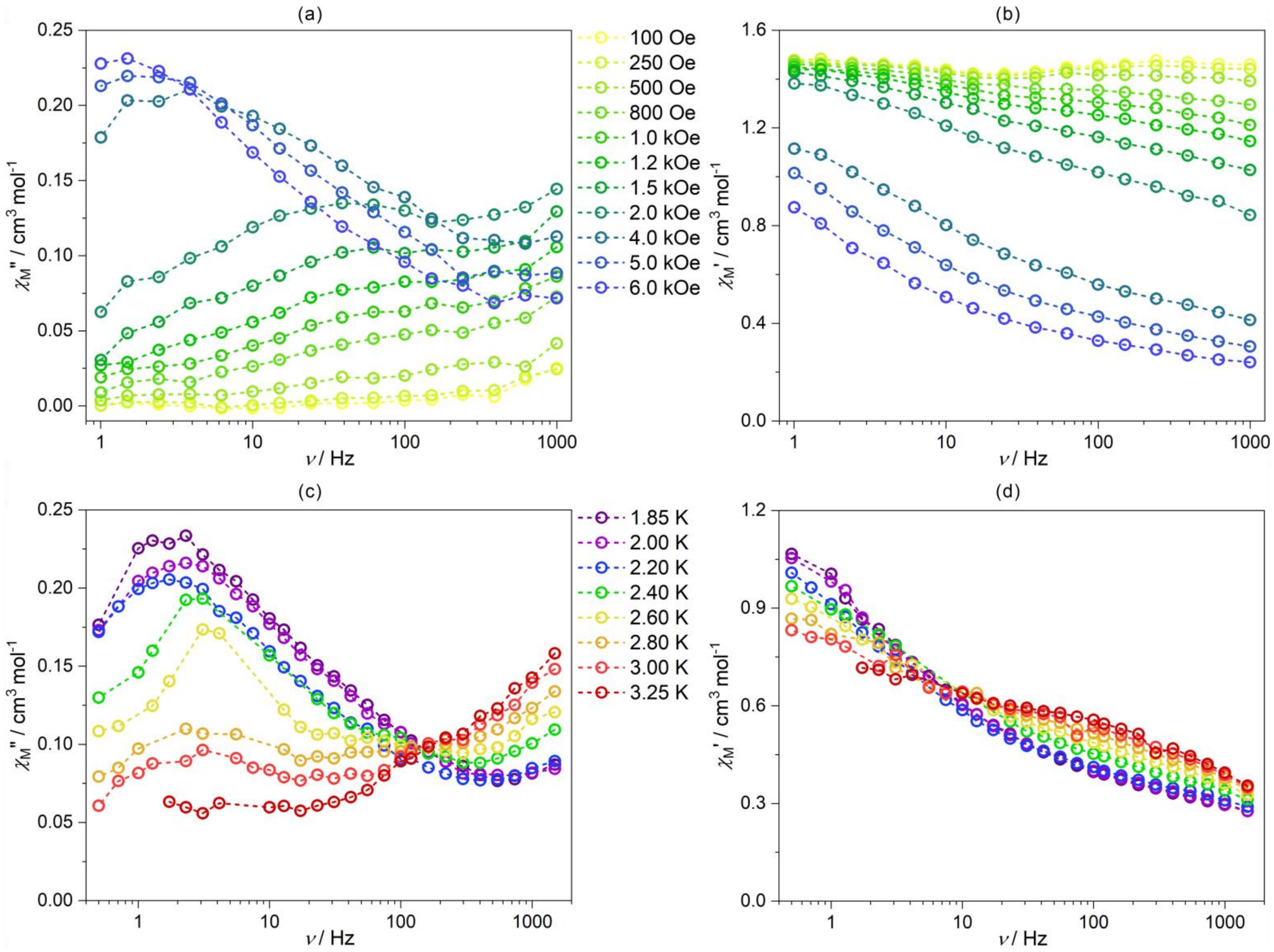

Figure S22. Field- and temperature-variable alternate-current (*ac*) magnetic susceptibility characteristics of **7** under H_{ac} = 3 Oe and their analysis: (a) the frequency dependence of the out-of-phase molar susceptibility, χ_M", at various indicated fields measured at T = 1.85 K, (b) the frequency dependence of the in-phase molar susceptibility, χ_M', at various indicated fields measured at T = 1.85 K, (c) the frequency dependence of the out-of-phase molar susceptibility, χ_M", at various indicated temperatures measured under H_{dc} = 5 kOe, (d) the frequency dependence of the in-phase molar susceptibility, χ_M', at various indicated temperatures measured under H_{dc} = 5 kOe.

Comment to Figures S11–S20

For the fitting of the frequency dependences of χ_M' and χ_M" contributions to the *ac* magnetic susceptibility and the related Argand χ_M"(χ_M') plots (Figures S11–S20), the following equations (eq1 and eq2) of the generalized Debye model for a single relaxation process were used:

$$\chi_M'(\omega) = \chi_S + (\chi_T - \chi_S)\frac{1+(\omega\tau)^{1-\alpha}\sin(\pi\alpha/2)}{1+2(\omega\tau)^{1-\alpha}\sin(\pi\alpha/2)+(\omega\tau)^{2(1-\alpha)}} \quad \text{(eq1)}$$

$$\chi_M''(\omega) = (\chi_T - \chi_S)\frac{(\omega\tau)^{1-\alpha}\cos(\pi\alpha/2)}{1+2(\omega\tau)^{1-\alpha}\sin(\pi\alpha/2)+(\omega\tau)^{2(1-\alpha)}} \quad \text{(eq2)}$$

where

χ_S = the adiabatic susceptibility (at the infinitely high frequency of *ac* field),

χ_T = the isothermal susceptibility (at the infinitely low frequency of *ac* field),

τ = the relaxation time,

α = the distribution (Cole-Cole) parameter,

and ω is an angular frequency, that is $\omega = 2\pi v$, with v being for the linear frequency in [Hz] units.[9,10]

Table S8. Summary of parameters extracted from the fitting of *H*- and *T*-dependences of the relaxation times in **1**–**5** including contributions to the overall relaxation time from all considered processes at the indicated conditions.

compound	**1**	**2**	**3**	**4**	**5**
overall fitting with QTM, Raman, and Direct relaxation processes					
A_{dir} / $s^{-1}K^{-1}Oe^{-m}$	$2.11(2)\cdot10^{-12}$	$5.03(6)\cdot10^{-13}$	$5(1)\cdot10^{-13}$	$2.3(6)\cdot10^{-3}$	$2.7(1)\cdot10^{-13}$
m	4 (fixed)	4 (fixed)	4 (fixed)	1.59(3)	4 (fixed)
$A_{dir}H^m$ / $s^{-1}K^{-1}$ (for optimal *dc* field)	0.86(1)	2.55(3)	1.0(2)	135(35)	0.27(1)
τ_{Direct} / s (optimal *dc* field, T = 1.8 K)	0.65(1)	0.218(3)	0.55(2)	$4.1(8)\cdot10^{-3}$	2.1(1)
B_1 / s^{-1}	497(14)	2000 (fixed)	2861(158)	2365(115)	190(8)
B_2 / Oe^{-2}	$2.3(4)\cdot10^{-5}$	$5(1)\cdot10^{-4}$	$2.2(3)\cdot10^{-6}$	$4.8(6)\cdot10^{-5}$	$1.8(3)\cdot10^{-7}$
B_3 / Oe^{-2}	$1.1(1)\cdot10^{-5}$	–	$1.0(4)\cdot10^{-7}$	–	-
τ_{QTM} / s	$4(1)\cdot10^{-3}$	0.6(1)	$1.3(3)\cdot10^{-3}$	$2.1(4)\cdot10^{-2}$	$6.2(4)\cdot10^{-3}$
C_{Raman} / $s^{-1}K^{-n}$	4.4(3)	0.50(8)	58(3)	131(5)	12.1(9)
n	4.92(5)	6.9(2)	3.43(5)	2.93(4)	4.06(6)
τ_{Raman} / s (T = 1.8 K)	0.013(2)	0.035(6)	$2.3(1)\cdot10^{-3}$	$1.36(6)\cdot10^{-3}$	$7.6(4)\cdot10^{-3}$
limited fitting according to the Arrhenius law					
τ_0 / s	$1.4(2)\cdot10^{-6}$	$7(2)\cdot10^{-7}$	$1.0(2)\cdot10^{-5}$	$1.2(2)\cdot10^{-5}$	$3(1)\cdot10^{-6}$
U_{eff} / K	20.6(5)	22(1)	10.2(5)	9.4(5)	18(1)

Table S9. Description with contractions of the basis sets (models **S**, **L**, and **L+**) employed in *ab initio* calculations of the Yb^{III} crystal field in **1, 2, 4**, and **5**.

Basis set in model **S**	Basis set in models **L** and **L+**
Yb.ANO-RCC-VDZP 7S6P4D2F1G	Yb.ANO-RCC-VTZP 8S7P5D3F2G1H
N.ANO-RCC-VDZ 3S2P	N.ANO-RCC-VDZ 3S2P
O.ANO-RCC-VDZ 3S2P	O.ANO-RCC-VDZP 3S2P1D
C.ANO-RCC-VDZ 3S2P	C.ANO-RCC-VDZ 3S2P
H.ANO-RCC-VDZ 2S	H.ANO-RCC-VDZ 2S

Table S10. Summary of the energy splitting of the $^{2}F_{7/2}$ multiplet of Yb(III) for Yb1 centers in **1** and pseudo-*g*-tensors of ground Kramers doublet with state composition in $|m_J\rangle$ basis (model **S** and **L**).

**1** (Yb1 centers)	
model **S**	model **L**
**energy / cm^{-1}**	
0.000	0.000
103.270	138.747
251.933	255.213
425.440	391.717
**pseudo-*g*-tensor components (g_x, g_y, g_z) of ground Kramers doublet**	
0.813	0.596
1.276	0.897
5.858	6.002
**composition of ground Kramers doublet in $|m_J\rangle$ basis on the quantization axis within *J* = 7/2 manifold**	
0.5% $|+7/2\rangle$<br>2.0% $|+5/2\rangle$<br>0.2% $|+3/2\rangle$<br>1.5% $|+1/2\rangle$<br>4.2% $|-1/2\rangle$<br>13.7% $|-3/2\rangle$<br>40.2% $|-5/2\rangle$<br>37.7% $|-7/2\rangle$	30.5% $|+7/2\rangle$<br>37.0% $|+5/2\rangle$<br>9.6% $|+3/2\rangle$<br>3.3% $|+1/2\rangle$<br>0.3% $|-1/2\rangle$<br>4.8% $|-3/2\rangle$<br>6.5% $|-5/2\rangle$<br>8.0% $|-7/2\rangle$

Table S11. Summary of the energy splitting of the $^2F_{7/2}$ multiplet of Yb(III) for Yb2 centers in **1** and pseudo-*g*-tensors of ground Kramers doublet with state composition in $|m_J\rangle$ basis.

**1** (Yb2 centers)	
model **S**	model **L**
**energy / cm^{-1}**	
0.000	0.000
156.000	179.399
267.340	264.961
431.483	396.928
**pseudo-*g*-tensor components (g_x, g_y, g_z) of ground Kramers doublet**	
0.408	0.272
0.672	0.642
6.740	6.486
**composition of ground Kramers doublet in $|m_J\rangle$ basis on the quantization axis within *J* = 7/2 manifold**	
1.2% $|+7/2\rangle$<br>0.1% $|+5/2\rangle$<br>0.4% $|+3/2\rangle$<br>1.3% $|+1/2\rangle$<br>8.0% $|-1/2\rangle$<br>6.9% $|-3/2\rangle$<br>8.7% $|-5/2\rangle$<br>73.5% $|-7/2\rangle$	0.0% $|+7/2\rangle$<br>0.2% $|+5/2\rangle$<br>0.1% $|+3/2\rangle$<br>1.2% $|+1/2\rangle$<br>6.8% $|-1/2\rangle$<br>11.0% $|-3/2\rangle$<br>16.8% $|-5/2\rangle$<br>63.9% $|-7/2\rangle$

Table S12. Summary of the energy splitting of the $^2F_{7/2}$ multiplet of Yb(III) for Yb1 centers in **1** and pseudo-*g*-tensors of ground Kramers doublet with state composition in $|m_J\rangle$ basis (model **L** and **L+**).

**1** (Yb1 centers)	
model **L**	model **L+**
**energy / cm^{-1}**	
0.000	0.000
138.747	158.868
255.213	273.754
391.717	421.747
**pseudo-*g*-tensor components (g_x, g_y, g_z) of ground Kramers doublet**	
0.596	0.598
0.897	0.878
6.002	5.950
**composition of ground Kramers doublet in $|m_J\rangle$ basis on the quantization axis within *J* = 7/2 manifold**	
30.5% $|+7/2\rangle$<br>37.0% $|+5/2\rangle$<br>9.6% $|+3/2\rangle$<br>3.3% $|+1/2\rangle$<br>0.3% $|-1/2\rangle$<br>4.8% $|-3/2\rangle$<br>6.5% $|-5/2\rangle$<br>8.0% $|-7/2\rangle$	33.6% $|+7/2\rangle$<br>46.5% $|+5/2\rangle$<br>13.8% $|+3/2\rangle$<br>2.2% $|+1/2\rangle$<br>0.9% $|-1/2\rangle$<br>0.0% $|-3/2\rangle$<br>2.3% $|-5/2\rangle$<br>0.6% $|-7/2\rangle$

Table S13. Summary of the energy splitting of the $^2F_{7/2}$ multiplet of Yb(III) in **2** and pseudo-*g*-tensors of ground Kramers doublet with state composition in $|m_J\rangle$ basis.

**2**	
model **S**	model **L**
**energy / cm^{-1}**	
0.000	0.000
113.066	152.955
284.101	285.028
453.254	407.312
**pseudo-*g*-tensor components (g_x, g_y, g_z) of ground Kramers doublet**	
0.132	0.178
0.413	0.387
6.989	6.704
**composition of ground Kramers doublet in $|m_J\rangle$ basis on the quantization axis within *J* = 7/2 manifold**	
33.7% $|+7/2\rangle$<br>3.3% $|+5/2\rangle$<br>3.8% $|+3/2\rangle$<br>4.6% $|+1/2\rangle$<br>1.5% $|-1/2\rangle$<br>4.0% $|-3/2\rangle$<br>2.3% $|-5/2\rangle$<br>46.7% $|-7/2\rangle$	51.1% $|+7/2\rangle$<br>10.2% $|+5/2\rangle$<br>10.8% $|+3/2\rangle$<br>5.1% $|+1/2\rangle$<br>0.0% $|-1/2\rangle$<br>2.0% $|-3/2\rangle$<br>1.9% $|-5/2\rangle$<br>18.8% $|-7/2\rangle$

Table S14. Summary of the energy splitting of the $^2F_{7/2}$ multiplet of Yb(III) in **4** and pseudo-*g*-tensors of ground Kramers doublet with state composition in $|m_J\rangle$ basis.

**4**	
model **S**	model **L**
**energy / cm^{-1}**	
0.000	0.000
100.174	142.695
211.879	215.494
317.922	297.073
**pseudo-*g*-tensor components (g_x, g_y, g_z) of ground Kramers doublet**	
0.067	0.243
0.827	0.791
5.603	5.614
**composition of ground Kramers doublet in $|m_J\rangle$ basis on the quantization axis within *J* = 7/2 manifold**	
0.3% $|+7/2\rangle$<br>42.1% $|+5/2\rangle$<br>0.1% $|+3/2\rangle$<br>0.0% $|+1/2\rangle$<br>0.0% $|-1/2\rangle$<br>0.1% $|-3/2\rangle$<br>57.1% $|-5/2\rangle$<br>0.2% $|-7/2\rangle$	0.1% $|+7/2\rangle$<br>49.5% $|+5/2\rangle$<br>0.2% $|+3/2\rangle$<br>0.1% $|+1/2\rangle$<br>0.1% $|-1/2\rangle$<br>0.2% $|-3/2\rangle$<br>49.5% $|-5/2\rangle$<br>0.1% $|-7/2\rangle$

Table S15. Summary of the energy splitting of the $^2F_{7/2}$ multiplet of Yb(III) in **5** and pseudo-*g*-tensors of ground Kramers doublet with state composition in $|m_J\rangle$ basis.

**5**	
model **S**	model **L**
**energy / cm^{-1}**	
0.000	0.000
91.850	133.789
220.217	223.330
326.046	306.512
**pseudo-*g*-tensor components (g_x, g_y, g_z) of ground Kramers doublet**	
0.064	0.258
0.896	0.828
5.587	5.606
**composition of ground Kramers doublet in $|m_J\rangle$ basis on the quantization axis within *J* = 7/2 manifold**	
0.1% $|+7/2\rangle$<br>86.7% $|+5/2\rangle$<br>0.0% $|+3/2\rangle$<br>0.0% $|+1/2\rangle$<br>0.0% $|-1/2\rangle$<br>0.2% $|-3/2\rangle$<br>12.4% $|-5/2\rangle$<br>0.6% $|-7/2\rangle$	0.0% $|+7/2\rangle$<br>85.1% $|+5/2\rangle$<br>0.1% $|+3/2\rangle$<br>0.2% $|+1/2\rangle$<br>0.0% $|-1/2\rangle$<br>0.4% $|-3/2\rangle$<br>13.9% $|-5/2\rangle$<br>0.2% $|-7/2\rangle$

Table S16. Energy splitting of ^2F term of Yb^{III} for **1**, **2**, **4**, and **5** in **L** model and **L+** model.

**state**	**energy / cm^{-1}**					
-	**1** (Yb1, **L** model)	**1** (Yb2)	**1** (Yb1, **L+** model)	**2**	**4**	**5**
1	0.000	0.000	0.000	0.000	0.000	0.000
2	0.000	0.000	0.000	0.000	0.000	0.000
3	138.747	179.399	158.868	152.955	142.695	133.789
4	138.747	179.399	158.868	152.955	142.695	133.789
5	255.213	264.961	273.754	285.028	215.494	223.330
6	255.213	264.961	273.754	285.028	215.494	223.330
7	391.717	396.928	421.747	407.312	297.073	306.512
8	391.717	396.928	421.747	407.312	297.073	306.512
**9**	**10349.939**	**10348.623**	**10346.727**	**10355.002**	**10360.708**	**10364.727**
**10**	**10349.939**	**10348.623**	**10346.727**	**10355.002**	**10360.708**	**10364.727**
11	10432.147	10467.676	10436.849	10450.206	10402.609	10395.526
12	10432.147	10467.676	10436.849	10450.206	10402.609	10395.526
13	10655.706	10659.802	10672.431	10677.577	10575.261	10584.863
14	10655.706	10659.802	10672.431	10677.577	10575.261	10584.863

Table S17. Cumulative oscillator strengths for transitions between emissive doublet of $^2F_{5/2}$ excited term (states 9 and 10) and four Kramers doublets of $^2F_{7/2}$ multiplet in velocity gauge together with most intensive hot transitions.

states (as in Table S16)		oscillator strength × 10^6 (velocity gauge)					
from	to	**1** (Yb1, **L** model)	**1** (Yb2)	**1** (Yb1, **L+** model)	**2**	**4**	**5**
(9,10)	(1,2)	2.34	1.15	1.69	1.48	1.49	1.60
(9,10)	(3,4)	8.43	12.10	6.34	8.77	11.10	10.80
(9,10)	(5,6)	10.70	10.40	7.54	11.40	1.25	1.57
(9,10)	(7,8)	4.81	5.13	3.78	5.93	3.80	3.58
(11,12)	(1,2)	8.57	12.20	6.40	9.34	8.08	7.84
(11,12)	(5,6)	-	-	-	7.39	-	-

Comment to Tables S9–S17

Ab initio calculations were carried out using OpenMolcas quantum chemistry software package[11] and were performed on the experimental geometry of **1**, **2**, **4**, and **5** obtained from SC-XRD measurements. Only fragments of the structures consisting of $[Yb(2,2'\text{-bpdo})_4]^{3+}$ complexes were used for computations (Figure S23). The performed calculations were of the CASSCF/RASSI/SINGLE_ANISO type. Scalar relativistic effects were taken into account by employing second-order DKH (Douglas-Kroll-Hess) Hamiltonian and relativistic basis sets of an ANO-RCC type. Initially, two models with different basis sets were used: smaller with VDZP basis function quality for Yb^{III} centers (model **S**) and larger with the VTZP one (model **L**). Labels of the used basis sets and contractions for all atoms were gathered in Table S9. To save disk space Cholesky decomposition of ERI-s (electron repulsion integrals) was used with the $1.0\cdot10^{-8}$ threshold. In the first step, state average multiconfigurational self-consistent field (CASSCF) calculations for 7 doublets of ^2F term of Yb(III) were carried out. The active space was constructed of seven 4f-orbitals of Yb(III) with 13 active electrons CAS(13in7). To investigate the impact of additional orbital mixing on oscillator strengths calculations a larger active space was considered. The RASSCF scheme consisted of three 5p orbitals with 6 electrons in RAS1, seven 4f orbitals with 13 electrons in RAS2, and five 5d orbitals in RAS3. Within RAS2 complete active space calculation is performed, while in RAS1 two holes and RAS3 two excitations were allowed. Such active space can be labeled as CAS(19in15) and was employed for one of the complexes in **1** (model **L+**). In the following part, all 7 optimized spin-free states were mixed by the spin-orbit coupling within RASSI (Restricted Active Space State Interaction) program resulting in 14 spin-orbit states. At the final stage, the SINGLE_ANISO module[12,13] was applied to: (i) decompose spin-orbit states into states with a definite projection of the total momentum on the quantization axis, (ii) calculate pseudo-g-tensors for each Kramers doublet, and (iii) simulate *dc* magnetic curves, $M(H)$ and $\chi_M T(T)$. Each obtained energy splitting together with components of pseudo-g-tensors and composition of ground Kramers doublets in $|J = 7/2, m_J\rangle$ basis is presented in Tables S10–S15. The comparison of simulated $M(H)$ and $\chi_M T(T)$ dependencies with experimental ones can be found in Figures S9–S10.

The strong admixture of different $|m_J\rangle$ states and non-vanishing transversal components (g_x and g_y) of ground Kramers doublets in all analogs clearly explains the lack of SMM behavior under zero *dc* field due to fast Quantum Tunneling of Magnetization (QTM). Only after applying the external *dc* field, the QTM effect can be quenched and slow magnetic relaxation is observed. Moreover, in all calculated cases the first excited state is located at much higher energy than the expected effective energy barrier typical for the Orbach process estimated using the Arrhenius law, which is a common phenomenon for Yb^{III}-based SMMs.[14] To rationalize the gathered high-resolution emission spectra, correctly assign each band to transitions between the given states and gain insight into relative intensities of them, the following method is proposed. Taking advantage of a very precise knowledge of the electronic structure obtained from relativistic *ab initio* calculation it is possible to calculate (within the RASSI module of OpenMolcas) transition moments between spin-orbit states given after diagonalization of AMFI spin-orbit Hamiltonian. Therefore, we can obtain oscillator strength from state A to B, which can be seen as factors expressing the probability of emission, thus also band intensity, defined as follows:

$$f_{AB} = \frac{2m_e \, |\langle A|W|B\rangle|^2}{3\hbar^2(E_B - E_A)} \qquad \text{(eq3)}$$

where E_B and E_A are energies of state B and A, respectively, m_e is an electron mass and $\langle A|W|B\rangle$ is a transition moment between these two states. In the proposed method two main approximations are applied. Firstly, here the used states are from state average multiconfigurational calculations taking into account relativistic effects but done on experimental geometry, and therefore the geometry for the excited electronic states was not optimized. Furthermore, higher-order transitions moments (f. ex. electric-quadrupole or magnetic-dipole) were omitted and only electric-dipole transitions were taken into account. Nevertheless, both of these can be justified. The application of the first one can be explained due to a small impact of changing geometry on the crystal field for well-screened valence electrons of lanthanides ions, while the second one is well established in very successful theories such as the Judd-Ofelt Theory of intensities.[15,16] When in 1936 Van Vleck firstly ascribed sharp emission lines of rare-earth metal ions to parity (Laporte) forbidden f-f electronic transitions, he tried to justify them by electric-quadrupole and magnetic-dipole mechanism of transitions combined with crystal field distortions during vibrations.[17] However, it was later shown that electric-dipole transitions are sufficient to explain intensities, but one must consider admixture of a configuration of other parity f. ex. due to odd terms in the expansion of the crystal

field potential.[18] For A and B states which are exact solutions to time-independent Schrodinger equation with Hamiltonian, H, and for arbitrary operator W, simple equality holds:

$$\langle A|W|B\rangle = \frac{\langle A|[W,H]|B\rangle}{E_{\mathrm{B}}-E_{\mathrm{A}}} \qquad \text{(eq4)}$$

According to this, there exist an arbitrary choice of what operator is valid to use. To calculate dipole transition moments, where operator W is simply a vector of (x, y, z) coordinates, we can also use [W, H], which for non-relativistic Hamiltonian is a velocity operator. As far as the used functions are exact solutions, the two approaches are strictly the same. However, if we use approximated functions, as in every practical calculation, those two can differ significantly. Over the past years, many debates occurred about which operator and in which cases would be correct.[19,20] Here, we found the velocity gauge to more correctly reproduce experimental spectra. The calculated cumulative oscillator strengths which are obtained by 2x2 summation over degenerated Kramers doublets of energy states A and B in **L**/**L+** models were presented in Table S17. They are compared with experimental high-resolution emission spectra measured at 80 K (Figures 4 and S26) and represented by bars with their height normalized to the strongest transition intensity. Also, the most intense hot transitions from the states labeled as (11,12), first excited states of $^2F_{5/2}$ term, were assigned to experimental data. They are presented as bars with negative energy. The experimental emission spectra appeared to be well-reproduced by the results of calculations following the proposed method.

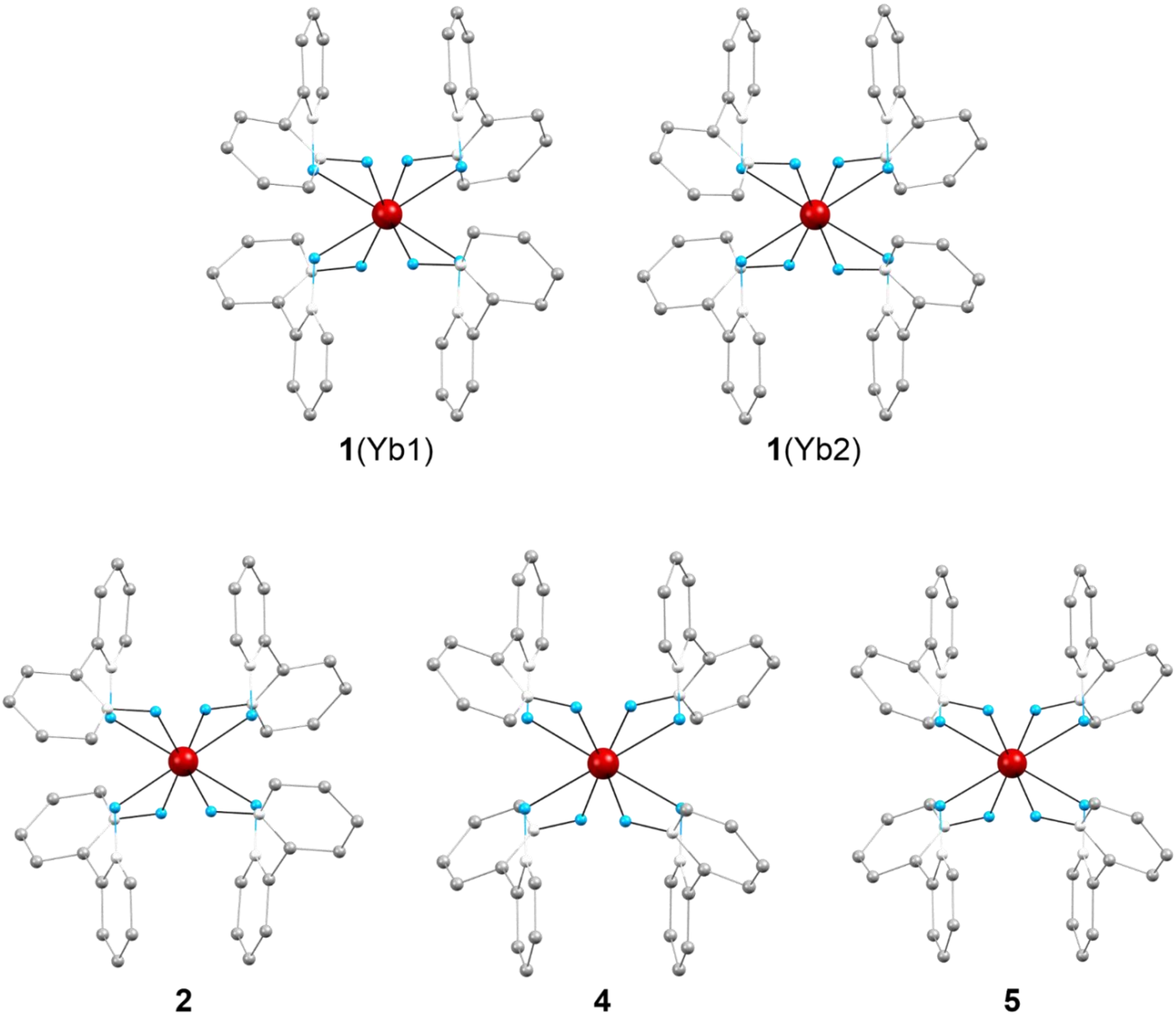

Figure S23. Selected fragments of the crystal structures of **1**, **2**, **4**, and **5** used for *ab initio* calculations of the Yb^{III} crystal field.

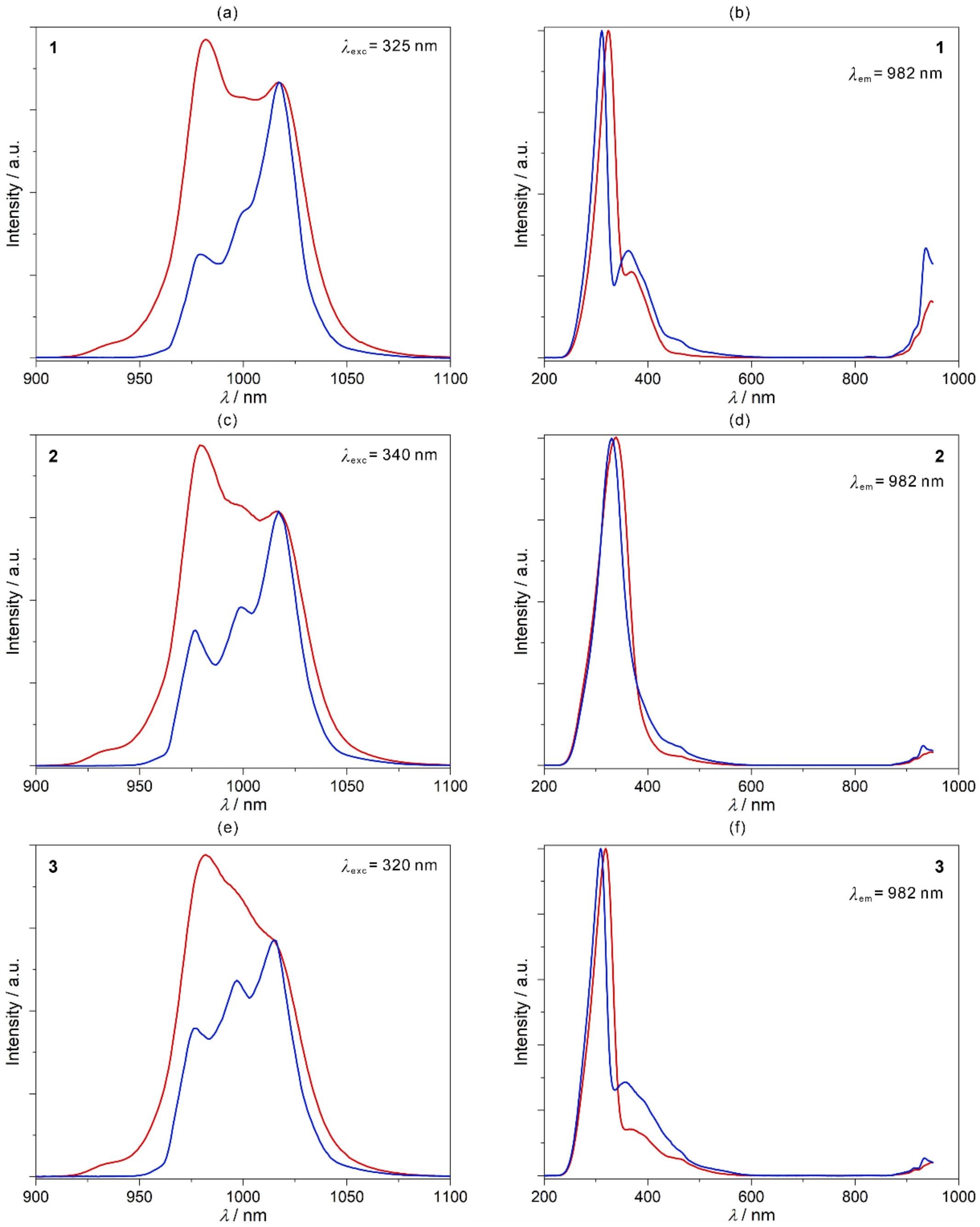

Figure S24. Solid-state excitation (left panel) and emission (right panel) spectra of **1** (a, b), **2** (c, d), and **3** (e, f) gathered at 80 K (blue solid lines) and 300 K (red solid lines). The respective emission and excitation wavelengths used were indicated in the figures.

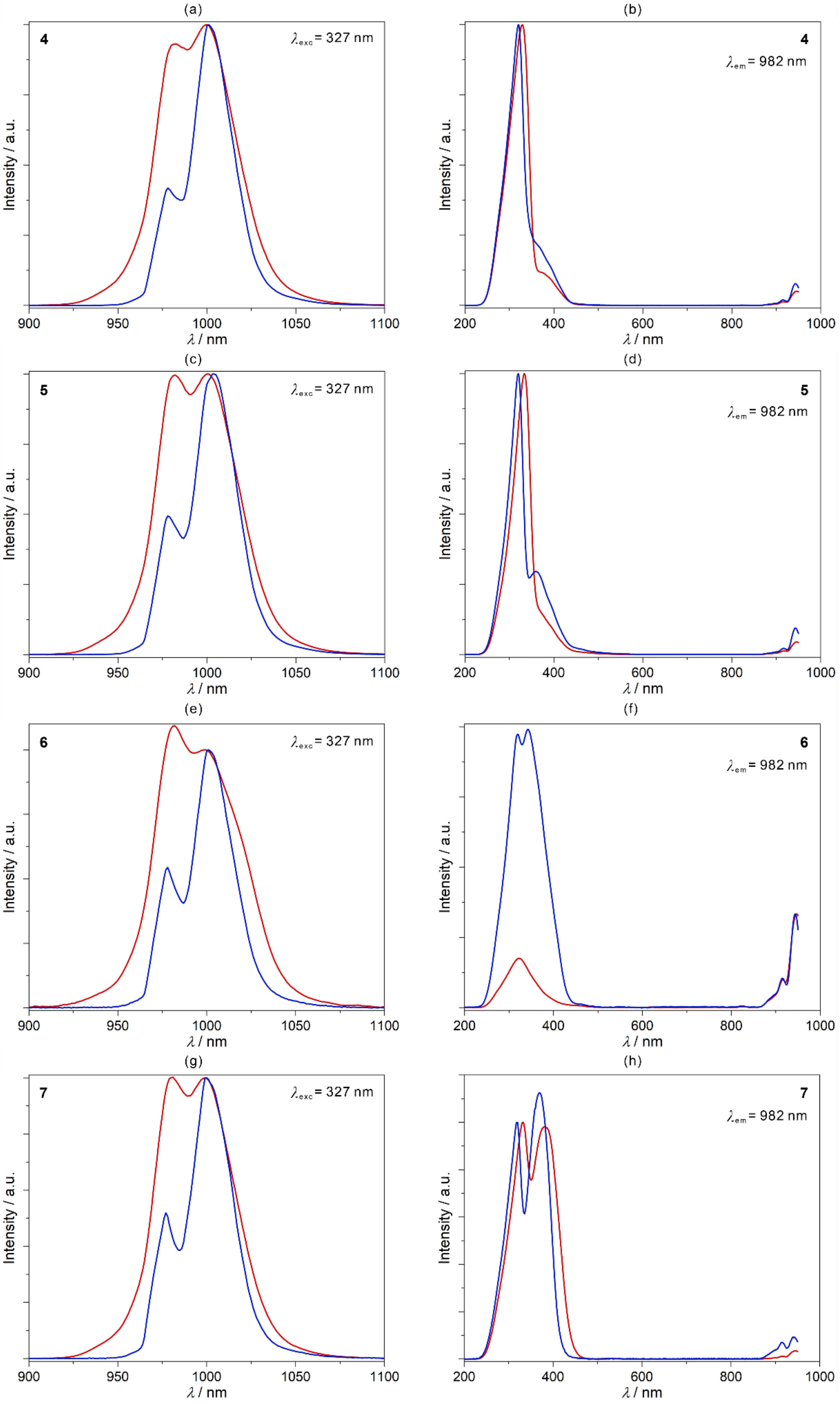

Figure S25. Solid-state excitation (left panel) and emission (right panel) spectra of **4** (a, b), **5** (c, d), **6** (e, f), and **7** (g, h) gathered at 80 K (blue solid lines) and 300 K (red solid lines). The respective emission and excitation wavelengths used were indicated in the figures.

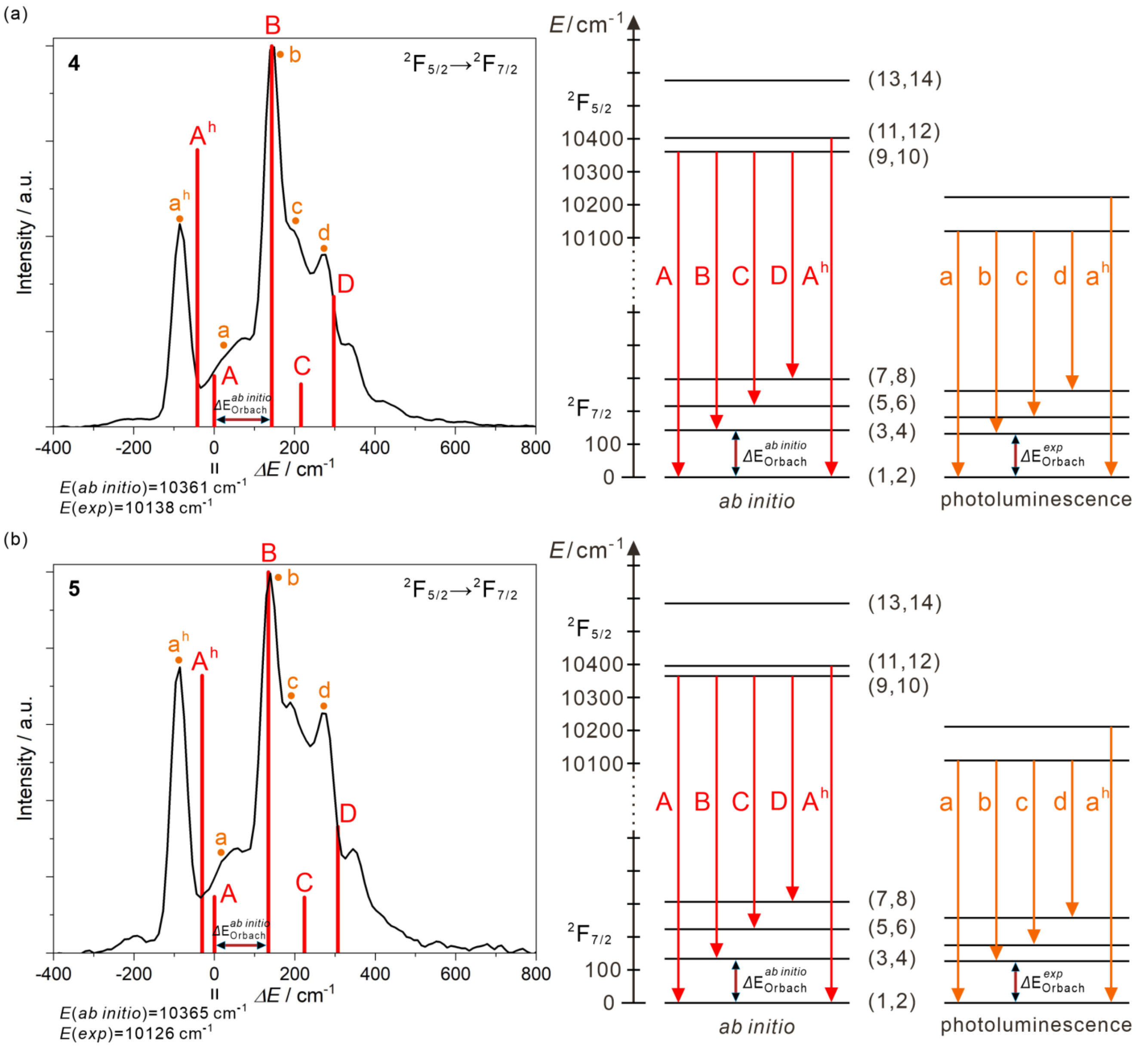

Figure S26. High-resolution emission spectra of **4** (a) and **5** (b) at 80 K for the 325 nm excitation (black lines), shown with the calculated cumulative oscillator strengths (colored bars) obtained from the *ab initio* calculations using the **L** models. The spectra are presented in the function of energy differences (*ΔE*) counted in relation to the calculated 0–0 emission line (A). For better comparison, the spectra were re-positioned to have the identical zero point and the corresponding absolute energy values are indicated. The positive values of *ΔE* represent the transition energies smaller than the 0–0 line, thus they directly relate to the energy splitting of the ground multiplet, while the negative values of *ΔE* show the hot bands (marked with *h*). The right panel shows the comparison of the energy level diagrams for Yb^{III} centers obtained from the *ab initio* and the emission spectra. The energy barriers for potential Orbach magnetic relaxation were indicated.

Table S18. Comparison of experimental and theoretical (obtained from the *ab initio* calculations) energies of emission transitions and energies of Kramers doublets of Yb^{III} complexes in **1**, **2**, **4**, and **5** (see Figures 5 and S26).

compound	1				2		4		5	
Yb center	Yb1		Yb2		Yb1		Yb1		Yb1	
energy of transition / cm^{-1}	*ab initio*	*exp*	*ab initio*	*exp*	*ab initio*	*exp*	*ab initio*	*exp*	*ab initio*	*exp*
A (*ab initio*) a (*exp*) ″0–0 line″ (ΔE value, Fig. 5, S26)*	10350.0 (0)	10133.3 (-12.1)	10348.6 (1.4)	10087.0 (34.2)	10355.0 (0)	10131.2 (0)	10360.7 (0)	10119.8 (18.1)	10364.7 (0)	10108.8 (17.2)
B (*ab initio*) b (*exp*) (ΔE value)	10211.2 (138.7)	10011.2 (110.0)	10169.2 (180.8)	9935.8 (185.3)	10202.1 (152.9)	10006.4 (124.8)	10218.0 (142.7)	9988.0 (149.9)	10230.9 (133.8)	9982.2 (143.8)
C (*ab initio*) c (*exp*) (ΔE value)	10094.7 (255.2)	9855.8 (265.4)	10083.6 (275.1)	9824.6 (296.6)	10070.0 (285.0)	9814.8 (316.7)	10145.2 (215.5)	9937.8 (200.1)	10141.4 (223.3)	9933.9 (192.1)
D (*ab initio*) d (*exp*) (ΔE value)	9958.2 (391.7)	9714.4 (406.8)	9951.7 (398.3)	9680.6 (440.6)	9947.7 (407.3)	9693.2 (438.3)	10063.6 (297.1)	9858.3 (279.6)	10058.2 (306.5)	9850.9 (275.1)
A^h (*ab initio*) a^h (*exp*) (ΔE value)	10432.1 (-82.1)	10226.2 (-92.9)	10467.7 (-117.7)	10240.9 (-119.7)	10450.2 (-95.2)	10236.9 (-105.7)	10402.6 (-41.9)	10222.9 (-85.0)	10395.5 (-30.8)	10211.1 (-85.1)
C^h (*ab initio*) c^h (*exp*) (ΔE value)	-	-	-	-	10165.2 (189.8)	9936.7 (194.5)	-	-	-	-
energy of pseudo doublet / cm^{-1}	*ab initio*	*exp*	*ab initio*	*exp*	*ab initio*	*exp*	*ab initio*	*exp*	*ab initio*	*exp*
(1,2)	**0**	**0**	**0**	**0**	**0**	**0**	**0**	**0**	**0**	**0**
(3,4)	**138.7**	**122.1**	**179.4**	**155.1**	**152.9**	**124.8**	**142.7**	**131.8**	**133.8**	**126.6**
(5,6)	**255.2**	**277.5**	**273.7**	**262.4**	**285.0**	**316.7**	**215.5**	**182.0**	**223.3**	**174.9**
(7,8)	**391.7**	**418.9**	**396.9**	**406.4**	**407.3**	**438.3**	**297.1**	**261.5**	**306.5**	**257.9**
(9,10)	10350.0	10133.3	10348.6	10087.0	10355.0	10131.2	10360.7	10156.0	10364.7	10108.8
(11,12)	10432.1	10226.2	10467.7	10240.9	10450.2	10236.9	10402.6	10222.9	10395.5	10211.1
(13,14)	10655.7	-	10659.8	-	10677.6	-	10575.3	-	10584.9	-
ΔE_{Orbach} [energy of the (3,4) doublet]	**138.7**	**122.1**	**179.4**	**155.1**	**152.9**	**124.8**	**142.7**	**131.8**	**133.8**	**126.6**

*Please notice that, for **1**, $E(exp)$ = 10121.1 cm^{-1} relates to the $\Delta E = 0$ in the scale for the experimental data while E(*ab initio*) = 10350.0 cm^{-1} relates to the $\Delta E = 0$ in the scale for the computed data (Figure 5a). For **2**, $E(exp)$ = 10131.2 cm^{-1} and E(*ab initio*) = 10355.0 cm^{-1} relate to the $\Delta E = 0$ (Figure 5b). For **4**, $E(exp)$ = 10137.9 cm^{-1} and E(*ab initio*) = 10360.7 cm^{-1} relate to the $\Delta E = 0$ (Figure S26a). For **5**, $E(exp)$ = 10126.0 cm^{-1} and E(*ab initio*) = 10364.7 cm^{-1} relate to the $\Delta E = 0$ (Figure S26b).

REFERENCES to SUPPORTING INFORMATION

(1) Ziegler, B.; Babel, D. Die Kristallstruktur des Cyanospinells $K_2Cd(CN)_4$. *Z. Naturforsch.* **1991**, *46b*, 47–49.

(2) Watt, G. W.; Helvenston, E. P.; Sharif, L. E. Ammines of Iridium(0). *J. Inorg. Nucl. Chem.* **1962**, *24,* 1067–1072.

(3) Sheldrick, G. M. *SHELXS-97, Program for Crystal Structure Solution;* University of Göttingen: Göttingen, 1997.

(4) Sheldrick, G. M. SHELXT – Integrated Space-Group and Crystal-Structure Determination. *Acta Crystallogr., Sect. A: Found. Adv.* **2015**, *71*, 3–8.

(5) Sheldrick, G. M. Crystal Structure Refinement with *SHELXL*. *Acta Cryst.* **2015**, *C71*, 3–8.

(6) Farrugia, L. J. *WinGX* and *ORTEP for Windows*: an Update. *J. Appl. Crystallogr.* **2012**, *45*, 849–854.

(7) Llunell, M.; Casanova, D.; Cirera, J.; Bofill, J.; Alemany, P.; Alvarez, S.; Pinsky, M.; Avnir, D. *SHAPE v. 2.1, Program for the Calculation of Continuous Shape Measures of Polygonal and Polyhedral Molecular Fragments*; University of Barcelona: Barcelona, 2013.

(8) Casanova, D.; Cirera, J.; Llunell, M.; Alemany, P.; Avnir, D.; Alvarez, S. Minimal Distortion Pathways in Polyhedral Rearrangements. *J. Am. Chem. Soc.* **2004**, *126*, 1755–1763.

(9) Guo, Y.-N.; Xu, G.-F.; Guo, Y.; Tang, J. Relaxation Dynamics of Dysprosium(III) Single Molecule Magnets. *Dalton Trans.* **2011**, *40*, 9953–9963.

(10) Ramos Silva, M.; Martin-Ramos, P.; Coutinho, J. T.; Pereira, L. C. J.; Martin-Gil, J. Effect of the Capping Ligand on Luminescent Erbium(III) β-Diketonate Single-Ion Magnets. *Dalton Trans.* **2014**, *43*, 6752–6761.

(11) Galván, I. F.; Vacher, M.; Alavi, A.; Angeli, C.; Aquilante, F.; Autschbach, J.; Bao, J. J.; Bokarev, S. I.; Bogdanov, N. A.; Carlson, R. K., et al. OpenMolcas: From Source Code to Insight. *J. Chem. Theory Comput.* **2019**, *15*, 5925–5964.

(12) Chibotaru, L. F.; Ungur, L. Ab Initio Calculation of Anisotropic Magnetic Properties of Complexes. I. Unique Definition of Pseudospin Hamiltonians and Their Derivation. *J. Chem. Phys.* **2012**, *137*, 064112.

(13) Ungur, L.; Chibotaru, L. F. Ab Initio Crystal Field for Lanthanides. *Chem. Eur. J.* **2017**, *23*, 3708–3718.

(14) Wang, J.; Zakrzewski, J. J.; Heczko, M.; Zychowicz, M.; Nakagawa, K.; Nakabayashi, K.; Sieklucka, B.; Chorazy, S.; Ohkoshi, S. Proton Conductive Luminescent Thermometer Based on Near-Infrared Emissive $\{YbCo_2\}$ Molecular Nanomagnets. *J. Am. Chem. Soc.* **2020**, *142*, 3970–3979.

(15) Judd, B. R. Optical Absorption Intensities of Rare-Earth Ions. *Phys. Rev.* **1962**, *127*, 750–761.

(16) Ofelt, G. S. Intensities of Crystal Spectra of Rare-Earth Ions. *J. Chem. Phys.* **1962**, *37*, 511–520.

(17) Van Vleck, J. H. The Puzzle of Rare-Earth Spectra in Solids. *J. Phys. Chem.* **1937**, *41*, 67–80.

(18) Peacock, R. D. The Intensities of Lanthanides f-f Transitions. *Struct. Bond.* **1975**, *22*, 83–122.

(19) Roginsky, D. V. I.; Klapisch, M.; Cohen, M. Electric Dipole Oscillator Strengths: Length and Velocity! *Chem. Phys. Lett.* **1983**, *95*, 568–572.

(20) Starace, A. F. Length and Velocity Formulas in Approximate Oscillator-Strength Calculations. *Phys. Rev. A* **1971**, *3*, 1242–1245.
